# Comparative genomic analysis of *Genlisea* (corkscrew plants—Lentibulariaceae) chloroplast genomes reveals an increasing loss of the *ndh* genes

**DOI:** 10.1371/journal.pone.0190321

**Published:** 2018-01-02

**Authors:** Saura R. Silva, Todd P. Michael, Elliott J. Meer, Daniel G. Pinheiro, Alessandro M. Varani, Vitor F. O. Miranda

**Affiliations:** 1 Universidade Estadual Paulista (Unesp), Botucatu, Instituto de Biociências, São Paulo, Brazil; 2 J. Craig Venter Institute, La Jolla, CA, United States of America; 3 10X Genomics, Pleasanton, California, United States of America; 4 Universidade Estadual Paulista (Unesp), Faculdade de Ciências Agrárias e Veterinárias, Jaboticabal, Departamento de Tecnologia, São Paulo, Brazil; 5 Universidade Estadual Paulista (Unesp), Faculdade de Ciências Agrárias e Veterinárias, Jaboticabal, Departamento de Biologia Aplicada à Agropecuária, São Paulo, Brazil; Agriculture and Agri-Food Canada, CANADA

## Abstract

In the carnivorous plant family Lentibulariaceae, all three genome compartments (nuclear, chloroplast, and mitochondria) have some of the highest rates of nucleotide substitutions across angiosperms. While the genera *Genlisea* and *Utricularia* have the smallest known flowering plant nuclear genomes, the chloroplast genomes (cpDNA) are mostly structurally conserved except for deletion and/or pseudogenization of the NAD(P)H-dehydrogenase complex (*ndh*) genes known to be involved in stress conditions of low light or CO_2_ concentrations. In order to determine how the cpDNA are changing, and to better understand the evolutionary history within the *Genlisea* genus, we sequenced, assembled and analyzed complete cpDNA from six species (*G*. *aurea*, *G*. *filiformis*, *G*. *pygmaea*, *G*. *repens*, *G*. *tuberosa* and *G*. *violacea*) together with the publicly available *G*. *margaretae* cpDNA. In general, the cpDNA structure among the analyzed *Genlisea* species is highly similar. However, we found that the plastidial *ndh* genes underwent a progressive process of degradation similar to the other terrestrial Lentibulariaceae cpDNA analyzed to date, but in contrast to the aquatic species. Contrary to current thinking that the terrestrial environment is a more stressful environment and thus requiring the *ndh* genes, we provide evidence that in the Lentibulariaceae the terrestrial forms have progressive loss while the aquatic forms have the eleven plastidial *ndh* genes intact. Therefore, the Lentibulariaceae system provides an important opportunity to understand the evolutionary forces that govern the transition to an aquatic environment and may provide insight into how plants manage water stress at a genome scale.

## Introduction

The carnivorous plant *Genlisea* has astonished scientists for many years. Charles Darwin was seduced by this “remarkable genus” which he described at the end of his book Insectivorous Plants [1]. The genus *Genlisea* A.St.-Hil. belongs to the carnivorous family Lentibulariaceae together with genera *Utricularia* and *Pinguicula* [2]. *Genlisea* encompass about 30 species that inhabit open areas with nutrient-poor soil distributed in tropical Africa and the Neotropics (eight of twenty species are endemic to Brazil) [3–6]. *Genlisea* are small, rootless, terrestrial herbs commonly known as “corkscrew plants” due to Y-shaped-underground leaves that are twisted helically and have the ability to capture, digest and absorb prey [7,8]. It is difficult to distinguish different species based solely on the vegetative forms due to *Genlisea* having a diverse set of intraspecific phenotypes. Despite Darwin’s early interest however, *Genlisea* remains poorly studied due to cultivation challenges, and being found in isolated and remote habitats [9].

*Genlisea* and *Utricularia* have one of the highest nucleotide substitution rates across all three genome compartments (nucleus, chloroplast, mitochondria) in comparison to other angiosperms [10–12] with previous studies revealing that both genera have an exclusive mutation in the mitochondrial cytochrome *c* oxidase gene (*cox1*) [13]. These mutations lead to a proton pumping change and, during oxidative phosphorylation, cause electrons to leak into the mitochondria, generating reactive oxygen species (ROS). It is proposed that the ROS can damage DNA, which produces breaks in the double helix structure, leading to point mutations [14–16]. On an evolutionary timescale this potential increase in ROS could explain the high nucleotide substitution rate, the process of genome miniaturization [17], and a high diversification of morphological traits [14].

Previous systematic studies were carried out using morphological traits, mainly based on capsule dehiscence together with trap, pollen, flower characteristics [4,18,19] and molecular markers from the three plastidial loci: *trnK/matK*, *rps16* and *trnQ-rps16*. Phylogenies based on these markers suggested two major groups within *Genlisea*: the subgenus *Genlisea*, comprising the sections *Genlisea*, *Africanae* and *Recurvatae*, and the subgenus *Tayloria*. However, due to the recent discovery of new species, unresolved clades and possible cryptic species, the evolutionary history of *Genlisea* requires further investigation [4,18].

Chloroplast genome (cpDNA) sequencing and analysis of different species provides a powerful tool to dissect out the evolutionary history of plant genera. The highly conserved structure and gene content of the cpDNA enable plant evolution and phylogeny studies [20]. Structural rearrangements, gene decay and loss are often observed in cpDNA and inform a plethora of evolutionary relationships among different taxa. For example, plastid gene loss in the most extreme cases is linked to lineages with heterotrophic nutrition, such as parasitic [21] and mycoheterotrophic plants [22].

One of the gene losses that occur in such plants is related to the NAD(P)H-dehydrogenase complex (*ndh*) genes. The *ndh* genes consist of eleven (11) subunits in the cpDNA (*ndhA*, *B*, *C*, *D*, *E*, *F*, *G*, *H*, *I*, *J* and *K*) that encodes, along with nuclear genes, the thylakoid NAD(P)H dehydrogenase complex [23]. This complex is involved in photosynthesis, the photosynthetic response and stress acclimation [24], and has been hypothesized to be related to the transition to terrestrial habitats [14,16]. The eleven *ndh* subunit genes are present in the aquatic Lentibulariaceae species, but are lost in the terrestrial *Utricularia* species, suggesting that the evolutive history of the *ndh* genes among the *Utricularia* lineages followed an opposite trend, and that the *ndh* function may be dispensable in terrestrial forms [25]. However the presence and absence of the *ndh* genes remain to be established in *Genlisea* species. Therefore, the *ndh* genes in the cpDNA can provide a valuable resource for the understanding of *Genlisea* evolution and how these genes can be associated to the habitats.

To better understand the evolutionary history of the *Genlisea* genus and explore the role of *ndh* gene loss, we sequenced, assembled six chloroplast genomes and, together with the published *G*. *margaretae* cpDNA, carried out a full analysis. These seven *Genlisea* species represent both subgenera *Tayloria* (*G*. *violacea*) and *Genlisea (G*. *aurea*, *G*. *filiformis*, *G*. *pygmaea*, *G*. *repens*, *G*. *tuberosa* and *G*. *margaretae)*. We found that the chloroplast genome is highly similar across species, but unlike their aquatic relatives, in the terrestrial *Genlisea* species the *ndh* genes are deleted, fragmented or pseudogenized. These findings not only add to the understanding of terrestrial heterotrophic plants, and their cpDNA evolution, but also provide an important opportunity to understand the evolutionary forces that govern the transition to an aquatic environment at a genome scale.

## Material and methods

### Plant samples, preparation and sequencing

Fresh photosynthetic leaves of *Genlisea* species were sampled from natural populations and also cultivated and stored in silica gel. Total DNA was extracted using modified CTAB protocol and concentration, integrity and purity was assessed using Nanodrop^TM^ spectrophotometer (Thermo Scientific) and Agilent 2100 Bioanalyzer (Agilent Genomics). Herbarium vouchers are deposited at the Herbarium JABU at Universidade Estadual Paulista (UNESP/FCA; ICMBio/ MMA for collecting permits SISBIO #26938 and #48516) (S1 Table).

The paired-end libraries were prepared using Illumina library preparation manufacturer’s protocol and genomic DNA was sequenced using Illumina Miseq Platform (Illumina, San Diego, CA).

The publicly available *Genlisea aurea* DNA sequencing data was obtained from raw genome database SRA (accession number SRR916071) that was previously used for nuclear genome assembly [26].

### Assembly and annotation

The quality of raw reads was assessed by FastQC [27]. Removal of adapters from both ends and trimming to obtain high quality reads were performed using the Platanus_trim (v.1.0.7) [28] with Phred quality score of >30 and length cutoff of 150bp for 300bp reads, 100bp for 150bp reads, 80bp for 100bp reads and 50bp for 75bp reads (see S1 Table). In addition, to exclude nuclear and mitochondrial genomes, the *Genlisea* species chloroplast genome paired end reads were extracted by mapping all raw reads to the reference cpDNA *Utricularia gibba* (NC021449) with Bowtie2 (v.2.2.3) [29] (i.e.–very-sensitive-local with–N 1 modification). Then this selected set of reads was assembled using Spades (v.3.7.1) [30] software with default parameters. Uncertain regions, such as IR junctions, were picked out from published Lentibulariaceae species (*U*. *gibba* and *Genlisea margaretae* [NC025652.1]) to extend the length using iteration method with MITObim (v.1.8) [31]. As the assembly usually collapses the inverted repeats in one single contig, the IR region of some species were manually inverted and duplicated to integrate the whole chloroplast genome using BioEdit (v.7.2) [32]. High quality filtered reads were mapped back using Bowtie2 (i.e.–very-sensitive; end-to-end) in Geneious Pro (v.10.2.3) [33] to each assembled chloroplast genome to confirm assembly accuracy quality and repeat region junctions (S1 Table; S1 Fig).

The annotation of the chloroplast genomes were performed using Dual Organellar GenoMe Annotator (DOGMA) [34] with manual corrections for start and stop codons and intron boundaries by comparison to homologous genes from sequenced chloroplast of *Utricularia gibba*, *U*. *reniformis* (NC029719.2) and *Genlisea margaretae*. The tRNA genes were also verified with ARAGORN [35] and tRNAscan-SE [36]. The codon usage was calculated using CodonW (v1.4.4) [37].The circular chloroplast genome maps were drawn using OrganellarGenome DRAW tool (OGDRAW) [38].

To determine whether a gene was a pseudogene, fragmented or deleted gene, Blastn and Blastx searches were performed using other chloroplasts as reference, such as *U*. *gibba*, and a pseudogene was characterized according to the absence of start and/or stop codon, frameshift and genes with more than 20% of the coding region in comparison to other related species. The genes that are considered as fragmented were any group of nucleotides that had at least >25bp and had correspondence to position and blastn and tblastx alignment with the complete gene.

### Repeat identification

REPuter [39] was used to search both direct and palindrome sequences, with a minimum repeat size of > 30bp and a sequence identity greater than 90% (parameters: repfind–f–p–l 30 –h 3 –best 10,000). Microsatellites for mono-, di-, tri-, penta- and hexanucleotides were detected using the Perl script MISA [40]. The established parameters were performed according with Silva *et al*. [25].

### Identity and variation analyses

The chloroplast genomes were aligned using MAFFT (v.7) [41] with FFT-NS-2 parameters and identity comparisons between chloroplasts were conducted with mVISTA program [42].

Average p-distances were calculated to determine genetic divergence between *Genlisea* species and the number of phylogenetically informative characters (PICs) for each plastome gene, intergenic spacers, introns and pseudogenes using PAUP (v.4b10) [43]. Nonparametric Spearman test was used to test for correlation between PICs and average p-distances between sequences of *Genlisea* species.

### Phylogenomic analyses

Phylogenetic analyses were performed to different partitions by using the whole chloroplast genome sequence, protein coding genes, intergenic spacers, LSC (Large Single Copy), SSC (Small Single Copy), IR (Inverted Repeat) and *ndh* genes. For *ndh* phylogenetic tree, pseudogenes and fragments of deleted genes of at least 25bp were considered (S2 and S3 Tables). Previously published Lentibulariaceae chloroplast genomes were included (*Utricularia foliosa* [KY025562], *U*. *gibba* [NC021449], *U*. *macrorhiza* [NC025653], *U*. *reniformis* [NC029719.2] and *Pinguicula ehlersiae* [NC023463]) and *Tectona grandis* (Lamiaceae) [NC020098], *Sesamum indicum* (Pedaliaceae) [NC016433] and *Tanaecium tetranolobum* (Bignoniaceae) [NC027955] cpDNA used as outgroup.

The alignments were conducted using MAFFT (v.7) [41] and the evolutionary model (best-of-fit) that was most appropriate for all the data according with corrected Akaike Information Criterion (AICc), calculated using jModelTest [44].

Maximum parsimony criterion was performed using PAUP (v.4b10) [43] with heuristic searches of 2,000 replicates and bootstrap analysis with 1,000 pseudoreplicates, both using the tree bisection-reconnection branch swapping (TBR) and random addition of sequences. The probabilistic analysis was conducted using RAxML (v.8) [45] for maximum likelihood (ML) using the default parameters with bootstrap support of 1,000 pseudoreplicates and MrBayes (v.3) [46] for Bayesian inference with 5×10^5^ generations with two runs and four chains following the substitution matrix assessed as mentioned above. Both analyses were performed on CIPRES Science Gateway website [47] and cladograms were edited with the program TreeGraph2 (beta v.2.0) [48].

In an attempt to test also the phylogenetic signal of *ndh* genes in *Genlisea* lineages, we created a matrix with 22 characters. The characters 1 to 11 we codified if each *ndh* gene (*ndhA*, *ndhB*, *ndhC*, *ndhD*, *ndhE*, *ndhF*, *ndhG*, *ndhH*, *ndhI*, *ndhJ*, and *ndhK*) was absent (0) or present (1) and the characters 12 to 22 if each gene was pseudogenized (0), decayed (1) or complete (2) for each of the eleven *ndh* genes and carried out a parsimony analysis (S2–S4 Tables). The consensual tree (strict consensus) of most parsimonious trees was presented and evolution of *ndh* genes was traced using both matrix and chloroplast phylogenomic tree described above with PAUP (v.4b10) with ACCTRAN optimization [43].

## Results

### Genome content and organization of the six *Genlisea* chloroplast genomes

**Fig 1:**
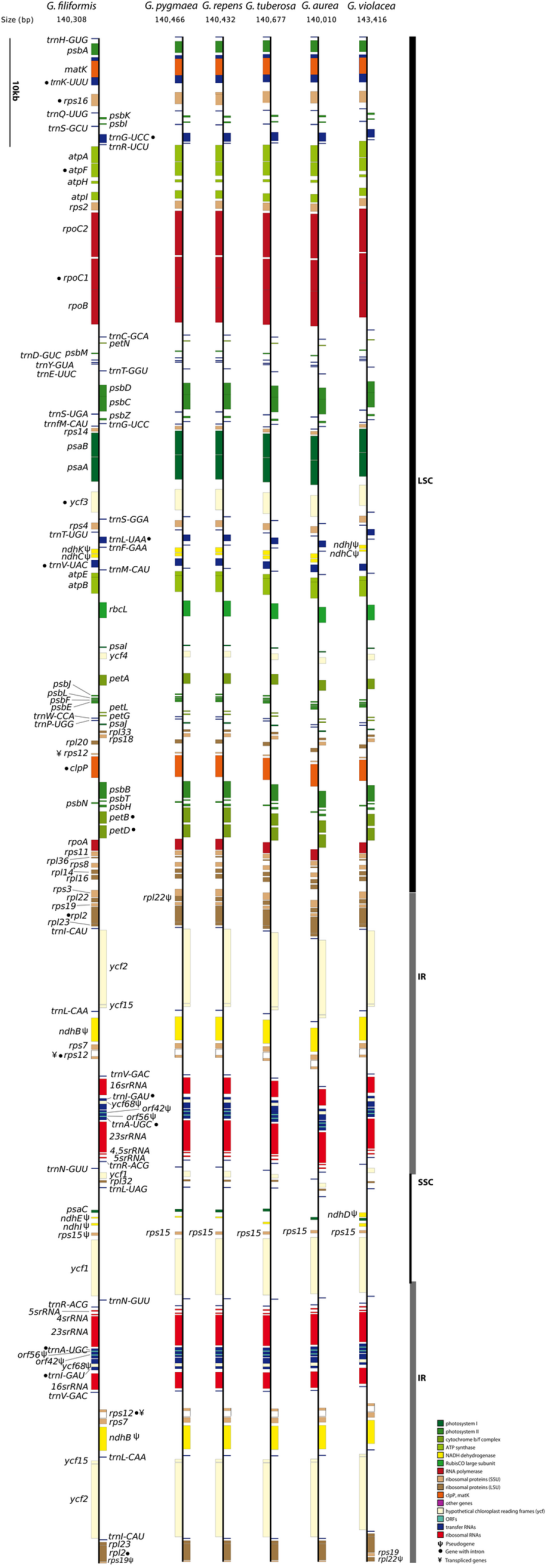
Physical chloroplast genome maps of six assembled *Genlisea* species. The chloroplast genome is showed with the genes colorized according to the functional classes for each species. The genes shown on the right side of each cpDNA map are transcribed clockwise, whereas gene on the left side are transcribed counter clockwise. The symbol Ψ after the gene name indicates that is a pseudogene, • the presence of introns and ¥ denotes transpliced genes. Large single copy (LSC), inverted repeats (IR) and single copy repeat (SSC) are represented by the black and grey bars.

The cpDNA of *Genlisea* ranged from 140,010 bp (*G*. *aurea*) to the largest plastome of the sequenced species with 143,416 bp (*G*. *violacea*) (Fig 1, Table 1). All six chloroplast genomes display a quadripartite structure, which consists of a pair of inverted repeats (IR) separated by a Large Single Copy (LSC) and a Small Single Copy (SSC) region. The plastomes contain 103 unique genes, including 69 protein-coding genes, 30 tRNAs, 4 rRNAs and the average GC content was 38.57±0.08%. Fourteen genes contain a single intron, such as *atpF*, *petB*, *petD*, *rpl16*, *rpl2*, *rpoC1*, *rps12*, *rps16*, *trnA-*UGC, *trnG*-UCC, *trnI*-GAU, *trnK*-UUU, *trnL*-UAA and *trnV*-UAC, while *clpP* and *ycf3* have two introns. The *orf42*, *orf56*, and *ycf68* genes of the IR region are pseudogenes due to lack of start and/or stop codons (Table 2).

**Table 1:** Summary of assembly data for *Genlisea* plastomes (for details about sequencing data see S1 Table).

Species	cpDNA size (bp)	LSC size (bp)	SSC size (bp)	IRs size (bp)	GC content (%)	GenBank accession number
*Genlisea aurea*	140,010	80,653	9,419	24,969	38.5	MF593121
*G*. *filiformis*	140,308	79,754	10,316	25,119	38.7	MF593122
*G*. *pygmaea*	140,466	79,888	10,346	25,116	38.6	MF593123
*G*. *repens*	140,432	79,875	10,325	25,116	38.5	MF593124
*G*. *tuberosa*	140,677	80,347	10,462	24,934	38.5	MF593125
*G*. *violacea*	143,416	81,089	10,969	25,679	38.6	MF593126

**Table 2:** Genes in the six *Genlisea* chloroplast genomes (except *G*. *margaretae*).

Category of genes	Group of gene	Name of the gene
**Self-replication**	Ribosomal RNA genes (rRNAs)	4.5S rRNA (2x), 5S rRNA (2x), 16S rRNA (2x), 23S rRNA (2x)
	Transfer RNA genes (tRNAs)	trnH-GUG, trnK-UUU●, trnQ-UUG, trnS-GCU, trnG-UCC●, trnR-UCU, trnC-GCA, trnD-GUC, trnY-GUA, trnE-UUC, trnT-GGU, trnS-UGA, trnG-UCC●, trnfM-CAU, trnS-GGA, trnT-UGU, trnL-UAA●, trnF-GAA, trnV-UAC●, trnM-CAU, trnW-CCA, trnP-UGG, trnI-CAU, trnL-CAA (2x), trnV-GAC (2x), trnI-GAU ● (2x), trnA-UGC ● (2x), trnR-ACG (2x), trnN-GUU (2x), trnL-UAG
	Small subunit of ribosomal protein	rps2, rps3, rps4, rps7 (2x), rps8, rps11, rps12● (2x) ¥, rps14, rps15**, rps16●, rps18, rps19***
	Large subunit of ribosomal protein	rpl2● (2x), rpl14, rpl16●, rpl20, rpl22*, rpl23 (2x), rpl32, rpl33, rpl36
	RNA polymerase subunit	rpoA, rpoB, rpoC1●, rpoC2
**Photosynthesis**	NADH dehydrogenase	All are ψ or deleted (see Figs 1 and 4 for each *Genlisea* species)
	Photosystem I	psaA, psaB, psaC, psaI, psaJ, ycf3●, ycf4
	Photosystem II	psbA, psbB, psbC, psbD, psbE, psbF, psbH, psbI, psbJ, psbK, psbL, psbM, psbN, psbT, psbZ
	Cytochrome b/f complex	petA, petB●, petD●, petG, petL, petN
	ATP synthase	atpA, atpB, atpE, atpF●, atpH, atpI
	Rubisco large subunit	rbcL
**Other genes**	Translation initiation factor	infA
	Maturase	matK
	Protease	clpP●
	Envelope membrane protein	cemA
	Subunit of acetyl-CoA-carboxylase	accD
	c-type cytochrome synthesis gene	ccsA
**Unknown function**	Conserved hypothetical protein	ycf1, ycf2 (2x), ycf15 (x2), ycf68 ψ (2x), orf56 ψ (2x), orf42 ψ (2x)

● Gene with intron

ψ Pseudogenes

¥ Transpliced genes.

* One of duplicated gene is partial in *G*. *violacea* and is pseudogene in *G*. *pygmaea*

**Pseudogene in *G*. *filiformis*

*** Duplicated gene in *G*. *violacea*.

Overall, all the *Genlisea* cpDNAs are highly conserved in organization and structure (Fig 1), except for the *ndh* genes that are pseudogenized, fragmented or deleted in all *Genlisea* plastomes. In addition, *G*. *violacea* has slightly expanded IR/LSC boundary genes with the duplication of intact *rps19* gene and *rpl22* as pseudogene (see S2 Fig), and the *rps15* and *rpl22* are present as pseudogenes in *G*. *filiformis* and *G pygmaea*, respectively (Fig 1).

### Repeats in the *Genlisea* plastomes

**Fig 2:**
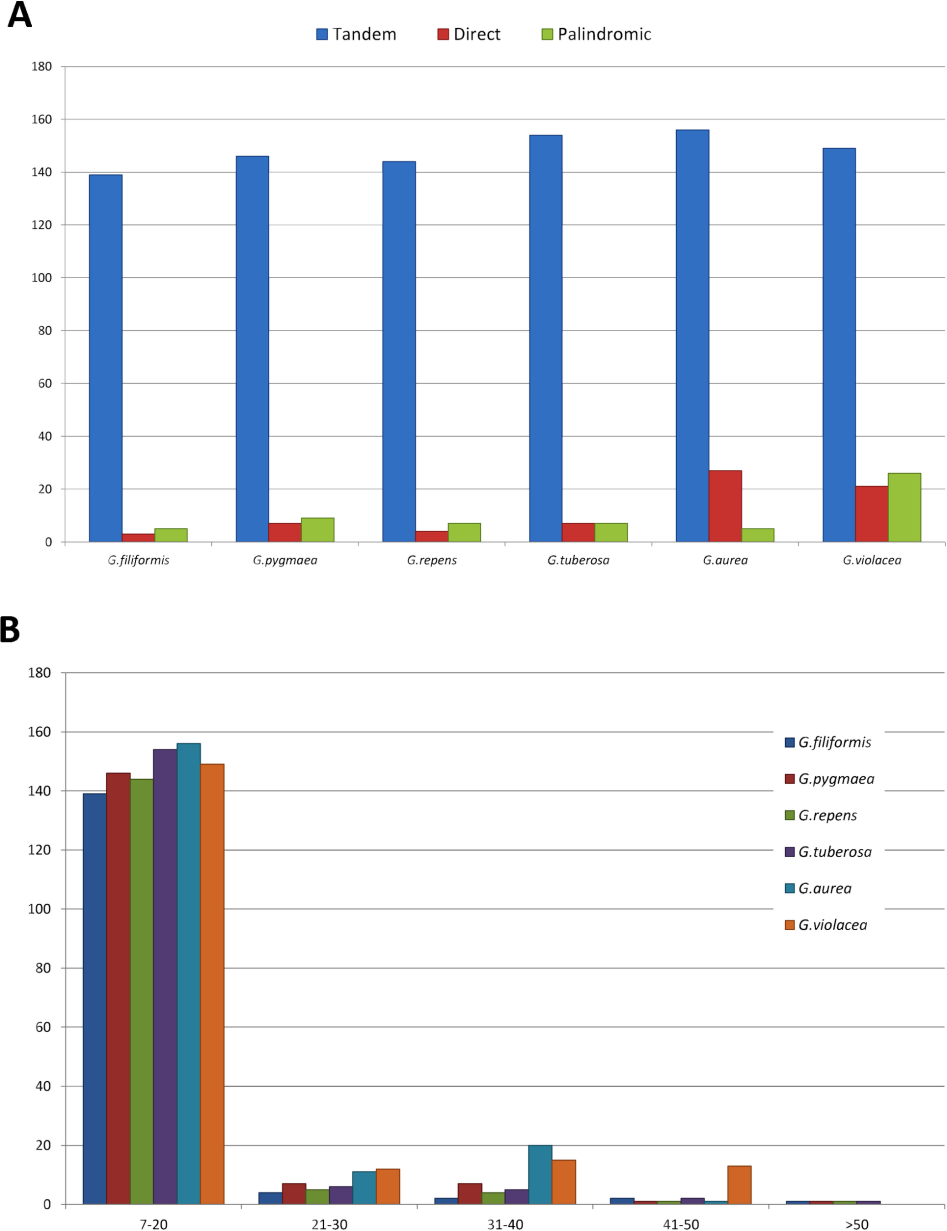
Analysis of repeats in *Genlisea* chloroplast genomes. (A) Quantity of tandem, direct and palindromic repeats of each species. (B) Quantity of repeats by length.

Repeats were divided in three categories: tandem, direct and palindromic (Fig 2). The great majority of the repeats across the chloroplast genomes were simple sequence repeats (SSRs) of lengths between 7 and 20 bp. An average of 210 repeats were detected in the six chloroplast genomes, 6.80% (69 repeats) of which are direct repeats, 5.80% (59 repeats) were palindromic repeats, and 87.40% (888 repeats) tandem repeats (Fig 2; S5 Table). Moreover, most of the repeats are located in the intergenic regions (39.40%), followed by coding (36.60%) and intronic regions (14.90%). Few repeats were found in tRNA, rRNA and pseudogenes regions (9.10%). The majority of microsatellites in all species are A/T mono- and dinucleotides. There are few tetra- and pentanucleotide and one hexanucleotide in *G*. *pygmaea*. Among all chloroplast genomes, 41 repeat regions (4%) were shared by all analyzed *Genlisea* species (S5 Table).

### Molecular markers identification

Genome wide comparison allowed the identification of genomic regions that could be used as possible phylogenetic markers to reconstruct the evolutionary history of the genus. A positive correlation between the percentage of variable sites, given by p-distance, and phylogenetically informative characters (PICs) (ρ = 0.583, P<0.001; S3 Fig) were identified. Thus, the PICs of each coding and non-coding alignment region were used to identify potential regions for phylogenetics and population studies.

**Fig 3:**
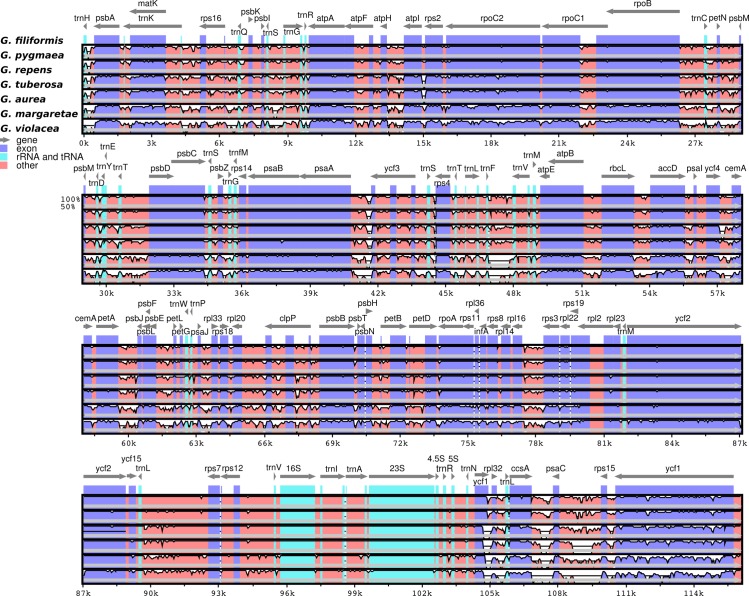
Sequence identity plots for the six assembled *Genlisea* species and previously published *G*. *margaretae*.

The divergence hotspot analysis given by p-distance and phylogenetically informative characters (S6 Table) revealed that the most informative regions for phylogenetic analyses were non-coding DNA regions such as intergenic spacers and introns (Fig 3; S4 Fig). Moreover, the p-distance between *Genlisea* and *Pinguicula* was 0.043, *Genlisea* and *Utricularia* 0.057 and between *Genlisea* species was of 0.032. The overall p-distance between *G*. *repens* and *G*. *pygmaea*, the most related species in this study, was 0.001. Phylogenetically informative characters suggest that the top ten regions with the greatest number of PICs are three genes (*ycf1*, *matK* and *rpoC2*), two introns (*rpl16-intron*, *trnK-intron*) and five intergenic regions (*trnK-rps16*, *rps12-clpP*, *petA-psbJ*, *rpl20-rps12*, *rps12-trnV*) (S4 Fig; S6 Table).

### Phylogenomic analysis

**Fig 4:**
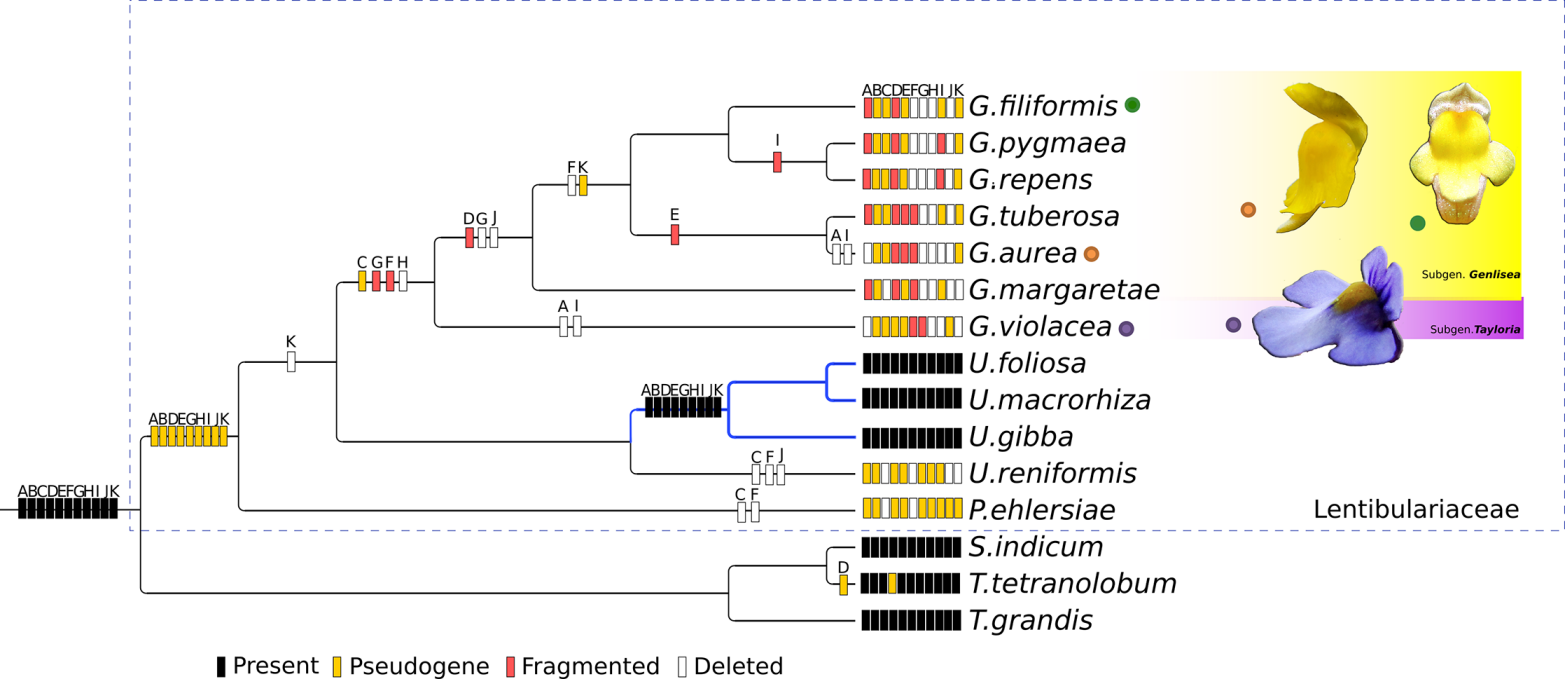
Phylogenomics of whole chloroplasts of *Genlisea* species and *ndh* genes evolution. The boxes indicate the *ndhA*, *ndhB*, *ndhC*, *ndhD*, *ndhE*, *ndhF*, *ndhG*, *ndhH*, *ndhI*, *ndhJ* and *ndhK* genes. Black boxes denote intact genes, yellow boxes pseudogenized genes, red boxes fragmented and white boxes indicate deleted genes. Blue lines indicate the aquatic *Utricularia* species with complete *ndh* repertoire. Numbers of support values are all 100% for Bayesian inference, maximum likelihood and maximum parsimony bootstrap, except for outgroup clade *S*. *indicum* and *T*. *tetranolobum* with parsimony bootstrap value of 85%.

**Fig 5:**
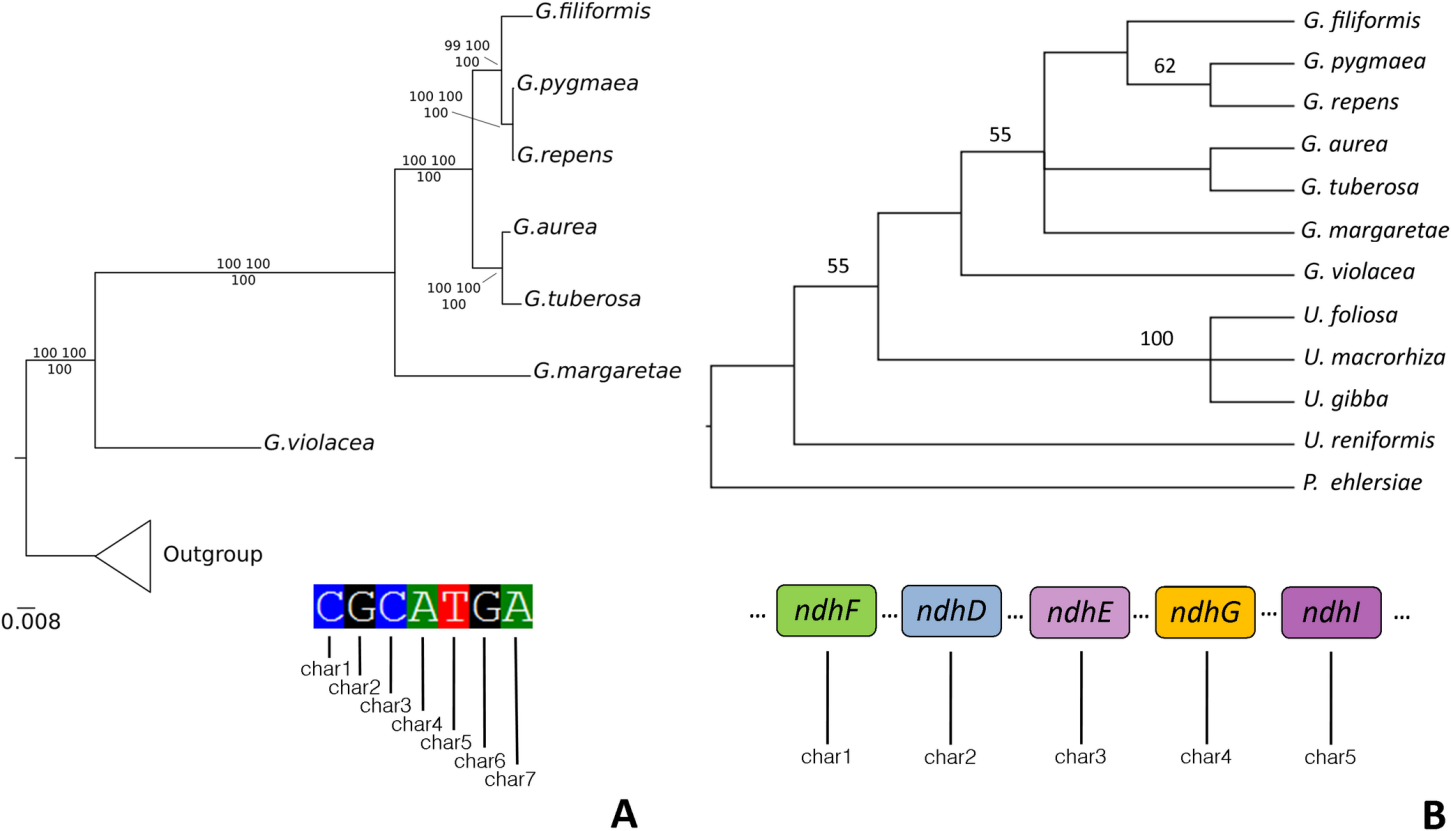
Phylogenetic hypothesis based on *ndh* sequences. **A.** Analyses based on *ndh* sites (nucleotide-by-nucleotide). In this analysis, each nucleotide was used as one character (e.g. char1, char2, char3) **B**. Strict consensus of the two most parsimonious trees (33 steps; IC = 0.70; IR = 0.78) based on the matrix codified for *ndh* patterns. In this analysis, each *ndh* gene was applied to two characters: one codified as absent (state 0) or present (state 1) (characters 1 to 11) and other codified as pseudogenized (state 0), decayed (state 1), complete (state 2) and inapplicable (state “-”, when the gene is deleted) (characters 12 to 22). For details see S2, S3 and S4 Tables.

Regarding the Lentibulariaceae, the topologies were totally congruent for all chloroplast dataset partitions (LSC, IR, SSC, coding regions, intergenic spacers and introns; S5 Fig). The whole chloroplast alignment resulted in 178,161 characters of which 21,687 are informative sites (Table 3). The most parsimony, Bayesian (BS) and maximum likelihood (ML) trees are highly congruent with very high support (ML bootstraps and posterior probabilities mostly 100) and support Lentibulariaceae as a monophyletic group, and *Genlisea*–*Utricularia* as sister clade with *Pinguicula*. When all branch lengths for each cladogram are visualized, the IR tree depicts very short branches (S5 Fig), resulting from the lowest proportion of variable sites (9%; Table 3). These results support that the *Genlisea* genus is monophyletic and its topology follows previous phylogenetic studies [18]: subgenus *Tayloria* (represented by *G*. *violacea*) as a sister clade to subgenus *Genlisea* (*G*. *margaretae*, *G*. *filiformis*, *G*. *pygmaea*, *G*. *repens*, *G*. *tuberosa* and *G*. *aurea*) (Fig 4). Moreover, the phylogenetic analyses based on the *ndh* genes partition, which treated each nucleotide ordinarily as a character, reveals a topology totally congruent to the trees resulting from other partitions and whole plastomes (Fig 4; S5 Fig). Also, when the processes that could be involved in the *ndh* degeneration (pseudogenization and decay) were codified in a multistate character matrix (see S2–S4 Tables); the resultant tree (Fig 5B) was mostly congruent with the nucleotide-by-nucleotide tree (Figs 4 and 5A).

**Table 3:** Datasets and phylogenetic statistics for each *Genlisea* cpDNA partition.

	Whole chloroplast	LSC	SSC	IR	Protein coding	Intergenic spacers	Introns	*ndh* genes
**Alignment (bp)**	178,161	99,235	20,156	28,636	67,437	46,068	15,090	9,462
**Overall GC content (%)–Only *Genlisea* species**	38.5	36.4	30.5	43.5	40.4	32.5	36.1	35.7
**Overall GC content (%)–*Genlisea* + outgroup**	38.1	36.1	31.3	43.1	40.4	32	35.9	35.2
**Variable sites (%)**	40,427 (22%)	27,508 (27%)	7,753 (38%)	2,752 (9%)	12,817 (19%)	15,502 (33%)	4,057 (26%)	1,944 (20%)
**Informative sites (%)**	21,687 (12%)	15,218 (15%)	4,275 (21%)	1,140 (4%)	6,909 (10%)	8,616 (18%)	2,360 (15%)	535 (6%)
**Consistency index (CI)**	0.856	0.852	0.837	0.922	0.845	0.855	0.847	0.976
**Retention index (RI)**	0.875	0.876	0.847	0.919	0.868	0.875	0.881	0.948
**Model of substitution (AICc)**	GTR+G+I	GTR+G+I	TVM+G+I	TVM+G+I	GTR+G+I	TVM+G+I	GTR+G+I	TVM+G

## Discussion

Chloroplast genomes are a powerful tool to understand the evolutionary forces acting on a species because their structure and sequence are highly constrained across flowering plants. The carnivorous plant family Lentibulariaceae has been shown to have a high rate of nucleotide substitution in all three genome compartments, including the chloroplast genome [10]. In this study we describe seven *Genlisea* cpDNA including both subgenera within carnivorous plant *Genlisea*: subgen. *Tayloria* (*G*. *violacea*) and subgen. *Genlisea* (*G*. *aurea*, *G*. *filiformis*, *G*. *pygmaea*, *G*. *repens*, *G*. *tuberosa* and *G*. *margaretae*).

The *Genlisea* cpDNA have typical quadripartite structure with a similar gene repertoire, as previously described for other Lentibulariaceae [25,49,50]. However, we do find that the *ndh* genes are deleted, fragmented or pseudogenized, which provides new insight into the evolutionary trajectory of *Genlisea* as well as the terrestrial forms of the Lentibulariaceae.

Even though cpDNAs are structurally conserved, changes in genome composition have been identified in many species of angiosperms [51] and also in some gnetophytes [52]. These variations are principally due to the expansion and contraction of IR and SSC regions [53] and gene loss and duplicated genes in IR/SC or IR/LSC boundaries [54]. Among the six cpDNAs described in this study and the previously published *Genlisea margaretae* cpDNA [50], *G*. *violacea* proved to be the most divergent from the other *Genlisea* species with possible IR expansion that includes duplication of *rps19* gene and partial duplication of *rpl22* gene. In addition, *G*. *filiformis* and *G*. *pygmaea* showed pseudogenization of *rps15* and *rpl22* genes, respectively. However, the absence of these genes is observed in other angiosperms. For instance, the *rpl22* gene was loss in several cpDNA, such as legumes [55,56], *Gossypium* [57], *Citrus* [58], *Castanea* [59], *Quercus* [60] and *Passiflora* species [61]. Moreover, some studies suggest that there is strong evidence that the *rpl22* gene has been transferred to the nucleus in some angiosperms [56,60].

The GC content among seed plant plastomes ranges between 34–40% and, comparing each cpDNA region, the SSC is the one with the lowest GC content [51]. For the *Genlisea* cpDNAs, we also found that the SSC had the lowest GC content (31.3%). One explanation for the SSC having the lowest GC content is that this region is susceptible to nucleotide substitutions, which is consistent with the high level of nucleotide variation (38%) we observed, compared to other cpDNA regions (Table 3).

The codon usage in *Genlisea* plastomes is similar to that reported for other Lentibulariaceae family cpDNA. Approximately 19,268 codons represent the coding repertoire of the protein coding regions (S7 Table). Codons frequency that ends with A and T have higher usage than G and C ending codons. For all plastomes the most frequent codon was Leucine (with approximately 1,989; 10.35%), whereas the least frequent was Cysteine (approximately 210–1.10%).

The identification of phylogenetically informative characters (PICs; including the parsimony informative characters) is an important procedure for evaluating characters with phylogenetic signal. Indeed, the PICs are represented by the synapomorphies [62,63] rather than nucleotide changes lacking phylogenetic signal. In this context, the results presented in this study support that the cpDNA is a powerful source of information for phylogenetic inferences. For *Genlisea*, two previous phylogenetic studies employed the cpDNA loci *trnK*/*matK* and *rps16* [4,18]. Our study suggests that other cpDNA regions (such as *ycf1*, *rpl20-rps12*, *rpoC2*) have more PICs and consequently have higher phylogenetic signal than previously considered sequences used to assess phylogenies and populations studies.

According to the Consortium for the Barcode of Life’s (CBOL), further studies are necessary to define the best DNA sequences for DNA barcoding of plants [64,65]. As many plants have poor resolution at the population level, previous studies have proposed using combinations of loci (as *matK*, *rbcL*, *trnH-psbA*), suggesting that no unique region exists [65,66]. However, a recent study suggested a single region in *ycf1* gene [67] could be used as a better barcode. Our PIC and divergence analysis corroborate usage of *ycf1* and/or *matK* for barcoding purposes, since *ycf1* is the first PIC classification and *matK* is the sixth (S6 Table).

Widely used in plant genotyping [68,69], SSRs are an important source of genetic variation that can be used for species discrimination, population structure and genetic diversity [69]. Similarly to our findings for *Genlisea* species, previous studies on cpDNA SSRs of Lentibulariaceae [70], reported that the chloroplast genomes have a large number of SSRs [25,50]; similarly, we find many SSRs across *Genlisea* species. Long repeats, represented by direct repeats and palindromic repeats can cause hairpin structures, which are associated with recombination, and can contribute significantly to rearranged gene order and addition of polymorphism [71,72]. In the evaluated *Genlisea* species, the long repeats were mainly found in non-coding regions, which is consistent with most angiosperms [73]. And, although long repeats are rare in Lentibulariaceae [50], both the smallest (*G*. *aurea*) and the largest chloroplast genomes (*G*. *violacea*) have a high number of direct repeats and palindromic repeats. In the *G*. *violacea* chloroplast genome, regions with palindromic repeats are found near the LSC/IR junctions, suggesting they could be contributing to IR expansion.

In *Utricularia reniformis* [25], repeat hotspots seem to be associated with *ndh* gene degradation, since some repeats regions are close to *ndh* genes. However, in *Genlisea* the repeats are dispersed over the cpDNA indicating that, for this genus, there is no relationship between the repeats and *ndh* pseudogenization. This observation suggests that, unlike *Utricularia*, different evolutionary processes are acting in the *Genlisea ndh* loci.

Different dataset partitions (IR, LSC, SSC, coding regions, intergenic spacers and introns; S5 Fig), recovered the same tree topology for Lentibulariaceae with high clade support. Indeed, all datasets contained a considerable percentage of informative characters, thus phylogenetic signal can be found along the whole *Genlisea* cpDNA.

The eleven *ndh* genes present in all *Genlisea* species are pseudogenized, decayed or even deleted (Fig 5; S4 Table). *ndh* genes losses have been found a few times in other taxa and are attributed to heterotrophic plants [23], some conifers [52], orchids [74], and other species of Lentibulariaceae [25,49,50]. And even with the remarkable degradation of *ndh* genes, the nucleotide composition of *ndh* still provides sufficient signal for a phylogenetic analysis (Figs 4 and 5). As such, the topology of *ndh* phylogenetic tree reveals the cladogenetic separation of different subgenera (*Tayloria* and *Genlisea*) and resolution of all *Genlisea* species (Fig 5). While in some orchids [74] the *ndh* losses seem to have no relation with taxonomy, and environment where these species are found, in Lentibulariaceae the *ndh* genes appear to have been maintained in aquatic taxa [25,49,50].

When the *ndh* gene events (arisen, pseudogenization, decay or deletion) are traced in the total evidence (entire plastomes) phylogenetic analysis (Fig 4), we can verify a different scenario when comparing *Genlisea* lineages to *Pinguicula* and *Utricularia* lineages. The terrestrial taxa *Pinguicula* (represented by the *P*. *ehlersiae*) and *U*. *reniformis* have most *ndh* genes as pseudogenized or deleted. Interestingly, the clade represented by the aquatic species of *Utricularia* (*U*. *foliosa*, *U*. *macrorhiza*, and *U*. *gibba*) has gained, probably as independent (or not) reversion events (Fig 4; clade denoted with blue lines), the almost entire *ndh* repertoire. We have previously shown that aquatic species of *Utricularia* have maintained and conserved *ndh* genes [25]. The *ndh* genes activity appears dispensable under favorable conditions, as pointed out by transcriptomic studies [75] and verified in knock-out mutants [76–78]. But, episodes of abiotic stress can impact terrestrial habitats and, according to Ruhlman *et al*. [75], appear to be the cause of retention of *ndh* genes. Nonetheless, our phylogenetic hypothesis shows that Lentibulariaceae follows an opposite trend, since terrestrial species of *Pinguicula*, *U*. *reniformis* and all seven *Genlisea* possess degenerated *ndh* genes and the aquatic species of *Utricularia*, on the other hand, display a conserved *ndh* repertoire. Moreover, it is important to emphasize that the aquatic environment also provides a stressful habitat for plants, since these habitats can present low carbon and light availability, anoxia, wave exposure, significant restrictions to sexual reproduction, and sometimes also osmotic stress and limited nutrient supply [79]. Thus, the complete recovery of all eleven genes for the aquatic *Utricularia* supports the hypothesis that the *ndh* genes are conserved in stressful habitats.

The trend of decay and deletion of the *ndh* genes, represented within the different lineages of *Genlisea* is remarkable. The *Genlisea* clade presented the highest concentration of fragmented and deleted *ndh* genes, when compared to *Utricularia* and *Pinguicula* species (Fig 4). In an attempt to phylogenetically test this tendency of *ndh* genes to degrade in *Genlisea* lineages, we codified the state (present, pseudogenized, decayed or deleted; S2–S4 Tables) for each of eleven plastidial *ndh* genes and carried out a parsimony analysis. The consensual topology of both most parsimonious trees (Fig 5A and 5B) also supports this hypothesis when compared with the total evidence tree (Fig 4).

As in most Lentibulariaceae cpDNA, the loss of *ndh* genes does not seem to affect plant fitness despite the harsh environmental conditions common for the carnivorous habit [25,50]. However, as seen in the present study, it has been reported that in terrestrial species most of *ndh* genes were lost for Lentibulariaceae species (Fig 1).

Silva *et al*. [80] identified several pseudogenes of plastid origin in *U*. *reniformis* mtDNA. For instance, the presence of the *ndhJ-ndhK-ndhC* loci in the mtDNA supports the hypothesis of lateral transfer since these genes are absent in the cpDNA [80]. Similar translocation of *ndh* genes from the plastome to the mitochondrial genome was also suggested to the Epidendroideae orchid *Erycina pusilla* [81]. According to this study, other than the *ndh* genes could be transferred to mtDNA, since more than 76% of the cpDNA genome was transferred into the mtDNA genome of *E*. *pusilla* and the largest cpDNA insertion into the mtDNA genome in this species was 12kb.

In addition to the transfer of plastid genes to the mtDNA, cpDNA genes can also be transferred to the nuclear genome [82]. With the *G*. *aurea* nuclear genome published [26], we performed blastn and tblastn searches of all plastidial *ndh* genes subunits and none of these genes were also found in nuclear assembled scaffolds. However, one cannot discard the idea that these genes are present in the mtDNA. This hypothesis has to be further investigated since the mitochondrial genome is not available [26].

Studies have pointed out the function of *ndh* genes for modulating ROS in chloroplasts [23]. Plants with high expression of *ndh* genes also have an increasing concentration of ROS, which can lead to the cell death [83]. Assuming that terrestrial environments are less stressful than aquatic ones [79], the presence of complete *ndh* repertoire is understandable for aquatic species of Lentibulariaceae, since these genes are important for ROS modulation in the presence of their high respiratory rates. However, only the aquatic Lentibulariaceae species of *Utricularia* have had their respiration rates measured [84]. More chloroplast genomes from the *Genlisea* and *Utricularia* lineages are required to test this hypothesis. But the oxidant activities of ROS are well known for DNA [14,85,16] and it is not difficult to suppose their deleterious action even in genomes from different compartments.

## Conclusions

Here we report the chloroplast genome of six *Genlisea* species of both subgenera: *Tayloria* and *Genlisea*. These genomes were compared with the previously published *G*. *margaretae* cpDNA, showing that they are very similar in content and have the same gene order and quadripartite structure. Phylogenomic analysis showed that using coding regions, non-coding regions and even decayed *ndh* sequences it is possible to obtain the evolutionary history with great congruence, recovering with high support the position of assessed taxa in *Genlisea* genus and Lentibulariaceae family. Importantly, we corroborate previous observations that distinct from the aquatic taxa of Lentibulariaceae, the terrestrial *Genlisea* chloroplast genomes showed a pseudogenization and a progressive degradation of *ndh* genes, as reported for other Lentibulariaceae. In summary, we propose that the Lentibulariaceae system provides an important opportunity to understand the evolutionary forces that govern the transition to an aquatic environment, and may provide insight into how plants manage water stress at a genome scale. These findings may have implications for engineering crop species for better water stress tolerance, both too much and too little water.

## Supporting information

S1 Fig Coverage and read identity plots for the reconstructed plastid genomes of *Genlisea* species. All quality-trimmed reads from sequencing data sets have been mapped back to the reconstructed plastid supercontig. The upper plot indicates the identity per site and the lower plot shows the coverage plot per species.
(DOCX)

S2 Fig Agarose gel electrophoresis (0.8%) of PCR products of the cpDNA IR/LSC boundary of *Genlisea violacea* (3 bioreplicates = 3 specimens), *G*. *aurea*, *G*. *filiformis*, and *G*. *tuberosa*. Note the product of *G*. *violacea* cpDNA that presents the duplication of *rps19* gene and *rpl22* as pseudogene (amplicon with 1,194 bp), while the other species present an expected product with ~490 bp. (Amplification reactions of the rpl2-trnH(GUG) marker were conducted in 25 μL of the solution containing 20 mM of MgCl2, 100 mM of dNTPs, 10 mM of each primer, 1 U of Dream Taq Polymerase–Fermentas, and 50 ng of DNA template. The thermal profile for amplification was 1min at 94°C; 35 cycles of 40s at 94°C, 20s at 64°C, 90s at 72°C, and 5min of final extension at 72°C. Forward primer = 5’-AGT CGG ACA AGT GGG GAA TG-3’; reverse primer = 5’-GGA TGT GGC CAA GTG GAT CA-3’).
(DOCX)

S3 Fig Correlation between p-distance and phylogenetically informative characters (PICs). Statistics from Spearman correlation tests are given near the corresponding trend lines.
(DOCX)

S4 Fig Phylogenetically informative characters (PIC) and p-distance in *Genlisea* cpDNA based on alignment data. PIC values are represented as bars and cpDNA region is marked by colors. Black dots represent p-distance. Only PIC of *ndhs* were not calculated to avoid p-distance alignment artefact (see S6 Table).
(DOCX)

S5 Fig Phylogenomic trees based on different datasets for *Genlisea* species. Numbers above are parsimony bootstrap (left), maximum likelihood bootstrap (right) and posterior probability values are represented below. Lamiales species were used as outgroup.
(DOCX)

S1 Table Summary of sequencing data for *Genlisea* species. (DOCX)

S2 Table Characters and states codified of *ndh* genes for Lentibulariaceae. (DOCX)

S3 Table Matrix with codified characters of *ndh* genes for Lentibulariaceae. The characters were codified according the S2 Table. (G. = *Genlisea*; P. = *Pinguicula*; U. = *Utricularia*).
(DOCX)

S4 Table *ndh* genes length variation among *Genlisea* and *Utricularia gibba* species. Numbers within table refer to sequence length (bp). Colors refer to the state of character: white–deleted gene; yellow–pseudogenized; pink–decayed gene; grey–complete gene; n/a–absent.
(DOCX)

S5 Table Repeats (direct, palindromic and tandem) for each *Genlisea* species. F–Direct repeats; P–Palindromic repeats; T–Tandem repeats (inside parenthesis the repeated nucleotide). Common genes with repeats between the six species are highlighted with yellow background color in *G*. *aurea* table.
(DOCX)

S6 Table Phylogenetically informative characters (PIC) and p-distance of each gene for *Genlisea* species. Deleted *ndh* genes in all *Genlisea* species and boundaries between *ndh* pseudogenes are uncertain and were not included in this analysis (represented as n/a).
(DOCX)

S7 Table Codon usage and amino acid frequencies for *Genlisea* plastomes. (DOCX)
