# Supplementary material for: Comparative genomic analysis of Genlisea (corkscrew plants—Lentibulariaceae) chloroplast genomes reveals an increasing loss of the ndh genes
Source: PLoS One. 2018 Jan 2;13(1):e0190321. doi: 10.1371/journal.pone.0190321 (PMC5749785; doi:10.1371/journal.pone.0190321)
Supplement: S1 Fig — All quality-trimmed reads from sequencing data sets have been mapped back to the reconstructed plastid supercontig. The upper plot indicates the identity per site and the lower plot shows the coverage plot per species. (DOCX) [file pone.0190321.s001.docx]

**
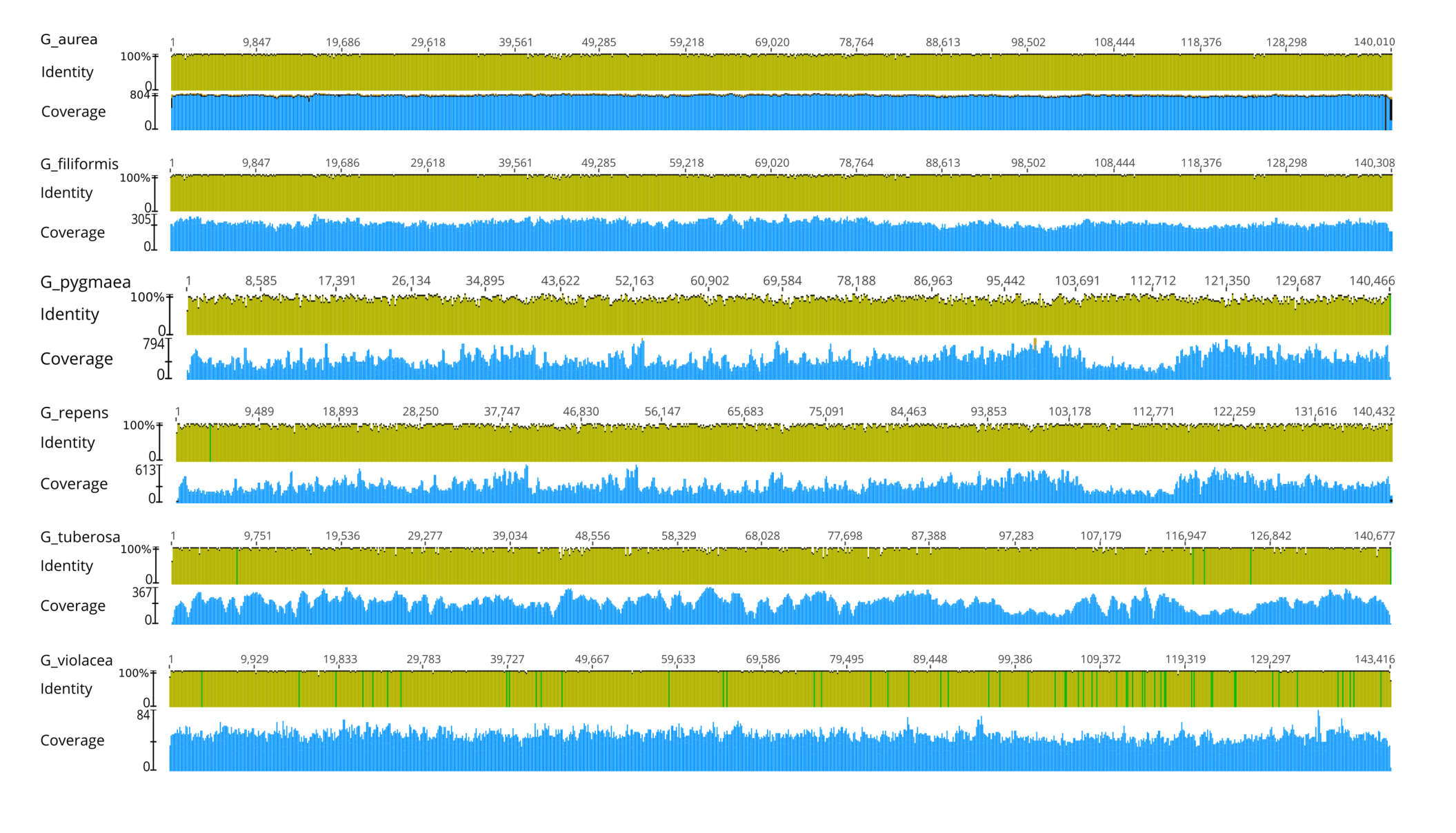
S1 Fig. Coverage and read identity plots for the reconstructed plastid genomes of *Genlisea* species**. All quality-trimmed reads from sequencing data sets have been mapped back to the reconstructed plastid supercontig. The upper plot indicates the identity per site and the lower plot shows the coverage plot per species.
