# Supplementary material for: Comparative genomic analysis of Genlisea (corkscrew plants—Lentibulariaceae) chloroplast genomes reveals an increasing loss of the ndh genes
Source: PLoS One. 2018 Jan 2;13(1):e0190321. doi: 10.1371/journal.pone.0190321 (PMC5749785; doi:10.1371/journal.pone.0190321)
Supplement: S2 Fig — Note the product of G. violacea cpDNA that presents the duplication of rps19 gene and rpl22 as pseudogene (amplicon with 1,194 bp), while the other species present an expected product with ~490 bp. (Amplification reactions of the rpl2-trnH(GUG) marker were conducted in 25 μL of the solution containing 20 mM of MgCl2, 100 mM of dNTPs, 10 mM of each primer, 1 U of Dream Taq Polymerase–Fermentas, and 50 ng of DNA template. The thermal profile for amplification was 1min at 94°C; 35 cycles of 40s at 94°C, 20s at 64°C, 90s at 72°C, and 5min of final extension at 72°C. Forward primer = 5’-AGT CGG ACA AGT GGG GAA TG-3’; reverse primer = 5’-GGA TGT GGC CAA GTG GAT CA-3’). (DOCX) [file pone.0190321.s002.docx]

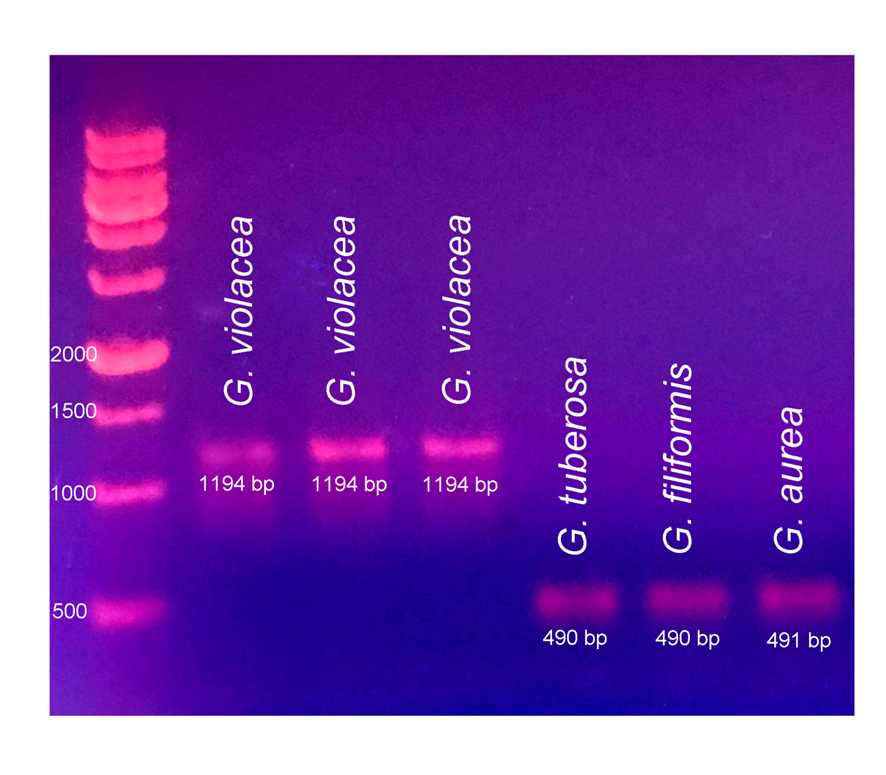


**S2 Fig. Agarose gel electrophoresis (0.8%) of PCR products of the cpDNA IR/LSC boundary of *Genlisea violacea* (3 bioreplicates = 3 specimens), *G. aurea*, *G. filiformis*, and *G. tuberosa*.** Note the product of *G. violacea* cpDNA that presents the duplication of *rps19* gene and *rpl22* as pseudogene (amplicon with 1,194 bp), while the other species present an expected product with ~490 bp. (Amplification reactions of the rpl2-trnH(GUG) marker were conducted in 25 μL of the solution containing 20 mM of MgCl2, 100 mM of dNTPs, 10 mM of each primer, 1 U of Dream Taq Polymerase – Fermentas, and 50 ng of DNA template. The thermal profile for amplification was 1min at 94°C; 35 cycles of 40s at 94°C, 20s at 64°C, 90s at 72°C, and 5min of final extension at 72°C. Forward primer = 5’-AGT CGG ACA AGT GGG GAA TG-3’; reverse primer = 5’-GGA TGT GGC CAA GTG GAT CA-3’).
