# Supplementary material for: Comparative genomic analysis of Genlisea (corkscrew plants—Lentibulariaceae) chloroplast genomes reveals an increasing loss of the ndh genes
Source: PLoS One. 2018 Jan 2;13(1):e0190321. doi: 10.1371/journal.pone.0190321 (PMC5749785; doi:10.1371/journal.pone.0190321)
Supplement: S3 Fig — Statistics from Spearman correlation tests are given near the corresponding trend lines. (DOCX) [file pone.0190321.s003.docx]

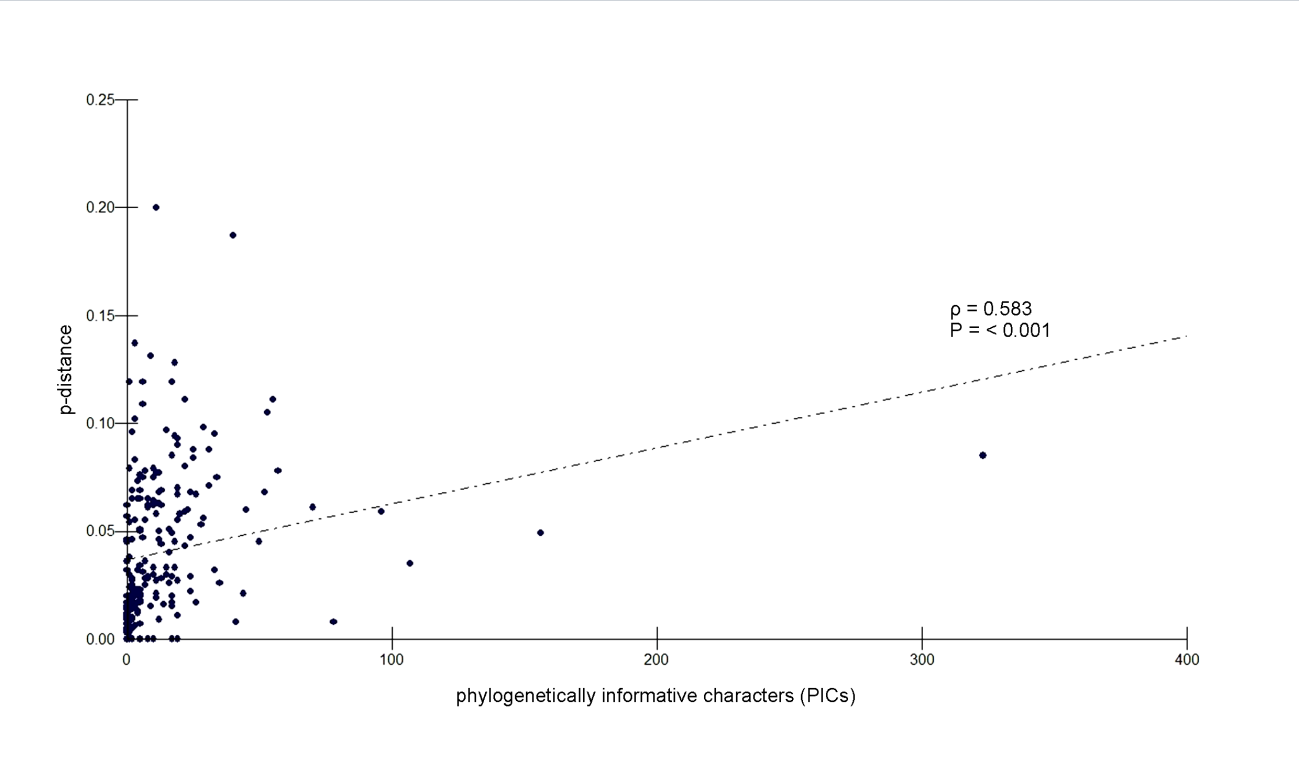


**S3 Fig. Correlation between p-distance and phylogenetically informative characters (PICs).** Statistics from Spearman correlation tests are given near the corresponding trend lines.
