# Supplementary material for: Comparative genomic analysis of Genlisea (corkscrew plants—Lentibulariaceae) chloroplast genomes reveals an increasing loss of the ndh genes
Source: PLoS One. 2018 Jan 2;13(1):e0190321. doi: 10.1371/journal.pone.0190321 (PMC5749785; doi:10.1371/journal.pone.0190321)
Supplement: S4 Fig — PIC values are represented as bars and cpDNA region is marked by colors. Black dots represent p-distance. Only PIC of ndhs were not calculated to avoid p-distance alignment artefact (see S6 Table). (DOCX) [file pone.0190321.s004.docx]

**
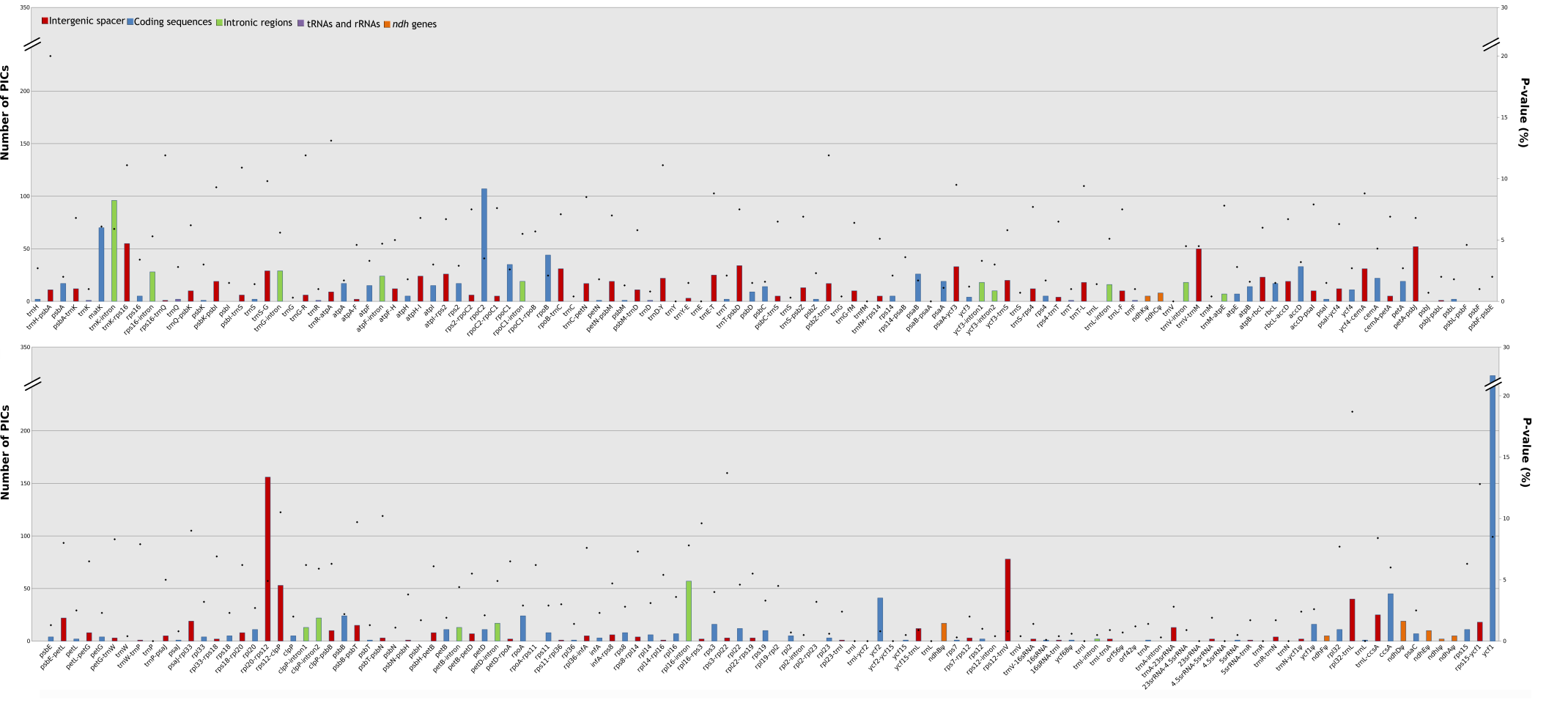
**

**S4 Fig. Phylogenetically informative characters (PIC) and p-distance in *Genlisea* cpDNA based on alignment data.** PIC values are represented as bars and cpDNA region is marked by colors. Black dots represent p-distance. Only PIC of *ndhs* were not calculated to avoid p-distance alignment artifact (see S6 Table).
