# Supplementary material for: Comparative genomic analysis of Genlisea (corkscrew plants—Lentibulariaceae) chloroplast genomes reveals an increasing loss of the ndh genes
Source: PLoS One. 2018 Jan 2;13(1):e0190321. doi: 10.1371/journal.pone.0190321 (PMC5749785; doi:10.1371/journal.pone.0190321)
Supplement: S5 Fig — Numbers above are parsimony bootstrap (left), maximum likelihood bootstrap (right) and posterior probability values are represented below. Lamiales species were used as outgroup. (DOCX) [file pone.0190321.s005.docx]

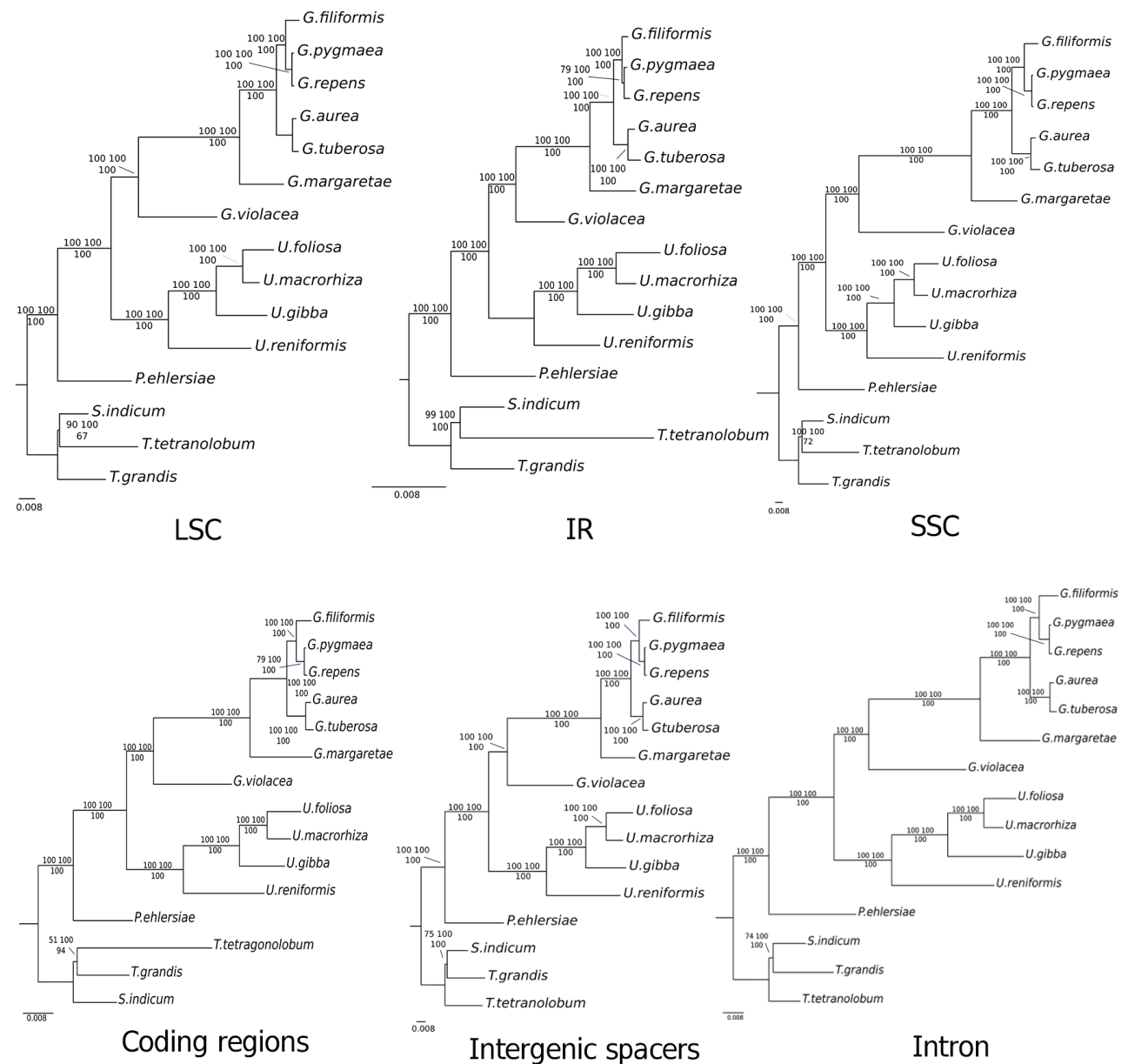


**S5 Fig. Phylogenomic trees based on different datasets for *Genlisea* species.** Numbers above are parsimony bootstrap (left), maximum likelihood bootstrap (right) and posterior probability values are represented below. Lamiales species were used as outgroup.
