# Supplementary material for: Comparative genomic analysis of Genlisea (corkscrew plants—Lentibulariaceae) chloroplast genomes reveals an increasing loss of the ndh genes
Source: PLoS One. 2018 Jan 2;13(1):e0190321. doi: 10.1371/journal.pone.0190321 (PMC5749785; doi:10.1371/journal.pone.0190321)
Supplement: S1 Table — (DOCX) [file pone.0190321.s006.docx]

**S1 Table. Summary of sequencing data for *Genlisea* species.**

| Species | Source^1^ | Voucher^2^ | Paired ends size for each data (bp) | Number of raw reads | Total read length (bp) | Number of high quality reads | Mapped reads (bp) | Contigs (n) | Coverage (×)^3^ |
| --- | --- | --- | --- | --- | --- | --- | --- | --- | --- |
| *Genlisea aurea* | Cultivated | - | 2 × 100 | 153,411,688 | 15,494,580,488 | 130,921,354 | 5,760,995 | 04 | 780.2 |
| *G. filiformis* | Natural pop. | VFOM1962 | 2 × 80 | 1,160,600 | 193,868,596 | 1,014,417 | 141,557 | 04 | 205.5 |
| *G. pygmaea* | Cultivated | VFOM1970 | 2 × 150 | 2,888,131 | 872,215,562 | 2,576,638 | 224,370 | 03 | 339.3 |
| *G. repens* | Cultivated | VFOM1965 | 2 × 150 | 2,767,896 | 835,904,592 | 2,430,284 | 166,706 | 03 | 246.5 |
| *G. tuberosa* | Natural pop. | VFOM2001 | 2 × 300 | 2,758,854 | 1,643,240,506 | 2,203,835 | 142,268 | 02 | 220.3 |
| *G. violacea* | Natural pop. | VFOM1963 | 2 × 80 | 1,449,666 | 241,781,382 | 1,354,160 | 30,777 | 03 | 40.2 |

^1^ Collected from natural population.

^2^ Deposited in Herbarium JABU (University of Sao Paulo State, Unesp/ FCAV, Brazil).

^3^ Coverage based on the mean of mapped reads using Bowtie2 (parameters: --very-sensitive; --end-to-end).
