# Supplementary material for: Comparative genomic analysis of Genlisea (corkscrew plants—Lentibulariaceae) chloroplast genomes reveals an increasing loss of the ndh genes
Source: PLoS One. 2018 Jan 2;13(1):e0190321. doi: 10.1371/journal.pone.0190321 (PMC5749785; doi:10.1371/journal.pone.0190321)
Supplement: S2 Table — (DOCX) [file pone.0190321.s007.docx]

**S2 Table. Characters and states codified of *ndh* genes for Lentibulariaceae.**

| N Character States |
| --- |

1 ndhA (0) absent (1) present

2 ndhB (0) absent (1) present

3 ndhC (0) absent (1) present

4 ndhD (0) absent (1) present

5 ndhE (0) absent (1) present

6 ndhF (0) absent (1) present

7 ndhG (0) absent (1) present

8 ndhH (0) absent (1) present

9 ndhI (0) absent (1) present

10 ndhJ (0) absent (1) present

11 ndhK (0) absent (1) present

12 ndhA – status (0) pseudogenized (1) decayed (2) complete (-) inapplicable

13 ndhB – status (0) pseudogenized (1) decayed (2) complete (-) inapplicable

14 ndhC – status (0) pseudogenized (1) decayed (2) complete (-) inapplicable

15 ndhD – status (0) pseudogenized (1) decayed (2) complete (-) inapplicable

16 ndhE – status (0) pseudogenized (1) decayed (2) complete (-) inapplicable

17 ndhF – status (0) pseudogenized (1) decayed (2) complete (-) inapplicable

18 ndhG – status (0) pseudogenized (1) decayed (2) complete (-) inapplicable

19 ndhH – status (0) pseudogenized (1) decayed (2) complete (-) inapplicable

20 ndhI – status (0) pseudogenized (1) decayed (2) complete (-) inapplicable

21 ndhJ – status (0) pseudogenized (1) decayed (2) complete (-) inapplicable

22 ndhK – status (0) pseudogenized (1) decayed (2) complete (-) inapplicable
