# Supplementary material for: Comparative genomic analysis of Genlisea (corkscrew plants—Lentibulariaceae) chloroplast genomes reveals an increasing loss of the ndh genes
Source: PLoS One. 2018 Jan 2;13(1):e0190321. doi: 10.1371/journal.pone.0190321 (PMC5749785; doi:10.1371/journal.pone.0190321)
Supplement: S3 Table — The characters were codified according the S2 Table. (G. = Genlisea; P. = Pinguicula; U. = Utricularia). (DOCX) [file pone.0190321.s008.docx]

**S3 Table. Matrix with codified characters of *ndh* genes for Lentibulariaceae.** The characters were codified according the S2 Table. (*G.*= *Genlisea*; *P.*= *Pinguicula*; *U.*= *Utricularia*)

| Taxa/ 1111111111222 |
| --- |
| Characters 1234567890123456789012 |

*G. filiformis* 1111100010110010---0-0

*G. pygmaea* 1111100010110010---1-0

*G. repens* 1111100010110010---1-0

*G. tuberosa* 11111100101100111--0-0

*G. aurea* 01111100001-00111----0

*G. margaretae* 1111110010010-101--0--

*G. violacea* 01111110010-000011--0-

*U. foliosa* 1111111111122222222222

*U. macrorhiza* 1111111111122222222222

*U. gibba* 1111111111122222222222

*U. reniformis* 1101101110000-00-000--

*P. ehlersiae* 1101101111100-00-00000
