# Supplementary material for: Comparative genomic analysis of Genlisea (corkscrew plants—Lentibulariaceae) chloroplast genomes reveals an increasing loss of the ndh genes
Source: PLoS One. 2018 Jan 2;13(1):e0190321. doi: 10.1371/journal.pone.0190321 (PMC5749785; doi:10.1371/journal.pone.0190321)
Supplement: S4 Table — Numbers within table refer to sequence length (bp). Colors refer to the state of character: white–deleted gene; yellow–pseudogenized; pink–decayed gene; grey–complete gene; n/a–absent. (DOCX) [file pone.0190321.s009.docx]

| **S4 Table.** ***ndh* genes length variation among *Genlisea* and *Utricularia* *gibba* species.** Numbers within table refer to sequence length (bp). Colors refer to the state of character: white – deleted gene; yellow – pseudogenized; pink – decayed gene; grey – complete gene; n/a – absent. | | | | | | | | | | | |
| --- | --- | --- | --- | --- | --- | --- | --- | --- | --- | --- | --- |
| Species/*ndh* | A | B | C | D | E | F | G | H | I | J | K |
| *G. tuberosa* | 173 | 1,474 | 318 | 258 | 82 | 59 | n/a | n/a | 189 | n/a | 406 |
| *G. aurea* | n/a | 1,474 | 318 | 270 | 88 | 42 | n/a | n/a | n/a | n/a | 419 |
| *G. repens* | 166 | 1,467 | 213 | 294 | 124 | n/a | n/a | n/a | 79 | n/a | 427 |
| *G. pygmaea* | 166 | 1,467 | 213 | 294 | 124 | n/a | n/a | n/a | 79 | n/a | 427 |
| *G. filiformis* | 160 | 1,474 | 316 | 292 | 124 | n/a | n/a | n/a | 199 | n/a | 417 |
| *G. margaretae* | 189 | 1,474 | n/a | 321 | 238 | 25 | n/a | n/a | 469 | n/a | n/a |
| *G. violacea* | n/a | 1,505 | 183 | 1,338 | 295 | 311 | 92 | n/a | n/a | 332 | n/a |
| *U. gibba* | 1,092 | 1,533 | 363 | 1,530 | 306 | 2,265 | 531 | 1,181 | 528 | 477 | 678 |
