# Supplementary material for: Comparative genomic analysis of Genlisea (corkscrew plants—Lentibulariaceae) chloroplast genomes reveals an increasing loss of the ndh genes
Source: PLoS One. 2018 Jan 2;13(1):e0190321. doi: 10.1371/journal.pone.0190321 (PMC5749785; doi:10.1371/journal.pone.0190321)
Supplement: S5 Table — F–Direct repeats; P–Palindromic repeats; T–Tandem repeats (inside parenthesis the repeated nucleotide). Common genes with repeats between the six species are highlighted with yellow background color in G. aurea table. (DOCX) [file pone.0190321.s010.docx]

**S5 Table. Repeats (direct, palindromic and tandem) for each *Genlisea* species**. F – Direct repeats; P – Palindromic repeats; T – Tandem repeats (inside parenthesis the repeated nucleotide). Common genes with repeats between the six species are highlighted with yellow background color in *G. aurea* table.

| **Sequence repeats in *Genlisea aurea.* Type, length, region, location and plastome quadripartite region. (F = Forward repeat; P= Palindromic repeat; T = Tandem repeat)** | | | | | | |
| --- | --- | --- | --- | --- | --- | --- |
| Repeat Start 1 | Type | Lenght (bp) | Repeat Start 2 | Gene | Location | Region |
| 68226 | F | 34 | 68260 | clpP | intron | LSC |
| 38023 | F | 40 | 40253 | psaB;psaA | CDS | LSC |
| 49479 | F | 37 | 49481 | trnM(CAU)-atpE | IGS | LSC |
| 49479 | F | 39 | 49483 | trnM(CAU)-atpE | IGS | LSC |
| 49482 | F | 39 | 49487 | trnM(CAU)-atpE | IGS | LSC |
| 49487 | F | 30 | 49488 | trnM(CAU)-atpE | IGS | LSC |
| 49479 | F | 36 | 49482 | trnM(CAU)-atpE | IGS | LSC |
| 49475 | F | 38 | 49484 | trnM(CAU)-atpE | IGS | LSC |
| 49479 | F | 35 | 49487 | trnM(CAU)-atpE | IGS | LSC |
| 49480 | F | 35 | 49487 | trnM(CAU)-atpE | IGS | LSC |
| 49481 | F | 35 | 49487 | trnM(CAU)-atpE | IGS | LSC |
| 27099 | F | 33 | 27122 | rpoB-trnC(GCA) | IGS | LSC |
| 49479 | F | 33 | 49489 | trnM(CAU)-atpE | IGS | LSC |
| 60858 | F | 33 | 60891 | petA-psbJ | IGS | LSC |
| 49479 | F | 32 | 49490 | trnM(CAU)-atpE | IGS | LSC |
| 49479 | F | 31 | 49491 | trnM(CAU)-atpE | IGS | LSC |
| 49479 | F | 30 | 49492 | trnM(CAU)-atpE | IGS | LSC |
| 49479 | F | 31 | 66659 | trnM(CAU)-atpE; rps12-clpP | IGS | LSC |
| 49487 | F | 31 | 49495 | trnM(CAU)-atpE | IGS | LSC |
| 49493 | F | 31 | 78919 | trnM(CAU)-atpE; rps16 | IGS;intron | LSC |
| 7748 | F | 30 | 14281 | psbK-psbI; atpH-atpI | IGS | LSC |
| 8022 | F | 30 | 34873 | psbI-trnS(GCU); psbC-trnS(UGA) | IGS | LSC |
| 9592 | F | 30 | 35802 | trnG(UCC);trnG(UCC) | tRNA | LSC |
| 49479 | F | 30 | 49493 | trnM(CAU)-atpE | IGS | LSC |
| 49481 | F | 30 | 66665 | trnM(CAU)-atpE; rps12-clpP | IGS | LSC |
| 49487 | F | 30 | 49496 | trnM(CAU)-atpE | IGS | LSC |
| 68281 | F | 30 | 68307 | clpP | intron | LSC |
| 8506 | P | 42 | 8548 | trnS(GUC)-trnG(UCC) | IGS | LSC |
| 56865 | P | 36 | 56901 | psaI-ycf4 | IGS | LSC |
| 8022 | P | 30 | 44716 | psbI-trnS(GCU);trnS(GGA) | IGS;tRNA | LSC |
| 63914 | P | 32 | 63953 | psaJ-rpl33 | IGS | LSC |
| 34873 | P | 30 | 44716 | psbC-trnS(UGA); trnS(GGA) | IGS;tRNA | LSC |
| 2234 | T(T) | 10 | 2243 | matK | CDS | LSC |
| 2933 | T(T) | 8 | 2940 | matK | CDS | LSC |
| 7353 | T(T) | 8 | 7360 | psbK | CDS | LSC |
| 8161 | T(T) | 8 | 8168 | trnS(GUC)-trnG(UCC) | IGS | LSC |
| 8319 | T(T) | 9 | 8327 | trnS(GUC)-trnG(UCC) | IGS | LSC |
| 8732 | T(A) | 7 | 8738 | trnS(GUC)-trnG(UCC) | IGS | LSC |
| 9174 | T(A) | 10 | 9183 | trnG(UCC) | intron | LSC |
| 12664 | T(A) | 8 | 12671 | atpF | CDS | LSC |
| 13147 | T(A) | 8 | 13154 | atpF-atpH | IGS | LSC |
| 15227 | T(A) | 8 | 15234 | atpI-rps2 | IGS | LSC |
| 16349 | T(T) | 8 | 16356 | rpoC2 | CDS | LSC |
| 18075 | T(T) | 7 | 18081 | rpoC2 | CDS | LSC |
| 18343 | T(A) | 9 | 18351 | rpoC2 | CDS | LSC |
| 20628 | T(T) | 7 | 20634 | rpoC1 | CDS | LSC |
| 20819 | T(A) | 8 | 20826 | rpoC1 | CDS | LSC |
| 22271 | T(A) | 10 | 22280 | rpoC1 | intron | LSC |
| 22470 | T(A) | 13 | 22482 | rpoC1 | intron | LSC |
| 22676 | T(A) | 7 | 22682 | rpoC1 | intron | LSC |
| 25473 | T(A) | 7 | 25479 | rpoB | CDS | LSC |
| 27076 | T(A) | 7 | 27082 | rpoB-trnC(GCA) | IGS | LSC |
| 27385 | T(T) | 16 | 27400 | rpoB-trnC(GCA) | IGS | LSC |
| 27594 | T(T) | 7 | 27600 | rpoB-trnC(GCA) | IGS | LSC |
| 27954 | T(A) | 10 | 27963 | trnC(GCA)-petN | IGS | LSC |
| 28277 | T(T) | 9 | 28285 | trnC(GCA)-petN | IGS | LSC |
| 29648 | T(T) | 7 | 29654 | psbM-trnD(GUC) | IGS | LSC |
| 29775 | T(T) | 11 | 29785 | psbM-trnD(GUC) | IGS | LSC |
| 31976 | T(T) | 7 | 31982 | trnT(GGU)-psbD | IGS | LSC |
| 33548 | T(G) | 7 | 33554 | psbC | CDS | LSC |
| 34298 | T(G) | 7 | 34304 | psbC | CDS | LSC |
| 34765 | T(T) | 7 | 34771 | psbC-trnS(UGA) | IGS | LSC |
| 35984 | T(A) | 7 | 35990 | trnG(UCC)-trnfM(CAU) | IGS | LSC |
| 39138 | T(A) | 7 | 39144 | psaA | CDS | LSC |
| 39624 | T(C) | 7 | 39630 | psaA | CDS | LSC |
| 40364 | T(A) | 7 | 40370 | psaA | CDS | LSC |
| 41366 | T(T) | 10 | 41375 | psaA-ycf3 | IGS | LSC |
| 42474 | T(T) | 8 | 42481 | ycf3 | intron | LSC |
| 42814 | T(T) | 11 | 42824 | ycf3 | intron | LSC |
| 42943 | T(T) | 9 | 42951 | ycf3 | intron | LSC |
| 46428 | T(A) | 14 | 46441 | trnL(UAA) | intron | LSC |
| 47076 | T(T) | 9 | 47084 | trnL(UAA)-trnF(GAA) | IGS | LSC |
| 47593 | T(T) | 7 | 47599 | ndhK(pseudo) | pseudo | LSC |
| 48263 | T(A) | 9 | 48271 | ndhC(pseudo)-trnV(UAC) | IGS | LSC |
| 51934 | T(T) | 12 | 51945 | atpB-rbcL | IGS | LSC |
| 53910 | T(T) | 7 | 53916 | rbcL-accD | IGS | LSC |
| 54201 | T(A) | 9 | 54209 | rbcL-accD | IGS | LSC |
| 56382 | T(A) | 7 | 56388 | accD-psaI | IGS | LSC |
| 57797 | T(T) | 13 | 57809 | ycf4-cemA | IGS | LSC |
| 59506 | T(A) | 7 | 59512 | petA | CDS | LSC |
| 59977 | T(T) | 7 | 59983 | petA | CDS | LSC |
| 60106 | T(A) | 7 | 60112 | petA-psbJ | IGS | LSC |
| 60241 | T(T) | 7 | 60247 | petA-psbJ | IGS | LSC |
| 60527 | T(T) | 15 | 60541 | petA-psbJ | IGS | LSC |
| 60841 | T(A) | 11 | 60851 | petA-psbJ | IGS | LSC |
| 61431 | T(A) | 8 | 61438 | psbF | CDS | LSC |
| 62315 | T(A) | 7 | 62321 | psbE-petL | IGS | LSC |
| 62568 | T(A) | 8 | 62575 | psbE-petL | IGS | LSC |
| 63018 | T(T) | 7 | 63024 | petG-trnW(CCA) | IGS | LSC |
| 63217 | T(A) | 7 | 63223 | trnW(CCA)-trnP(UGG) | IGS | LSC |
| 63817 | T(T) | 7 | 63823 | psaJ | CDS | LSC |
| 63939 | T(T) | 7 | 63945 | psAJ-rpl33 | IGS | LSC |
| 66205 | T(T) | 7 | 66211 | rpl20-rps12 | IGS | LSC |
| 67572 | T(A) | 11 | 67582 | clpP | intron | LSC |
| 67951 | T(A) | 7 | 67957 | clpP | CDS | LSC |
| 68065 | T(A) | 7 | 68071 | clpP | intron | LSC |
| 68474 | T(T) | 9 | 68482 | clpP | intron | LSC |
| 68676 | T(T) | 7 | 68682 | clpP | intron | LSC |
| 69984 | T(T) | 8 | 69991 | psbB | CDS | LSC |
| 70333 | T(T) | 7 | 70339 | psbB | CDS | LSC |
| 71912 | T(A) | 7 | 71918 | psbH-petB | IGS | LSC |
| 72110 | T(A) | 12 | 72121 | petB | intron | LSC |
| 73496 | T(T) | 9 | 73504 | petD | intron | LSC |
| 75700 | T(T) | 7 | 75706 | rps11 | CDS | LSC |
| 76164 | T(T) | 7 | 76170 | rps11-rpl36 | IGS | LSC |
| 76736 | T(A) | 7 | 76742 | infA-rps8 | IGS | LSC |
| 77087 | T(T) | 7 | 77093 | rps8 | CDS | LSC |
| 77298 | T(T) | 15 | 77312 | rps8-rpl14 | IGS | LSC |
| 77789 | T(A) | 7 | 77795 | rpl14-rpl16 | IGS | LSC |
| 78333 | T(A) | 9 | 78341 | rpl16 | intron | LSC |
| 79780 | T(T) | 7 | 79786 | rps3 | CDS | LSC |
| 79933 | T(C) | 7 | 79939 | rps3-rpl22 | IGS | LSC |
| 80072 | T(T) | 7 | 80078 | rpl22 | CDS | LSC |
| 80911 | T(T) | 7 | 80917 | rpl2 | CDS | IR |
| 84536 | T(A) | 8 | 84543 | ycf2 | CDS | IR |
| 84981 | T(A) | 7 | 84987 | ycf2 | CDS | IR |
| 85559 | T(T) | 7 | 85565 | ycf2 | CDS | IR |
| 86149 | T(A) | 9 | 86157 | ycf2 | CDS | IR |
| 88802 | T(A) | 7 | 88808 | ycf2 | CDS | IR |
| 91764 | T(A) | 8 | 91771 | ndhB(pseudo) | pseudo | IR |
| 91899 | T(A) | 12 | 91910 | ndhB(pseudo) | pseudo | IR |
| 95190 | T(T) | 8 | 95197 | rps12-trnV(GAC) | IGS | IR |
| 96241 | T(A) | 7 | 96247 | rps12-trnV(GAC) | IGS | IR |
| 98901 | T(T) | 8 | 98908 | trnV(GAU) | intron | IR |
| 100138 | T(G) | 7 | 100144 | trnA(UGC) | intron | IR |
| 100297 | T(G) | 7 | 100303 | trnA(UGC) | intron | IR |
| 102737 | T(G) | 7 | 102743 | 23SrRNA | rRNA | IR |
| 104158 | T(T) | 7 | 104164 | 5SrRNA-trnR(ACG) | IGS | IR |
| 104456 | T(T) | 7 | 104462 | trnR(ACG)-trnN(GUU) | IGS | IR |
| 105784 | T(A) | 7 | 105790 | rpl32 | CDS | SSC |
| 107593 | T(A) | 8 | 107600 | ccsA-psaC | IGS | SSC |
| 108332 | T(A) | 8 | 108339 | ccsA-psaC | IGS | SSC |
| 109439 | T(T) | 8 | 109446 | psaC-rps15 | IGS | SSC |
| 109638 | T(T) | 9 | 109646 | rps15 | CDS | SSC |
| 110834 | T(T) | 9 | 110842 | ycf1 | CDS | SSC |
| 111040 | T(T) | 7 | 111046 | ycf1 | CDS | SSC |
| 111172 | T(T) | 8 | 111179 | ycf1 | CDS | SSC |
| 111577 | T(A) | 7 | 111583 | ycf1 | CDS | SSC |
| 111787 | T(A) | 7 | 111793 | ycf1 | CDS | SSC |
| 112774 | T(T) | 7 | 112780 | ycf1 | CDS | SSC |
| 113275 | T(A) | 7 | 113281 | ycf1 | CDS | SSC |
| 116203 | T(A) | 7 | 116209 | trnN(GUU)-trnR(ACG) | IGS | IR |
| 116501 | T(A) | 7 | 116507 | trnR(ACG)-5SrRNA | IGS | IR |
| 117922 | T(C) | 7 | 117928 | 23SrRNA | rRNA | IR |
| 120362 | T(C) | 7 | 120368 | trnA(UGC) | intron | IR |
| 120521 | T(C) | 7 | 120527 | trnA(UGC) | tRNA | IR |
| 121757 | T(A) | 8 | 121764 | trnV(GAU) | intron | IR |
| 124418 | T(T) | 7 | 124424 | trnV(GAU)-rps12 | IGS | IR |
| 125468 | T(A) | 8 | 125475 | trnV(GAU)-rps12 | IGS | IR |
| 128755 | T(T) | 12 | 128766 | ndhB(pseudo) | pseudo | IR |
| 128894 | T(T) | 8 | 128901 | ndhB(pseudo) | pseudo | IR |
| 131857 | T(T) | 7 | 131863 | ycf2 | CDS | IR |
| 134508 | T(T) | 9 | 134516 | ycf2 | CDS | IR |
| 135100 | T(A) | 7 | 135106 | ycf2 | CDS | IR |
| 135678 | T(T) | 7 | 135684 | ycf2 | CDS | IR |
| 136122 | T(T) | 8 | 136129 | ycf2 | CDS | IR |
| 139748 | T(A) | 7 | 139754 | rpl2 | CDS | IR |
| 3662 | T(AT) | 8 | 3669 | trnK(UUU) | intron | LSC |
| 13749 | T(CA) | 8 | 13756 | atpH-atpI | IGS | LSC |
| 19073 | T(CG) | 8 | 19080 | rpoC2 | CDS | LSC |
| 34877 | T(GA) | 8 | 34884 | trnS(UGA) | tRNA | LSC |
| 40523 | T(AG) | 8 | 40530 | psaA | CDS | LSC |
| 41629 | T(TA) | 8 | 41636 | psaA-ycf3 | IGS | LSC |
| 47770 | T(AG) | 8 | 47777 | ndhK(pseudo) | pseudo | LSC |
| 52840 | T(GA) | 8 | 52847 | rbcL | CDS | LSC |
| 54051 | T(TA) | 8 | 54058 | rbcL-accD | IGS | LSC |
| 58238 | T(TC) | 8 | 58245 | cemA | CDS | LSC |
| 59145 | T(AT) | 8 | 59152 | petA | CDS | LSC |
| 81362 | T(TA) | 8 | 81369 | rpl2 | intron | IR |
| 81652 | T(TC) | 8 | 81659 | rpl2 | intron | IR |
| 89484 | T(TA) | 8 | 89491 | ycf2 | CDS | IR |
| 91327 | T(AG) | 8 | 91334 | ndhB(pseudo) | pseudo | IR |
| 102268 | T(CT) | 8 | 102275 | 23SrRNA | rRNA | IR |
| 105166 | T(TA) | 8 | 105173 | trnN(GUU)-ycf1 | IGS | IR |
| 106427 | T(TA) | 8 | 106434 | rpl32-trnL(UAG) | IGS | SSC |
| 115491 | T(AT) | 8 | 115498 | ycf1-trnN(GUU) | IGS | IR |
| 118390 | T(AG) | 8 | 118397 | 23SrRNA | rRNA | IR |
| 129331 | T(CT) | 8 | 129338 | ndhB(pseudo) | pseudo | IR |
| 131174 | T(TA) | 8 | 131181 | ycf2 | CDS | IR |
| 139005 | T(AG) | 8 | 139012 | rpl2 | intron | IR |
| 139295 | T(AT) | 8 | 139302 | rpl2 | intron | IR |
| 9697 | T(AAT) | 18 | 9714 | trnG(UCC)-trnR(UCU) | IGS | LSC |
| 34547 | T(TTC) | 12 | 34558 | psbC | CDS | LSC |
| 65022 | T(ACT) | 12 | 65033 | rps18 | CDS | LSC |
| 4940 | T(GATA) | 12 | 4951 | trnK(UUU)-rps16 | IGS | LSC |
| 41889 | T(ATTA) | 12 | 41900 | psaA-ycf3 | IGS | LSC |
| 4211 | T(AAATA) | 15 | 4225 | trnK(UUU)-rps16 | IGS | LSC |
| 30832 | T(ATATT) | 20 | 30851 | trnE(UUC)-trnT(GGU) | IGS | LSC |

| **Sequence repeats in *Genlisea pygmaea.* Type, length, region, location and plastome quadripartite region. (F = Forward repeats; P= Palindromic repeats; T = Tandem repeats)** | | | | | | | | | | | | | |
| --- | --- | --- | --- | --- | --- | --- | --- | --- | --- | --- | --- | --- | --- |
| Repeat Start 1 | Type | | | Lenght (bp) | | Repeat Start 2 | | Gene | | Location | | Region | |
| 37664 | F | | | 40 | | 39888 | | psaB;psaA | | CDS | | LSC | |
| 8216 | F | | | 30 | | 8242 | | trnS(GCU)-trnG(UCC) | | IGS | | LSC | |
| 91030 | F | | | 33 | | 94074 | | ndhB (pseudo); rps12-trnV(GAC) | | pseudo;IGS | | IR | |
| 126247 | F | | | 33 | | 129291 | | trnV(GAC)-rps12; ndhB(pseudo) | | IGS; pseudo | | IR | |
| 8046 | F | | | 30 | | 34526 | | psbI-trnS(GCU);psbC-trnS(UGA) | | IGS | | LSC | |
| 9637 | F | | | 30 | | 35460 | | trnG(UCC);trnG(UCC) | | tRNA | | LSC | |
| 41624 | F | | | 30 | | 109733 | | psaA-ycf3;ndhE(pseudo)-rps15 | | IGS | | LSC | |
| 63356 | P | | | 69 | | 63356 | | psaJ-rpl33 | | IGS | | LSC | |
| 29091 | P | | | 38 | | 29091 | | psbM-trnD(GUC) | | IGS | | LSC | |
| 41133 | P | | | 44 | | 41133 | | psaA-ycf3 | | IGS | | LSC | |
| 56453 | P | | | 36 | | 56453 | | psaI-ycf4 | | IGS | | LSC | |
| 8046 | P | | | 30 | | 44233 | | trnS(GCU);trnS(GGA) | | tRNA | | LSC | |
| 91030 | P | | | 33 | | 126247 | | ndhB(pseudo);trnV(GAC)-rps12 | | pseudo;IGS | | IR | |
| 94074 | P | | | 33 | | 129291 | | rps12-trnV(GAC);ndhB(pseudo) | | IGS;pseudo | | IR | |
| 109711 | P | | | 30 | | 109711 | | ndhE(pseudo)-rps15 | | IGS | | SSC | |
| 34526 | P | | | 30 | | 44233 | | psbC-trnS(UGA);trnS(GGA) | | IGS;tRNA | | LSC | |
| 1549 | T(A) | | | 9 | | 1557 | | psbA-trnK(UUU) | | IGS | | LSC | |
| 2302 | T(T) | | | 10 | | 2311 | | matK | | CDS | | LSC | |
| 3890 | T(G) | | | 7 | | 3896 | | trnK(UUU) | | intron | | LSC | |
| 7395 | T(T) | | | 7 | | 7401 | | psbK | | CDS | | LSC | |
| 8185 | T(T) | | | 15 | | 8199 | | trnS(GCU)-trnG(UCC) | | IGS | | LSC | |
| 8320 | T(A) | | | 7 | | 8326 | | trnS(GCU)-trnG(UCC) | | IGS | | LSC | |
| 8792 | T(A) | | | 7 | | 8798 | | trnS(GCU)-trnG(UCC) | | IGS | | LSC | |
| 12670 | T(A) | | | 8 | | 12677 | | atpF | | CDS | | LSC | |
| 13088 | T(A) | | | 8 | | 13095 | | atpF-atpH | | IGS | | LSC | |
| 13525 | T(T) | | | 7 | | 13531 | | atpH-atpI | | IGS | | LSC | |
| 13820 | T(T) | | | 7 | | 13826 | | atpH-atpI | | IGS | | LSC | |
| 16123 | T(T) | | | 8 | | 16130 | | rpoC2 | | CDS | | LSC | |
| 17840 | T(T) | | | 7 | | 17846 | | rpoC2 | | CDS | | LSC | |
| 18108 | T(A) | | | 9 | | 18116 | | rpoC2 | | CDS | | LSC | |
| 20405 | T(T) | | | 7 | | 20411 | | rpoC1 | | CDS | | LSC | |
| 20596 | T(A) | | | 8 | | 20603 | | rpoC1 | | CDS | | LSC | |
| 22239 | T(A) | | | 14 | | 22252 | | rpoC1 | | intron | | LSC | |
| 25242 | T(A) | | | 7 | | 25248 | | rpoB | | CDS | | LSC | |
| 26814 | T(A) | | | 7 | | 26820 | | rpoB-trnC(GCA) | | IGS | | LSC | |
| 27954 | T(T) | | | 9 | | 27962 | | trnC(GCA)-petN | | IGS | | LSC | |
| 28393 | T(G) | | | 10 | | 28402 | | petN-psbM | | IGS | | LSC | |
| 29338 | T(T) | | | 7 | | 29344 | | psbM-trnD(GUC) | | IGS | | LSC | |
| 29470 | T(T) | | | 9 | | 29478 | | psbM-trnD(GUC) | | IGS | | LSC | |
| 30646 | T(T) | | | 7 | | 30652 | | trnT(GGU)-psbD | | IGS | | LSC | |
| 31639 | T(T) | | | 7 | | 31645 | | trnT(GGU)-psbD | | IGS | | LSC | |
| 33211 | T(G) | | | 7 | | 33217 | | psbC | | CDS | | LSC | |
| 33961 | T(G) | | | 7 | | 33967 | | psbC | | CDS | | LSC | |
| 34421 | T(T) | | | 7 | | 34427 | | psbC-trnS(UGA) | | IGS | | LSC | |
| 35637 | T(A) | | | 9 | | 35645 | | trnG(UCC)-trnfM(CAU) | | IGS | | LSC | |
| 38773 | T(A) | | | 7 | | 38779 | | psaA | | CDS | | LSC | |
| 39259 | T(C) | | | 7 | | 39265 | | psaA | | CDS | | LSC | |
| 39999 | T(A) | | | 7 | | 40005 | | psaA | | CDS | | LSC | |
| 41353 | T(T) | | | 7 | | 41359 | | psaA-ycf3 | | IGS | | LSC | |
| 42041 | T(T) | | | 9 | | 42049 | | ycf3 | | intron | | LSC | |
| 42367 | T(T) | | | 13 | | 42379 | | ycf3 | | intron | | LSC | |
| 46028 | T(A) | | | 11 | | 46038 | | trnL(UAA) | | intron | | LSC | |
| 46663 | T(T) | | | 13 | | 46675 | | trnL(UAA)-trnF(GAA) | | IGS | | LSC | |
| 47200 | T(T) | | | 7 | | 47206 | | ndhK(pseudo) | | pseudo | | LSC | |
| 47693 | T(A) | | | 8 | | 47700 | | ndhC(pseudo) | | pseudo | | LSC | |
| 47848 | T(A) | | | 7 | | 47854 | | ndhC(pseudo)-trnC(UAC) | | IGS | | LSC | |
| 48813 | T(T) | | | 7 | | 48819 | | trnV(UAC)-trnM(CAU) | | IGS | | LSC | |
| 49070 | T(T) | | | 14 | | 49083 | | trnM(CAU)-atpE | | IGS | | LSC | |
| 51499 | T(T) | | | 13 | | 51511 | | atpB-rbcL | | IGS | | LSC | |
| 57383 | T(T) | | | 9 | | 57391 | | ycf4-cemA | | IGS | | LSC | |
| 58460 | T(T) | | | 7 | | 58466 | | cemA-petA | | IGS | | LSC | |
| 59063 | T(A) | | | 7 | | 59069 | | petA | | CDS | | LSC | |
| 59534 | T(T) | | | 7 | | 59540 | | petA | | CDS | | LSC | |
| 60107 | T(T) | | | 11 | | 60117 | | petA-psbJ | | IGS | | LSC | |
| 60263 | T(A) | | | 9 | | 60271 | | petA-psbJ | | IGS | | LSC | |
| 60926 | T(A) | | | 8 | | 60933 | | psbF | | CDS | | LSC | |
| 62335 | T(A) | | | 12 | | 62346 | | petL-petG | | IGS | | LSC | |
| 62490 | T(T) | | | 7 | | 62496 | | petG-trnW(CCA) | | IGS | | LSC | |
| 62689 | T(A) | | | 7 | | 62695 | | trnW(CCA)-trnP(UGG) | | IGS | | LSC | |
| 63259 | T(T) | | | 7 | | 63265 | | psaJ | | CDS | | LSC | |
| 65617 | T(T) | | | 12 | | 65628 | | rpl20-rps12 | | IGS | | LSC | |
| 65771 | T(T) | | | 7 | | 65777 | | rpl20-rps12 | | IGS | | LSC | |
| 66977 | T(A) | | | 13 | | 66989 | | clpP | | intron | | LSC | |
| 67358 | T(A) | | | 7 | | 67364 | | clpP | | CDS | | LSC | |
| 67471 | T(A) | | | 8 | | 67478 | | clpP | | intron | | LSC | |
| 69313 | T(T) | | | 8 | | 69320 | | psbB | | CDS | | LSC | |
| 69662 | T(T) | | | 7 | | 69668 | | psbB | | CDS | | LSC | |
| 70116 | T(T) | | | 8 | | 70123 | | psbB-psbT | | IGS | | LSC | |
| 70312 | T(T) | | | 7 | | 70318 | | psbT | | CDS | | LSC | |
| 71222 | T(A) | | | 7 | | 71228 | | petB | | intron | | LSC | |
| 71420 | T(A) | | | 10 | | 71429 | | petB | | intron | | LSC | |
| 74977 | T(T) | | | 7 | | 74983 | | rps11 | | CDS | | LSC | |
| 75441 | T(T) | | | 7 | | 75447 | | rps11-rpl36 | | IGS | | LSC | |
| 76022 | T(A) | | | 7 | | 76028 | | infA-rps8 | | IGS | | LSC | |
| 76373 | T(T) | | | 7 | | 76379 | | rps8 | | CDS | | LSC | |
| 77065 | T(A) | | | 7 | | 77071 | | rpl14-rpl16 | | IGS | | LSC | |
| 77609 | T(A) | | | 8 | | 77616 | | rpl16 | | intron | | LSC | |
| 77758 | T(T) | | | 9 | | 77766 | | rpl16 | | intron | | LSC | |
| 79016 | T(T) | | | 7 | | 79022 | | rps3 | | CDS | | LSC | |
| 79306 | T(T) | | | 7 | | 79312 | | rpl22 | | CDS | | LSC | |
| 80145 | T(T) | | | 7 | | 80151 | | rpl2 | | CDS | | IR | |
| 83779 | T(A) | | | 8 | | 83786 | | ycf2 | | CDS | | IR | |
| 84224 | T(A) | | | 7 | | 84230 | | ycf2 | | CDS | | IR | |
| 84802 | T(T) | | | 7 | | 84808 | | ycf2 | | CDS | | IR | |
| 85392 | T(A) | | | 9 | | 85400 | | ycf2 | | CDS | | IR | |
| 88045 | T(A) | | | 7 | | 88051 | | ycf2 | | CDS | | IR | |
| 91024 | T(A) | | | 9 | | 91032 | | ndhB(pseudo) | | pseudo | | IR | |
| 91160 | T(A) | | | 11 | | 91170 | | ndhB(pseudo) | | pseudo | | IR | |
| 94426 | T(T) | | | 8 | | 94433 | | rps12-trnV(GAC) | | IGS | | IR | |
| 95478 | T(A) | | | 7 | | 95484 | | rps12-trnV(GAC) | | IGS | | IR | |
| 97933 | T(G) | | | 7 | | 97939 | | trnI(GAU) | | intron | | IR | |
| 98138 | T(T) | | | 8 | | 98145 | | trnI(GAU) | | intron | | IR | |
| 99375 | T(G) | | | 7 | | 99381 | | trnA(UGC) | | intron | | IR | |
| 99534 | T(G) | | | 7 | | 99540 | | trnA(UGC) | | intron | | IR | |
| 101974 | T(G) | | | 7 | | 101980 | | 23SrRNA | | rRNA | | IR | |
| 103389 | T(T) | | | 7 | | 103395 | | 5SrRNA-trnR(ACG) | | IGS | | IR | |
| 103687 | T(T) | | | 7 | | 103693 | | trnR(ACG)-trnN(GUU) | | IGS | | IR | |
| 105777 | T(A) | | | 10 | | 105786 | | rpl32-trnL(UAG) | | IGS | | SSC | |
| 107139 | T(A) | | | 7 | | 107145 | | ccsA-psaC | | IGS | | SSC | |
| 107378 | T(T) | | | 7 | | 107384 | | ccsA-psaC | | IGS | | SSC | |
| 109023 | T(A) | | | 8 | | 109030 | | ndhE(pseudo)-rps15 | | IGS | | SSC | |
| 109919 | T(T) | | | 7 | | 109925 | | ndhE(pseudo)-rps15 | | IGS | | SSC | |
| 110126 | T(T) | | | 9 | | 110134 | | rps15 | | CDS | | SSC | |
| 111305 | T(T) | | | 8 | | 111312 | | ycf1 | | CDS | | SSC | |
| 111643 | T(T) | | | 7 | | 111649 | | ycf1 | | CDS | | SSC | |
| 111813 | T(T) | | | 7 | | 111819 | | ycf1 | | CDS | | SSC | |
| 112048 | T(A) | | | 7 | | 112054 | | ycf1 | | CDS | | SSC | |
| 112234 | T(A) | | | 11 | | 112244 | | ycf1 | | CDS | | SSC | |
| 112685 | T(T) | | | 7 | | 112691 | | ycf1 | | CDS | | SSC | |
| 113125 | T(T) | | | 13 | | 113137 | | ycf1 | | CDS | | SSC | |
| 113740 | T(A) | | | 7 | | 113746 | | ycf1 | | CDS | | SSC | |
| 114573 | T(T) | | | 7 | | 114579 | | ycf1 | | CDS | | SSC | |
| 114732 | T(A) | | | 7 | | 114738 | | ycf1 | | CDS | | SSC | |
| 116662 | T(A) | | | 7 | | 116668 | | trnN(GUU)-trnR(ACG) | | IGS | | IR | |
| 116960 | T(A) | | | 7 | | 116966 | | trnR(ACG)-5SrRNA | | IGS | | IR | |
| 118375 | T(C) | | | 7 | | 118381 | | 23SrRNA | | rRNA | | IR | |
| 120815 | T(C) | | | 7 | | 120821 | | trnA(UGC) | | intron | | IR | |
| 120974 | T(C) | | | 7 | | 120980 | | trnA(UGC) | | tRNA | | IR | |
| 122210 | T(A) | | | 8 | | 122217 | | trnI(GAU) | | intron | | IR | |
| 122416 | T(C) | | | 7 | | 122422 | | trnI(GAU) | | intron | | IR | |
| 124871 | T(T) | | | 7 | | 124877 | | trnV(GAC)-rps12 | | IGS | | IR | |
| 125922 | T(A) | | | 8 | | 125929 | | trnV(GAC)-rps12 | | IGS | | IR | |
| 129185 | T(T) | | | 11 | | 129195 | | ndhB(pseudo) | | pseudo | | IR | |
| 129323 | T(T) | | | 9 | | 129331 | | ndhB(pseudo) | | pseudo | | IR | |
| 132304 | T(T) | | | 7 | | 132310 | | ycf2 | | CDS | | IR | |
| 134955 | T(T) | | | 9 | | 134963 | | ycf2 | | CDS | | IR | |
| 135547 | T(A) | | | 7 | | 135553 | | ycf2 | | CDS | | IR | |
| 136125 | T(T) | | | 7 | | 136131 | | ycf2 | | CDS | | IR | |
| 136569 | T(T) | | | 8 | | 136576 | | ycf2 | | CDS | | IR | |
| 140204 | T(A) | | | 7 | | 140210 | | rpl2 | | CDS | | IR | |
| 3722 | T(AT) | | | 8 | | 3729 | | trnK(UUU) | | intron | | LSC | |
| 18850 | T(CG) | | | 8 | | 18857 | | rpoC2 | | CDS | | LSC | |
| 34530 | T(GA) | | | 8 | | 34537 | | trnS(UGA) | | tRNA | | LSC | |
| 40158 | T(AG) | | | 8 | | 40165 | | psaA | | CDS | | LSC | |
| 52422 | T(GA) | | | 8 | | 52429 | | rbcL | | CDS | | LSC | |
| 53648 | T(TA) | | | 8 | | 53655 | | rbcL-accD | | IGS | | LSC | |
| 57796 | T(TC) | | | 8 | | 57803 | | cemA | | CDS | | LSC | |
| 58702 | T(AT) | | | 8 | | 58709 | | petA | | CDS | | LSC | |
| 80596 | T(TA) | | | 8 | | 80603 | | rpl2 | | intron | | IR | |
| 80886 | T(TC) | | | 8 | | 80893 | | rpl2 | | intron | | IR | |
| 88727 | T(TA) | | | 8 | | 88734 | | ycf2 | | CDS | | IR | |
| 90579 | T(AG) | | | 8 | | 90586 | | ndhB(pseudo) | | pseudo | | IR | |
| 101505 | T(CT) | | | 8 | | 101512 | | 23SrRNA | | rRNA | | IR | |
| 110665 | T(TA) | | | 8 | | 110672 | | rps15-ycf1 | | IGS | | IR | |
| 118843 | T(AG) | | | 8 | | 118850 | | 23SrRNA | | rRNA | | IR | |
| 129769 | T(CT) | | | 8 | | 129776 | | ndhB(pseudo) | | pseudo | | IR | |
| 131621 | T(TA) | | | 8 | | 131628 | | ycf2 | | CDS | | IR | |
| 139461 | T(AG) | | | 8 | | 139468 | | rpl2 | | intron | | IR | |
| 139751 | T(AT) | | | 8 | | 139758 | | rpl2 | | intron | | IR | |
| 34210 | T(TTC) | | | 12 | | 34221 | | psbC | | CDS | | LSC | |
| 1665 | T(CTAA) | | | 12 | | 1676 | | psbA-trnK(UUU) | | IGS | | LSC | |
| 53393 | T(GTAATT) | | | 18 | | 53410 | | rbcL-accD | | IGS | | LSC | |
| **Sequence repeats in *Genlisea tuberosa.* Type, length, region, location and plastome quadripartite region. (F = Forward repeats; P= Palindromic repeats; T = Tandem repeats)** | | | | | | | | | | | | |  |
| Repeat Start 1 | | Type | Lenght (bp) | | Repeat Start 2 | | Gene | | Location | | Region | |  |
| 63271 | | F | 44 | | 63301 | | trnP(UGG)-psaJ | | IGS | | LSC | |  |
| 106090 | | F | 36 | | 106125 | | rpl32-trnL(UAG) | | IGS | | SSC | |  |
| 37977 | | F | 40 | | 40201 | | psaB;psaA | | CDS | | LSC | |  |
| 60590 | | F | 31 | | 60623 | | petA-psbJ | | IGS | | LSC | |  |
| 8023 | | F | 30 | | 34840 | | psbI-trnS(GCU);psbC-trnS(UGA) | | IGS | | LSC | |  |
| 9588 | | F | 30 | | 35766 | | trnG(UCC);trnG(UCC) | | tRNA | | LSC | |  |
| 68003 | | F | 30 | | 68029 | | clpP | | intron | | LSC | |  |
| 63684 | | P | 62 | | 63684 | | psaJ-rpl33 | | IGS | | LSC | |  |
| 8508 | | P | 42 | | 8508 | | trnS(GCU)-trnG(UCC) | | IGS | | LSC | |  |
| 56782 | | P | 36 | | 56782 | | psaI-ycf4 | | IGS | | LSC | |  |
| 8023 | | P | 30 | | 44653 | | psbI-trnS(GCU);trnS(GGA) | | IGS;tRNA | | LSC | |  |
| 66506 | | P | 34 | | 66506 | | rps12-clpP | | IGS | | LSC | |  |
| 34840 | | P | 30 | | 44653 | | psbC-trnS(UGA); trnS(GGA) | | IGS;tRNA | | LSC | |  |
| 41950 | | P | 30 | | 78611 | | psaA-ycf3; rpl16 | | IGS;intron | | LSC | |  |
| 2230 | | T(T) | 10 | | 2239 | | matK | | CDS | | LSC | |  |
| 2929 | | T(T) | 8 | | 2936 | | matK | | CDS | | LSC | |  |
| 7356 | | T(T) | 8 | | 7363 | | psbK | | CDS | | LSC | |  |
| 8162 | | T(T) | 9 | | 8170 | | trnS(GCU)-trnG(UCC) | | IGS | | LSC | |  |
| 8321 | | T(T) | 9 | | 8329 | | trnS(GCU)-trnG(UCC) | | IGS | | LSC | |  |
| 8730 | | T(A) | 7 | | 8736 | | trnS(GCU)-trnG(UCC) | | IGS | | LSC | |  |
| 9848 | | T(T) | 7 | | 9854 | | trnR(UCU)-atpA | | IGS | | LSC | |  |
| 12672 | | T(A) | 8 | | 12679 | | atpF | | CDS | | LSC | |  |
| 13151 | | T(A) | 8 | | 13158 | | atpF-atpH | | IGS | | LSC | |  |
| 15224 | | T(A) | 7 | | 15230 | | atpI-rps2 | | IGS | | LSC | |  |
| 16342 | | T(T) | 8 | | 16349 | | rpoC2 | | CDS | | LSC | |  |
| 18068 | | T(T) | 7 | | 18074 | | rpoC2 | | CDS | | LSC | |  |
| 18336 | | T(A) | 9 | | 18344 | | rpoC2 | | CDS | | LSC | |  |
| 20621 | | T(T) | 7 | | 20627 | | rpoC1 | | CDS | | LSC | |  |
| 20812 | | T(A) | 8 | | 20819 | | rpoC1 | | CDS | | LSC | |  |
| 22460 | | T(A) | 12 | | 22471 | | rpoC1 | | intron | | LSC | |  |
| 22665 | | T(A) | 8 | | 22672 | | rpoC1 | | intron | | LSC | |  |
| 25462 | | T(A) | 7 | | 25468 | | rpoB | | CDS | | LSC | |  |
| 27032 | | T(A) | 7 | | 27038 | | rpoB-trnC(GCA) | | IGS | | LSC | |  |
| 27318 | | T(T) | 17 | | 27334 | | rpoB-trnC(GCA) | | IGS | | LSC | |  |
| 27528 | | T(T) | 7 | | 27534 | | rpoB-trnC(GCA) | | IGS | | LSC | |  |
| 27887 | | T(A) | 8 | | 27894 | | trnC(GCA)-petN | | IGS | | LSC | |  |
| 28214 | | T(T) | 9 | | 28222 | | trnC(GCA)-petN | | IGS | | LSC | |  |
| 29595 | | T(T) | 7 | | 29601 | | psbM-trnD(GUC) | | IGS | | LSC | |  |
| 29722 | | T(T) | 12 | | 29733 | | psbM-trnD(GUC) | | IGS | | LSC | |  |
| 31942 | | T(T) | 7 | | 31948 | | trnT(GGU)-psbD | | IGS | | LSC | |  |
| 32141 | | T(C) | 7 | | 32147 | | trnT(GGU)-psbD | | IGS | | LSC | |  |
| 33515 | | T(G) | 7 | | 33521 | | psbC | | CDS | | LSC | |  |
| 34265 | | T(G) | 7 | | 34271 | | psbC | | CDS | | LSC | |  |
| 34732 | | T(T) | 7 | | 34738 | | psbC-trnS(UGA) | | IGS | | LSC | |  |
| 35948 | | T(A) | 7 | | 35954 | | trnG(UCC)-trnfM(CAU) | | IGS | | LSC | |  |
| 39086 | | T(A) | 7 | | 39092 | | psaA | | CDS | | LSC | |  |
| 39572 | | T(C) | 7 | | 39578 | | psaA | | CDS | | LSC | |  |
| 40312 | | T(A) | 7 | | 40318 | | psaA | | CDS | | LSC | |  |
| 41314 | | T(T) | 11 | | 41324 | | psaA-ycf3 | | IGS | | LSC | |  |
| 42416 | | T(T) | 8 | | 42423 | | ycf3 | | intron | | LSC | |  |
| 42756 | | T(T) | 11 | | 42766 | | ycf3 | | intron | | LSC | |  |
| 42885 | | T(T) | 9 | | 42893 | | ycf3 | | intron | | LSC | |  |
| 45723 | | T(A) | 8 | | 45730 | | rps4-trnT(UGU) | | IGS | | LSC | |  |
| 46372 | | T(A) | 9 | | 46380 | | trnL(UAA) | | intron | | LSC | |  |
| 47009 | | T(T) | 8 | | 47016 | | trnL(UAA)-trnF(GAA) | | IGS | | LSC | |  |
| 48190 | | T(A) | 9 | | 48198 | | ndhC(pseudo)-trnV(UAC) | | IGS | | LSC | |  |
| 49418 | | T(T) | 14 | | 49431 | | trnM(CAU)-atpE | | IGS | | LSC | |  |
| 51847 | | T(T) | 11 | | 51857 | | atpB-rbcL | | IGS | | LSC | |  |
| 53826 | | T(T) | 7 | | 53832 | | rbcL-accD | | IGS | | LSC | |  |
| 54122 | | T(A) | 9 | | 54130 | | rbcL-accD | | IGS | | LSC | |  |
| 57523 | | T(T) | 14 | | 57536 | | ycf4-cemA | | IGS | | LSC | |  |
| 59234 | | T(A) | 7 | | 59240 | | petA | | CDS | | LSC | |  |
| 59705 | | T(T) | 7 | | 59711 | | petA | | CDS | | LSC | |  |
| 59834 | | T(A) | 7 | | 59840 | | petA-psbJ | | IGS | | LSC | |  |
| 59969 | | T(T) | 7 | | 59975 | | petA-psbJ | | IGS | | LSC | |  |
| 60255 | | T(T) | 17 | | 60271 | | petA-psbJ | | IGS | | LSC | |  |
| 60571 | | T(A) | 7 | | 60577 | | petA-psbJ | | IGS | | LSC | |  |
| 61161 | | T(A) | 8 | | 61168 | | psbF | | CDS | | LSC | |  |
| 62045 | | T(A) | 7 | | 62051 | | psbE-petL | | IGS | | LSC | |  |
| 62308 | | T(A) | 8 | | 62315 | | psbE-petL | | IGS | | LSC | |  |
| 62758 | | T(T) | 7 | | 62764 | | petG-trnW(CCA) | | IGS | | LSC | |  |
| 62957 | | T(A) | 7 | | 62963 | | trnW(CCA)-trnP(UGG) | | IGS | | LSC | |  |
| 65958 | | T(T) | 7 | | 65964 | | rpl20-rps12 | | IGS | | LSC | |  |
| 66107 | | T(T) | 9 | | 66115 | | rpl20-rps12 | | IGS | | LSC | |  |
| 67322 | | T(A) | 11 | | 67332 | | clpP | | intron | | LSC | |  |
| 67701 | | T(A) | 7 | | 67707 | | clpP | | CDS | | LSC | |  |
| 67815 | | T(A) | 7 | | 67821 | | clpP | | intron | | LSC | |  |
| 68196 | | T(T) | 9 | | 68204 | | clpP | | intron | | LSC | |  |
| 68397 | | T(T) | 7 | | 68403 | | clpP | | intron | | LSC | |  |
| 69705 | | T(T) | 8 | | 69712 | | psbB | | CDS | | LSC | |  |
| 70054 | | T(T) | 7 | | 70060 | | psbB | | CDS | | LSC | |  |
| 71391 | | T(T) | 7 | | 71397 | | psbH-petB | | IGS | | LSC | |  |
| 71632 | | T(A) | 8 | | 71639 | | psbH-petB | | IGS | | LSC | |  |
| 71812 | | T(A) | 13 | | 71824 | | petB | | intron | | LSC | |  |
| 75400 | | T(T) | 7 | | 75406 | | rps11 | | CDS | | LSC | |  |
| 75864 | | T(T) | 7 | | 75870 | | rps11-rpl36 | | IGS | | LSC | |  |
| 76436 | | T(A) | 7 | | 76442 | | infA-rps8 | | IGS | | LSC | |  |
| 76787 | | T(T) | 7 | | 76793 | | rps8 | | CDS | | LSC | |  |
| 77488 | | T(A) | 7 | | 77494 | | rpl14-rpl16 | | IGS | | LSC | |  |
| 78032 | | T(A) | 9 | | 78040 | | rpl16 | | intron | | LSC | |  |
| 79471 | | T(T) | 7 | | 79477 | | rps3 | | CDS | | LSC | |  |
| 79624 | | T(C) | 8 | | 79631 | | rps3-rpl22 | | IGS | | LSC | |  |
| 79764 | | T(T) | 7 | | 79770 | | rpl22 | | CDS | | LSC | |  |
| 80603 | | T(T) | 7 | | 80609 | | rpl2 | | CDS | | IR | |  |
| 84228 | | T(A) | 8 | | 84235 | | ycf2 | | CDS | | IR | |  |
| 84673 | | T(A) | 7 | | 84679 | | ycf2 | | CDS | | IR | |  |
| 85251 | | T(T) | 7 | | 85257 | | ycf2 | | CDS | | IR | |  |
| 85841 | | T(A) | 9 | | 85849 | | ycf2 | | CDS | | IR | |  |
| 88494 | | T(A) | 7 | | 88500 | | ycf2 | | CDS | | IR | |  |
| 91452 | | T(A) | 8 | | 91459 | | ndhB(pseudo) | | pseudo | | IR | |  |
| 91587 | | T(A) | 14 | | 91600 | | ndhB(pseudo) | | pseudo | | IR | |  |
| 94880 | | T(T) | 8 | | 94887 | | rps12-trnV(GAC) | | IGS | | IR | |  |
| 95931 | | T(A) | 7 | | 95937 | | rps12-trnV(GAC) | | IGS | | IR | |  |
| 98591 | | T(T) | 8 | | 98598 | | trnI(GAU) | | intron | | IR | |  |
| 99828 | | T(G) | 7 | | 99834 | | trnA(UGC) | | intron | | IR | |  |
| 99987 | | T(G) | 7 | | 99993 | | trnA(UGC) | | intron | | IR | |  |
| 102427 | | T(G) | 7 | | 102433 | | 23SrRNA | | rRNA | | IR | |  |
| 103848 | | T(T) | 7 | | 103854 | | 5SrRNA-trnR(ACG) | | IGS | | IR | |  |
| 105469 | | T(A) | 7 | | 105475 | | rpl32 | | CDS | | SSC | |  |
| 107303 | | T(A) | 13 | | 107315 | | ccsA-psaC | | IGS | | SSC | |  |
| 107748 | | T(A) | 14 | | 107761 | | ccsA-psaC | | IGS | | SSC | |  |
| 108060 | | T(A) | 12 | | 108071 | | ccsA-psaC | | IGS | | SSC | |  |
| 109127 | | T(A) | 8 | | 109134 | | psaC-ndhI(pseudo) | | IGS | | SSC | |  |
| 110079 | | T(T) | 10 | | 110088 | | ndhI(pseudo)-rps15 | | IGS | | SSC | |  |
| 110280 | | T(T) | 9 | | 110288 | | rps15 | | CDS | | SSC | |  |
| 111475 | | T(T) | 9 | | 111483 | | ycf1 | | CDS | | IR | |  |
| 111681 | | T(T) | 7 | | 111687 | | ycf1 | | CDS | | IR | |  |
| 111813 | | T(T) | 8 | | 111820 | | ycf1 | | CDS | | IR | |  |
| 112218 | | T(A) | 7 | | 112224 | | ycf1 | | CDS | | IR | |  |
| 112428 | | T(A) | 7 | | 112434 | | ycf1 | | CDS | | IR | |  |
| 113430 | | T(T) | 7 | | 113436 | | ycf1 | | CDS | | IR | |  |
| 113931 | | T(A) | 7 | | 113937 | | ycf1 | | CDS | | IR | |  |
| 115276 | | T(T) | 9 | | 115284 | | ycf1 | | CDS | | IR | |  |
| 117171 | | T(A) | 7 | | 117177 | | trnR(ACG)-5SrRNA | | IGS | | IR | |  |
| 118592 | | T(C) | 7 | | 118598 | | 23SrRNA | | rRNA | | IR | |  |
| 121032 | | T(C) | 7 | | 121038 | | trnA(UGC) | | intron | | IR | |  |
| 121191 | | T(C) | 7 | | 121197 | | trnA(UGC) | | intron | | IR | |  |
| 122427 | | T(A) | 8 | | 122434 | | trnI(GAU) | | intron | | IR | |  |
| 125088 | | T(T) | 7 | | 125094 | | trnV(GAC)-rps12 | | IGS | | IR | |  |
| 126138 | | T(A) | 8 | | 126145 | | trnV(GAC)-rps12 | | IGS | | IR | |  |
| 129425 | | T(T) | 14 | | 129438 | | ndhB(pseudo) | | pseudo | | IR | |  |
| 129566 | | T(T) | 8 | | 129573 | | ndhB(pseudo) | | pseudo | | IR | |  |
| 132525 | | T(T) | 7 | | 132531 | | ycf2 | | CDS | | IR | |  |
| 135176 | | T(T) | 9 | | 135184 | | ycf2 | | CDS | | IR | |  |
| 135768 | | T(A) | 7 | | 135774 | | ycf2 | | CDS | | IR | |  |
| 136346 | | T(T) | 7 | | 136352 | | ycf2 | | CDS | | IR | |  |
| 136790 | | T(T) | 8 | | 136797 | | ycf2 | | CDS | | IR | |  |
| 140416 | | T(A) | 7 | | 140422 | | rpl2 | | CDS | | IR | |  |
| 3658 | | T(AT) | 8 | | 3665 | | trnK(UUU) | | intron | | LSC | |  |
| 5590 | | T(GC) | 8 | | 5597 | | rps16 | | intron | | LSC | |  |
| 13754 | | T(CA) | 8 | | 13761 | | atpH-atpI | | IGS | | LSC | |  |
| 19066 | | T(CG) | 8 | | 19073 | | rpoC2 | | CDS | | LSC | |  |
| 30949 | | T(CT) | 8 | | 30956 | | trnT(GGU) | | tRNA | | LSC | |  |
| 34844 | | T(GA) | 8 | | 34851 | | trnS(UGA) | | tRNA | | LSC | |  |
| 40471 | | T(AG) | 8 | | 40478 | | psaA | | CDS | | LSC | |  |
| 41578 | | T(TA) | 8 | | 41585 | | psaA-ycf3 | | IGS | | LSC | |  |
| 52756 | | T(GA) | 8 | | 52763 | | rbcL | | CDS | | LSC | |  |
| 53967 | | T(TA) | 8 | | 53974 | | rbcL-accD | | IGS | | LSC | |  |
| 57966 | | T(TC) | 8 | | 57973 | | cemA | | CDS | | LSC | |  |
| 58873 | | T(AT) | 8 | | 58880 | | petA | | CDS | | LSC | |  |
| 81054 | | T(TA) | 8 | | 81061 | | rpl2 | | intron | | IR | |  |
| 81344 | | T(TC) | 8 | | 81351 | | rpl2 | | intron | | IR | |  |
| 89176 | | T(TA) | 8 | | 89183 | | ycf2 | | CDS | | IR | |  |
| 91015 | | T(AG) | 8 | | 91022 | | ndhB(pseudo) | | pseudo | | IR | |  |
| 101958 | | T(CT) | 8 | | 101965 | | 23SrRNA | | rRNA | | IR | |  |
| 104856 | | T(TA) | 10 | | 104865 | | trnN(GUU)-ycf1 | | IGS | | IR | |  |
| 116159 | | T(AT) | 10 | | 116168 | | ycf1-trnN(GUU) | | IGS | | IR | |  |
| 119060 | | T(AG) | 8 | | 119067 | | 23SrRNA | | rRNA | | IR | |  |
| 130003 | | T(CT) | 8 | | 130010 | | ndhB(pseudo) | | pseudo | | IR | |  |
| 131842 | | T(TA) | 8 | | 131849 | | ycf2 | | CDS | | IR | |  |
| 139673 | | T(AG) | 8 | | 139680 | | rpl2 | | intron | | IR | |  |
| 139963 | | T(AT) | 8 | | 139970 | | rpl2 | | intron | | IR | |  |
| 9693 | | T(AAT) | 18 | | 9710 | | trnG(UCC)-trnR(UCU) | | IGS | | LSC | |  |
| 34514 | | T(TTC) | 12 | | 34525 | | psbC | | CDS | | LSC | |  |
| 64775 | | T(ACT) | 12 | | 64786 | | rps18 | | CDS | | LSC | |  |
| 41838 | | T(ATTA) | 12 | | 41849 | | psaA-ycf3 | | IGS | | LSC | |  |
| 4202 | | T(AAATA) | 15 | | 4216 | | trnK(UUU)-rps16 | | IGS | | LSC | |  |
| 30784 | | T(ATATT) | 15 | | 30798 | | trnE(UUC)-trnT(GGU) | | IGS | | LSC | |  |

| **Sequence repeats in *Genlisea filiformis.* Type, length, region, location and plastome quadripartite region. (F = Forward repeats; P= Palindromic repeats; T = Tandem repeats)** | | | | | | |
| --- | --- | --- | --- | --- | --- | --- |
| Repeat Start 1 | Type | Lenght (bp) | Repeat Start 2 | Gene | Location | Region |
| 37666 | F | 40 | 39890 | psaB;psaA | CDS | LSC |
| 8062 | F | 30 | 34534 | psbI-trnS(GCU);psbC-trnS(UGA) | IGS | LSC |
| 9599 | F | 30 | 35460 | trnG(UCC);trnG(UCC) | tRNA | LSC |
| 63273 | P | 62 | 63273 | psaJ-rpl33 | IGS | LSC |
| 41135 | P | 44 | 41135 | psaA-ycf3 | IGS | LSC |
| 56407 | P | 36 | 56407 | psaI-ycf4 | IGS | LSC |
| 8062 | P | 30 | 44259 | psbI-trnS(GCU);trnS(GGA) | intron;tRNA | LSC |
| 34534 | P | 30 | 44259 | psbC-trnS(UGA);trnS(GGA) | intron;tRNA | LSC |
| 1604 | T(A) | 7 | 1610 | psbA-trnK(UUU) | IGS | LSC |
| 5823 | T(T) | 8 | 5830 | rps16 | intron | LSC |
| 7404 | T(T) | 7 | 7410 | psbK | CDS | LSC |
| 7809 | T(T) | 11 | 7819 | psbK-psbI | IGS | LSC |
| 8758 | T(A) | 7 | 8764 | trnS(GCU)-trnG(UCC) | IGS | LSC |
| 9836 | T(T) | 7 | 9842 | trnR(UCU)-atpA | IGS | LSC |
| 12646 | T(A) | 8 | 12653 | atpF | CDS | LSC |
| 13062 | T(A) | 7 | 13068 | atpF-atpH | IGS | LSC |
| 13504 | T(T) | 7 | 13510 | atpH-atpI | IGS | LSC |
| 16112 | T(T) | 8 | 16119 | rpoC2 | CDS | LSC |
| 17829 | T(T) | 7 | 17835 | rpoC2 | CDS | LSC |
| 18097 | T(A) | 9 | 18105 | rpoC2 | CDS | LSC |
| 20394 | T(T) | 7 | 20400 | rpoC1 | CDS | LSC |
| 20585 | T(A) | 8 | 20592 | rpoC1 | CDS | LSC |
| 22240 | T(A) | 11 | 22250 | rpoC1 | intron | LSC |
| 25239 | T(A) | 7 | 25245 | rpoB | CDS | LSC |
| 26802 | T(A) | 7 | 26808 | rpoB-trnC(GCA) | IGS | LSC |
| 27665 | T(A) | 10 | 27674 | trnC(GCA)-petN | IGS | LSC |
| 27975 | T(T) | 8 | 27982 | trnC(GCA)-petN | IGS | LSC |
| 29365 | T(T) | 7 | 29371 | psbM-trnD(GUC) | IGS | LSC |
| 29492 | T(T) | 10 | 29501 | psbM-trnD(GUC) | IGS | LSC |
| 30640 | T(T) | 7 | 30646 | trnT(GGU)-psbD | IGS | LSC |
| 33209 | T(G) | 7 | 33215 | psbC | CDS | LSC |
| 33959 | T(G) | 7 | 33965 | psbC | CDS | LSC |
| 34429 | T(T) | 7 | 34435 | psbC-trnS(UGA) | IGS | LSC |
| 35637 | T(A) | 11 | 35647 | trnG(UCC)-trnfM(CAU) | IGS | LSC |
| 38775 | T(A) | 7 | 38781 | psaA | CDS | LSC |
| 39261 | T(C) | 7 | 39267 | psaA | CDS | LSC |
| 40001 | T(A) | 7 | 40007 | psaA | CDS | LSC |
| 41348 | T(T) | 7 | 41354 | psaA-ycf3 | IGS | LSC |
| 42072 | T(T) | 8 | 42079 | ycf3 | intron | LSC |
| 42397 | T(T) | 9 | 42405 | ycf3 | intron | LSC |
| 43471 | T(A) | 10 | 43480 | ycf3 | intron | LSC |
| 46025 | T(A) | 10 | 46034 | trnL(UAA) | intron | LSC |
| 47129 | T(T) | 7 | 47135 | ndhK(pseudo) | pseudo | LSC |
| 47344 | T(A) | 8 | 47351 | ndhK(pseudo) | pseudo | LSC |
| 47607 | T(A) | 9 | 47615 | ndhC(pseudo) | pseudo | LSC |
| 47785 | T(A) | 7 | 47791 | ndhC(pseudo)-trnV(UAC) | IGS | LSC |
| 49026 | T(T) | 10 | 49035 | trnM(CAU)-atpE | IGS | LSC |
| 51452 | T(T) | 9 | 51460 | atpB-rbcL | IGS | LSC |
| 53745 | T(A) | 7 | 53751 | rbcL-accD | IGS | LSC |
| 55708 | T(A) | 7 | 55714 | accD-psaI | IGS | LSC |
| 57143 | T(T) | 13 | 57155 | ycf4-cemA | IGS | LSC |
| 57327 | T(T) | 8 | 57334 | ycf4-cemA | IGS | LSC |
| 58403 | T(T) | 7 | 58409 | cemA-petA | IGS | LSC |
| 59006 | T(A) | 7 | 59012 | petA | CDS | LSC |
| 59477 | T(T) | 7 | 59483 | petA | CDS | LSC |
| 60030 | T(T) | 9 | 60038 | petA-psbJ | IGS | LSC |
| 60844 | T(A) | 8 | 60851 | psbF | CDS | LSC |
| 62407 | T(T) | 7 | 62413 | petG-trnW(CCA) | IGS | LSC |
| 62606 | T(A) | 7 | 62612 | trnW(CCA)-trnP(UGG) | IGS | LSC |
| 63176 | T(T) | 7 | 63182 | psaJ | CDS | LSC |
| 65508 | T(T) | 14 | 65521 | rpl20-rps12 | IGS | LSC |
| 65664 | T(T) | 7 | 65670 | rpl20-rps12 | IGS | LSC |
| 65972 | T(T) | 10 | 65981 | rps12-clpP | IGS | LSC |
| 66700 | T(T) | 7 | 66706 | clpP | intron | LSC |
| 66852 | T(A) | 10 | 66861 | clpP | intron | LSC |
| 67230 | T(A) | 7 | 67236 | clpP | CDS | LSC |
| 67343 | T(A) | 8 | 67350 | clpP | intron | LSC |
| 68316 | T(A) | 7 | 68322 | clpP-psbB | IGS | LSC |
| 69167 | T(T) | 8 | 69174 | psbB | CDS | LSC |
| 69516 | T(T) | 7 | 69522 | psbB | CDS | LSC |
| 70802 | T(T) | 9 | 70810 | psbH-petB | IGS | LSC |
| 71090 | T(A) | 8 | 71097 | psbH-petB | IGS | LSC |
| 71289 | T(A) | 11 | 71299 | petB | intron | LSC |
| 74833 | T(T) | 7 | 74839 | rps11 | CDS | LSC |
| 75297 | T(T) | 7 | 75303 | rps11-rpl36 | IGS | LSC |
| 75878 | T(A) | 7 | 75884 | infA-rps8 | IGS | LSC |
| 76229 | T(T) | 7 | 76235 | rps8 | CDS | LSC |
| 76925 | T(A) | 7 | 76931 | rpl14-rpl16 | IGS | LSC |
| 78884 | T(T) | 7 | 78890 | rps3 | CDS | LSC |
| 79174 | T(T) | 7 | 79180 | rpl22 | CDS | LSC |
| 80013 | T(T) | 7 | 80019 | rpl2 | CDS | IR |
| 83641 | T(A) | 8 | 83648 | ycf2 | CDS | IR |
| 84086 | T(A) | 7 | 84092 | ycf2 | CDS | IR |
| 84664 | T(T) | 7 | 84670 | ycf2 | CDS | IR |
| 84915 | T(T) | 8 | 84922 | ycf2 | CDS | IR |
| 85254 | T(A) | 9 | 85262 | ycf2 | CDS | IR |
| 87907 | T(A) | 7 | 87913 | ycf2 | CDS | IR |
| 90886 | T(A) | 8 | 90893 | ndhB(pseudo) | pseudo | IR |
| 91021 | T(A) | 11 | 91031 | ndhB(pseudo) | pseudo | IR |
| 94294 | T(T) | 8 | 94301 | rps12-trnV(GAC) | IGS | IR |
| 95346 | T(A) | 7 | 95352 | rps12-trnV(GAC) | IGS | IR |
| 97801 | T(G) | 7 | 97807 | trnI(GAU) | intron | IR |
| 98006 | T(T) | 8 | 98013 | trnI(GAU) | intron | IR |
| 99243 | T(G) | 7 | 99249 | trnA(UGC) | intron | IR |
| 99402 | T(G) | 7 | 99408 | trnA(UGC) | intron | IR |
| 101842 | T(G) | 7 | 101848 | 23SrRNA | rRNA | IR |
| 103258 | T(T) | 7 | 103264 | 5SrRNA-trnR(ACG) | IGS | IR |
| 103556 | T(T) | 7 | 103562 | trnR(ACG)-trnN(GUU) | IGS | IR |
| 107433 | T(T) | 7 | 107439 | ccsA-psaC | IGS | SSC |
| 108658 | T(A) | 7 | 108664 | ndhE(pseudo)-ndhI(pseudo) | IGS | SSC |
| 109744 | T(T) | 7 | 109750 | ndhI(pseudo)-rps15 | IGS | SSC |
| 109951 | T(T) | 9 | 109959 | rps15 | CDS | SSC |
| 111145 | T(T) | 8 | 111152 | ycf1 | CDS | SSC |
| 111653 | T(T) | 7 | 111659 | ycf1 | CDS | SSC |
| 111888 | T(A) | 7 | 111894 | ycf1 | CDS | SSC |
| 112092 | T(A) | 11 | 112102 | ycf1 | CDS | SSC |
| 112983 | T(T) | 13 | 112995 | ycf1 | CDS | SSC |
| 113598 | T(A) | 7 | 113604 | ycf1 | CDS | SSC |
| 115123 | T(T) | 9 | 115131 | ycf1 | CDS | SSC |
| 116502 | T(A) | 7 | 116508 | trnN(GUU)-trnR(ACG) | IGS | IR |
| 116800 | T(A) | 7 | 116806 | trnR(ACG)-5SrRNA | IGS | IR |
| 118216 | T(C) | 7 | 118222 | 23SrRNA | CDS | IR |
| 120656 | T(C) | 7 | 120662 | trnA(UGC) | intron | IR |
| 120815 | T(C) | 7 | 120821 | trnA(UGC) | intron | IR |
| 122051 | T(A) | 8 | 122058 | trnI(GAU) | intron | IR |
| 122257 | T(C) | 7 | 122263 | trnI(GAU) | intron | IR |
| 124712 | T(T) | 7 | 124718 | trnV(GAC)-rps12 | IGS | IR |
| 125763 | T(A) | 8 | 125770 | trnV(GAC)-rps12 | IGS | IR |
| 129033 | T(T) | 11 | 129043 | ndhB(pseudo) | pseudo | IR |
| 129171 | T(T) | 8 | 129178 | ndhB(pseudo) | pseudo | IR |
| 132151 | T(T) | 7 | 132157 | ycf2 | CDS | IR |
| 134802 | T(T) | 9 | 134810 | ycf2 | CDS | IR |
| 135142 | T(A) | 8 | 135149 | ycf2 | CDS | IR |
| 135394 | T(A) | 7 | 135400 | ycf2 | CDS | IR |
| 135972 | T(T) | 7 | 135978 | ycf2 | CDS | IR |
| 136416 | T(T) | 8 | 136423 | ycf2 | CDS | IR |
| 140045 | T(A) | 7 | 140051 | rpl2 | CDS | IR |
| 18839 | T(CG) | 8 | 18846 | rpoC2 | CDS | LSC |
| 21912 | T(TA) | 8 | 21919 | rpoC1 | intron | LSC |
| 34538 | T(GA) | 8 | 34545 | trnS(UGA) | CDS | LSC |
| 40160 | T(AG) | 8 | 40167 | psaA | CDS | LSC |
| 52370 | T(GA) | 8 | 52377 | rbcL | CDS | LSC |
| 53596 | T(TA) | 8 | 53603 | rbcL-accD | IGS | LSC |
| 57739 | T(TC) | 8 | 57746 | cemA | CDS | LSC |
| 58645 | T(AT) | 8 | 58652 | petA | CDS | LSC |
| 80464 | T(TA) | 8 | 80471 | rpl2 | intron | IR |
| 80754 | T(TC) | 8 | 80761 | rpl2 | intron | IR |
| 88589 | T(TA) | 8 | 88596 | ycf2 | CDS | IR |
| 90441 | T(AG) | 8 | 90448 | ndhB(pseudo) | pseudo | IR |
| 101373 | T(CT) | 8 | 101380 | 23SrRNA | rRNA | IR |
| 118684 | T(AG) | 8 | 118691 | 23SrRNA | rRNA | IR |
| 129616 | T(CT) | 8 | 129623 | ndhB(pseudo) | pseudo | IR |
| 131468 | T(TA) | 8 | 131475 | ycf2 | CDS | IR |
| 139302 | T(AG) | 8 | 139309 | rpl2 | intron | IR |
| 139592 | T(AT) | 8 | 139599 | rpl2 | intron | IR |
| 34208 | T(TTC) | 12 | 34219 | psbC | CDS | LSC |
| 5064 | T(GATA) | 12 | 5075 | trnK(UUU)-rps16 | IGS | LSC |

| **Sequence repeats in *Genlisea repens.* Type, length, region, location and plastome quadripartite region. (F = Forward repeats; P= Palindromic repeats; T = Tandem repeats)** | | | | | | |
| --- | --- | --- | --- | --- | --- | --- |
| Repeat Start 1 | Type | Lenght (bp) | Repeat Start 2 | Gene | Location | Region |
| 37650 | F | 40 | 39874 | psaB;psaA | CDS | LSC |
| 48723 | F | 31 | 48747 | trnV(UAC)-trnM(CAU) | IGS | LSC |
| 8079 | F | 30 | 34513 | psbI-trnS(GCU);psbC-trnS(UGA) | IGS | LSC |
| 9639 | F | 30 | 35446 | trnC(UCC);trnG(UCC) | tRNA | LSC |
| 63354 | P | 69 | 63354 | psaJ-rpl33 | IGS | LSC |
| 29084 | P | 38 | 29084 | psbM-trnD(GUC) | IGS | LSC |
| 41119 | P | 44 | 41119 | psaA-ycf3 | IGS | LSC |
| 56449 | P | 36 | 56449 | psaI-ycf4 | IGS | LSC |
| 8079 | P | 30 | 44230 | psbI-trnS(GCU);trnG(UCC) | IGS;tRNA | LSC |
| 109689 | P | 30 | 109689 | ndhE(pseudo)-rps15 | IGS | LSC |
| 34513 | P | 30 | 44230 | psbC-trnS(UGA);trnS(GGA) | intron;tRNA | LSC |
| 1528 | T(A) | 10 | 1537 | psbA-trnK(UUU) | IGS | LSC |
| 2285 | T(T) | 10 | 2294 | matK | CDS | LSC |
| 3873 | T(G) | 7 | 3879 | trnK(UUU) | intron | LSC |
| 4389 | T(A) | 7 | 4395 | trnK(UUU)-rps16 | IGS | LSC |
| 7420 | T(T) | 7 | 7426 | psbK | CDS | LSC |
| 7825 | T(T) | 11 | 7835 | psbK-psbI | IGS | LSC |
| 8794 | T(A) | 7 | 8800 | trnS(GCU)-trnG(UCC) | IGS | LSC |
| 12673 | T(A) | 8 | 12680 | atpF | CDS | LSC |
| 13091 | T(A) | 8 | 13098 | atpF-atpH | IGS | LSC |
| 13529 | T(T) | 7 | 13535 | atpH-atpI | IGS | LSC |
| 13824 | T(T) | 7 | 13830 | atpH-atpI | IGS | LSC |
| 16123 | T(T) | 8 | 16130 | rpoC2 | CDS | LSC |
| 17840 | T(T) | 7 | 17846 | rpoC2 | CDS | LSC |
| 18108 | T(A) | 9 | 18116 | rpoC2 | CDS | LSC |
| 20405 | T(T) | 7 | 20411 | rpoC1 | CDS | LSC |
| 20596 | T(A) | 8 | 20603 | rpoC1 | CDS | LSC |
| 22238 | T(A) | 13 | 22250 | rpoC1 | intron | LSC |
| 25240 | T(A) | 7 | 25246 | rpoB | CDS | LSC |
| 26805 | T(A) | 7 | 26811 | rpoB-trnC(GCA) | IGS | LSC |
| 27947 | T(T) | 9 | 27955 | trnC(GCA)-petN | IGS | LSC |
| 29331 | T(T) | 7 | 29337 | psbM-trnD(GUC) | IGS | LSC |
| 29463 | T(T) | 9 | 29471 | psbM-trnD(GUC) | IGS | LSC |
| 30632 | T(T) | 7 | 30638 | trnT(GGU)-psbD | IGS | LSC |
| 31626 | T(T) | 7 | 31632 | trnT(GGU)-psbD | IGS | LSC |
| 33198 | T(G) | 7 | 33204 | psbC | CDS | LSC |
| 33948 | T(G) | 7 | 33954 | psbC | CDS | LSC |
| 34408 | T(T) | 7 | 34414 | psbC-trnS(UGA) | IGS | LSC |
| 38759 | T(A) | 7 | 38765 | psaA | CDS | LSC |
| 39245 | T(C) | 7 | 39251 | psaA | CDS | LSC |
| 39985 | T(A) | 7 | 39991 | psaA | CDS | LSC |
| 41340 | T(T) | 7 | 41346 | psaA-ycf3 | IGS | LSC |
| 42028 | T(T) | 10 | 42037 | ycf3 | intron | LSC |
| 42354 | T(T) | 11 | 42364 | ycf3 | intron | LSC |
| 46020 | T(A) | 11 | 46030 | trnL(UAA) | intron | LSC |
| 46656 | T(T) | 11 | 46666 | trnL(UAA)-trnF(GAA) | IGS | LSC |
| 47176 | T(T) | 7 | 47182 | ndhK(pseudo) | pseudo | LSC |
| 47669 | T(A) | 8 | 47676 | ndhC(pseudo) | pseudo | LSC |
| 47828 | T(A) | 7 | 47834 | ndhC(pseudo)-trnV(UAC) | IGS | LSC |
| 49074 | T(T) | 13 | 49086 | trnM(CAU)-atpE | IGS | LSC |
| 51502 | T(T) | 11 | 51512 | atpB-rbcL | IGS | LSC |
| 58454 | T(T) | 7 | 58460 | cemA-petA | IGS | LSC |
| 59057 | T(A) | 7 | 59063 | petA | CDS | LSC |
| 59528 | T(T) | 7 | 59534 | petA | CDS | LSC |
| 60106 | T(T) | 11 | 60116 | petA-psbJ | IGS | LSC |
| 60262 | T(A) | 9 | 60270 | petA-psbJ | IGS | LSC |
| 60925 | T(A) | 8 | 60932 | psbF | CDS | LSC |
| 62334 | T(A) | 11 | 62344 | petL-petG | IGS | LSC |
| 62488 | T(T) | 7 | 62494 | petG-trnW(CCA) | IGS | LSC |
| 62687 | T(A) | 7 | 62693 | trnW(CCA)-trnP(UGG) | IGS | LSC |
| 63257 | T(T) | 7 | 63263 | psaJ | CDS | LSC |
| 65616 | T(T) | 10 | 65625 | rpl20-rps12 | IGS | LSC |
| 65768 | T(T) | 7 | 65774 | rpl20-rps12 | IGS | LSC |
| 66975 | T(A) | 10 | 66984 | clpP | intron | LSC |
| 67353 | T(A) | 7 | 67359 | clpP | CDS | LSC |
| 67466 | T(A) | 8 | 67473 | clpP | intron | LSC |
| 69297 | T(T) | 8 | 69304 | psbB | CDS | LSC |
| 69646 | T(T) | 7 | 69652 | psbB | CDS | LSC |
| 70100 | T(T) | 9 | 70108 | psbB-psbT | IGS | LSC |
| 70297 | T(T) | 7 | 70303 | psbT | CDs | LSC |
| 71207 | T(A) | 7 | 71213 | petB | intron | LSC |
| 71405 | T(A) | 10 | 71414 | petB | intron | LSC |
| 74962 | T(T) | 7 | 74968 | rps11 | CDS | LSC |
| 75426 | T(T) | 7 | 75432 | rps11-rpl36 | IGS | LSC |
| 76007 | T(A) | 7 | 76013 | infA-rps8 | IGS | LSC |
| 76358 | T(T) | 7 | 76364 | rps8 | CDS | LSC |
| 77050 | T(A) | 7 | 77056 | rpl14-rpl16 | IGS | LSC |
| 77594 | T(A) | 7 | 77600 | rpl16 | intron | LSC |
| 77742 | T(T) | 9 | 77750 | rpl16 | intron | LSC |
| 78999 | T(T) | 7 | 79005 | rps3 | CDS | LSC |
| 79293 | T(T) | 7 | 79299 | rpl22 | CDS | LSC |
| 80132 | T(T) | 7 | 80138 | rpl2 | CDS | IR |
| 83766 | T(A) | 8 | 83773 | ycf2 | CDS | IR |
| 84211 | T(A) | 7 | 84217 | ycf2 | CDS | IR |
| 84789 | T(T) | 7 | 84795 | ycf2 | CDS | IR |
| 85379 | T(A) | 9 | 85387 | ycf2 | CDS | IR |
| 88032 | T(A) | 7 | 88038 | ycf2 | CDS | IR |
| 91011 | T(A) | 8 | 91018 | ndhB(pseudo) | pseudo | IR |
| 91146 | T(A) | 11 | 91156 | ndhB(pseudo) | pseudo | IR |
| 94412 | T(T) | 8 | 94419 | rps12-trnV(GAC) | IGS | IR |
| 95464 | T(A) | 7 | 95470 | rps12-trnV(GAC) | IGS | IR |
| 97919 | T(G) | 7 | 97925 | trnI(GAU) | intron | IR |
| 98124 | T(T) | 8 | 98131 | trnI(GAU) | intron | IR |
| 99361 | T(G) | 7 | 99367 | trnA(UGC) | intron | IR |
| 99520 | T(G) | 7 | 99526 | trnA(UGC) | intron | IR |
| 101960 | T(G) | 7 | 101966 | 23SrRNA | rRNA | IR |
| 103375 | T(T) | 7 | 103381 | 5SrRNA-trnR(ACG) | IGS | IR |
| 103673 | T(T) | 7 | 103679 | trnR(ACG)-trnN(GUU) | IGS | IR |
| 105774 | T(A) | 17 | 105790 | rpl32-trnL(UAG) | IGS | SSC |
| 107152 | T(A) | 7 | 107158 | ccsA-psaC | IGS | SSC |
| 107391 | T(T) | 7 | 107397 | ccsA-psaC | IGS | SSC |
| 109888 | T(T) | 7 | 109894 | ndhE(pseudo)-rps15 | IGS | SSC |
| 110095 | T(T) | 9 | 110103 | rps15 | CDS | SSC |
| 111272 | T(T) | 8 | 111279 | ycf1 | CDS | SSC |
| 111610 | T(T) | 7 | 111616 | ycf1 | CDS | SSC |
| 111780 | T(T) | 7 | 111786 | ycf1 | CDS | SSC |
| 112015 | T(A) | 7 | 112021 | ycf1 | CDS | SSC |
| 112201 | T(A) | 11 | 112211 | ycf1 | CDS | SSC |
| 112652 | T(T) | 7 | 112658 | ycf1 | CDS | SSC |
| 112862 | T(T) | 7 | 112868 | ycf1 | CDS | SSC |
| 113092 | T(T) | 14 | 113105 | ycf1 | CDS | SSC |
| 113707 | T(A) | 7 | 113713 | ycf1 | CDS | SSC |
| 114540 | T(T) | 7 | 114546 | ycf1 | CDS | SSC |
| 114699 | T(A) | 7 | 114705 | ycf1 | CDS | IR |
| 116629 | T(A) | 7 | 116635 | trnN(GUU)-trnR(ACG) | IGS | IR |
| 116927 | T(A) | 7 | 116933 | trnR(ACG)-5SrRNA | IGS | IR |
| 118342 | T(C) | 7 | 118348 | 23SrRNA | rRNA | IR |
| 120782 | T(C) | 7 | 120788 | trnA(UGC) | intron | IR |
| 120941 | T(C) | 7 | 120947 | trnA(UGC) | intron | IR |
| 122177 | T(A) | 8 | 122184 | trnI(GAU) | intron | IR |
| 122383 | T(C) | 7 | 122389 | trnI(GAU) | intron | IR |
| 124838 | T(T) | 7 | 124844 | trnV(GAC)-rps12 | IGS | IR |
| 125889 | T(A) | 8 | 125896 | trnV(GAC)-rps12 | IGS | IR |
| 129152 | T(T) | 11 | 129162 | ndhB(pseudo) | pseudo | IR |
| 129290 | T(T) | 8 | 129297 | ndhB(pseudo) | pseudo | IR |
| 132270 | T(T) | 7 | 132276 | ycf2 | CDS | IR |
| 134921 | T(T) | 9 | 134929 | ycf2 | CDS | IR |
| 135513 | T(A) | 7 | 135519 | ycf2 | CDS | IR |
| 136091 | T(T) | 7 | 136097 | ycf2 | CDS | IR |
| 136535 | T(T) | 8 | 136542 | ycf2 | CDS | IR |
| 140170 | T(A) | 7 | 140176 | rpl2 | CDS | IR |
| 3705 | T(AT) | 8 | 3712 | trnK(UUU) | intron | LSC |
| 10186 | T(TA) | 8 | 10193 | atpA | CDS | LSC |
| 18850 | T(CG) | 8 | 18857 | rpoC2 | CDS | LSC |
| 34517 | T(GA) | 8 | 34524 | trnS(UGA) | tRNA | LSC |
| 40144 | T(AG) | 8 | 40151 | psaA | CDS | LSC |
| 52424 | T(GA) | 8 | 52431 | rbcL | CDS | LSC |
| 53644 | T(TA) | 8 | 53651 | rbcL-accD | IGS | LSC |
| 57790 | T(TC) | 8 | 57797 | cemA | CDS | LSC |
| 58696 | T(AT) | 8 | 58703 | petA | CDS | LSC |
| 80583 | T(TA) | 8 | 80590 | rpl2 | intron | IR |
| 80873 | T(TC) | 8 | 80880 | rpl2 | intron | IR |
| 88714 | T(TA) | 8 | 88721 | ycf2 | CDS | IR |
| 90566 | T(AG) | 8 | 90573 | ndhB(pseudo) | pseudo | IR |
| 101491 | T(CT) | 8 | 101498 | 23SrRNA | rRNA | IR |
| 110632 | T(TA) | 8 | 110639 | rps15-ycf1 | IGS | SSC |
| 118810 | T(AG) | 8 | 118817 | 23SrRNA | rRNA | IR |
| 129735 | T(CT) | 8 | 129742 | ndhB(pseudo) | pseudo | IR |
| 131587 | T(TA) | 8 | 131594 | ycf2 | CDS | IR |
| 139427 | T(AG) | 8 | 139434 | rpl2 | intron | IR |
| 139717 | T(AT) | 8 | 139724 | rpl2 | intron | IR |
| 34197 | T(TTC) | 12 | 34208 | psbC | CDS | LSC |
| 1647 | T(CTAA) | 12 | 1658 | psbA-trnK(UUU) | IGS | LSC |
| 9974 | T(ATTT) | 12 | 9985 | trnR(UCU)-atpA | IGS | LSC |
| 104995 | T(ACAAT) | 15 | 105009 | ycf1 | CDS | SSC |

| **Sequence repeats in *Genlisea violacea.* Type, length, region, location and plastome quadripartite region. (F = Forward repeats; P= Palindromic repeats; T = Tandem repeats)** | | | | | | |
| --- | --- | --- | --- | --- | --- | --- |
| Repeat Start 1 | Type | Lenght (bp) | Repeat Start 2 | Region | Location | Plastome region |
| 89076 | F | 48 | 89094 | ycf2 | CDS | IR |
| 135354 | F | 48 | 135372 | ycf2 | CDS | IR |
| 89064 | F | 47 | 89082 | ycf2 | CDS | IR |
| 135367 | F | 47 | 135385 | ycf2 | CDS | IR |
| 89084 | F | 44 | 89102 | ycf2 | CDS | IR |
| 38243 | F | 41 | 40467 | psaB;psaA | CDS | LSC |
| 89066 | F | 40 | 89102 | ycf2 | CDS | IR |
| 135354 | F | 40 | 135390 | ycf2 | CDS | IR |
| 135377 | F | 37 | 135395 | ycf2 | CDS | IR |
| 44941 | F | 30 | 44967 | trnS(GGA)-rps4 | IGS | LSC |
| 89076 | F | 34 | 89112 | ycf2 | CDS | IR |
| 81486 | F | 31 | 81546 | rpl22; rpl22-rps19 | CDS;IGS | IR |
| 142919 | F | 31 | 142979 | rps19-rpl22 | IGS | IR |
| 7922 | F | 32 | 35078 | psbI-trnS(GCU);psbC-trnS(UGA) | IGS | LSC |
| 89064 | F | 31 | 89118 | ycf2 | CDS | IR |
| 135350 | F | 31 | 135404 | ycf2 | CDS | IR |
| 9451 | F | 30 | 36023 | trnG(UCC);trnG(UCC) | tRNA | LSC |
| 49521 | F | 30 | 49533 | ndhC(pseudo)-trnV(UAC) | IGS | IR |
| 86676 | F | 30 | 86718 | ycf2 | CDS | IR |
| 135367 | F | 30 | 135403 | ycf2 | CDS | IR |
| 137748 | F | 30 | 137790 | ycf2 | CDS | IR |
| 108159 | P | 48 | 108159 | rpl32-trnL(UAG) | IGS | SSC |
| 89076 | P | 48 | 135354 | ycf2;ycf2 | CDS | IR |
| 89094 | P | 48 | 135372 | ycf2;ycf2 | CDS | IR |
| 89064 | P | 47 | 135367 | ycf2;ycf2 | CDS | IR |
| 89082 | P | 47 | 135385 | ycf2;ycf2 | CDS | IR |
| 111000 | P | 36 | 111000 | ndhD(pseudo)-psaC | IGS | LSC |
| 41736 | P | 39 | 41736 | psaA-ycf3 | IGS | LSC |
| 89084 | P | 44 | 135350 | ycf2;ycf2 | CDS | IR |
| 89102 | P | 44 | 135368 | ycf2;ycf2 | CDS | IR |
| 74616 | P | 40 | 74616 | petD | intron | LSC |
| 29617 | P | 32 | 29617 | psbM-trnD(GUC) | IGS | LSC |
| 89066 | P | 40 | 135354 | ycf2;ycf2 | CDS | IR |
| 89102 | P | 40 | 135390 | ycf2;ycf2 | CDS | IR |
| 7924 | P | 30 | 44737 | psbI-trnS(GCU);trnS(GGA) | IGS;tRNA | LSC |
| 114976 | P | 33 | 114976 | ycf1 | CDS | SSC |
| 89076 | P | 34 | 135350 | ycf2;ycf2 | CDS | IR |
| 89112 | P | 34 | 135386 | ycf2;ycf2 | CDS | IR |
| 81486 | P | 31 | 142919 | rpl22;rps19-rpl22 | CDS;IGS | IR |
| 81546 | P | 31 | 142979 | rpl22-rps19;rps19-rpl22 | IGS | IR |
| 62209 | P | 30 | 62248 | petA-psbJ | IGS | LSC |
| 63643 | P | 30 | 63679 | psbE-petL | IGS | LSC |
| 89064 | P | 31 | 135347 | ycf2;ycf2 | CDS | IR |
| 89118 | P | 31 | 135401 | ycf2;ycf2 | CDS | IR |
| 35080 | P | 30 | 44737 | psbC-trnS(UGA);trnS(GGA) | IGS;tRNA | LSC |
| 86676 | P | 30 | 137748 | ycf2;ycf2 | CDS | IR |
| 86718 | P | 30 | 137790 | ycf2;ycf2 | CDS | IR |
| 1619 | T(A) | 9 | 1627 | psbA-trnK(UUU) | IGS | LSC |
| 1948 | T(A) | 9 | 1956 | trnK(UUU) | intron | LSC |
| 2216 | T(T) | 10 | 2225 | matK | CDS | LSC |
| 2872 | T(A) | 8 | 2879 | matK | CDS | LSC |
| 3664 | T(T) | 8 | 3671 | trnK(UUU) | intron | LSC |
| 4695 | T(T) | 7 | 4701 | trnK(UUU)-rps16 | IGS | LSC |
| 5347 | T(C) | 7 | 5353 | rps16 | intron | LSC |
| 7298 | T(T) | 7 | 7304 | psbK | CDS | LSC |
| 7670 | T(T) | 10 | 7679 | psbK-psbI | IGS | LSC |
| 8523 | T(T) | 7 | 8529 | trnS(GCU)-trnG(UCC) | IGS | LSC |
| 8850 | T(A) | 7 | 8856 | trnG(UCC) | intron | LSC |
| 11367 | T(A) | 8 | 11374 | atpA-atpF | IGS | LSC |
| 12082 | T(T) | 9 | 12090 | atpF | intron | LSC |
| 13372 | T(T) | 7 | 13378 | atpH-atpI | IGS | LSC |
| 13686 | T(T) | 7 | 13692 | atpH-atpI | IGS | LSC |
| 16202 | T(T) | 9 | 16210 | rpoC2 | CDS | LSC |
| 17666 | T(T) | 7 | 17672 | rpoC2 | CDS | LSC |
| 18062 | T(T) | 11 | 18072 | rpoC2 | CDS | LSC |
| 18205 | T(A) | 8 | 18212 | rpoC2 | CDS | LSC |
| 20485 | T(T) | 7 | 20491 | rpoC1 | CDS | LSC |
| 20676 | T(A) | 7 | 20682 | rpoC1 | CDS | LSC |
| 22099 | T(A) | 7 | 22105 | rpoC1 | intron | LSC |
| 22258 | T(A) | 7 | 22264 | rpoC1 | intron | LSC |
| 25285 | T(A) | 7 | 25291 | rpoB | CDS | LSC |
| 25769 | T(A) | 7 | 25775 | rpoB | CDS | LSC |
| 26393 | T(A) | 8 | 26400 | rpoB | CDS | LSC |
| 26854 | T(A) | 8 | 26861 | rpoB-trnC(GCA) | IGS | LSC |
| 27394 | T(T) | 9 | 27402 | rpoB-trnC(GCA) | IGS | LSC |
| 27752 | T(A) | 7 | 27758 | trnC(GCA)-petN | IGS | LSC |
| 28753 | T(T) | 10 | 28762 | petN-psbM | IGS | LSC |
| 29779 | T(A) | 10 | 29788 | psbM-trnD(GUC) | IGS | LSC |
| 29941 | T(A) | 7 | 29947 | psbM-trnD(GUC) | IGS | LSC |
| 31120 | T(T) | 7 | 31126 | trnT(GGU)-psbD | IGS | LSC |
| 31461 | T(C) | 7 | 31467 | trnT(GGU)-psbD | IGS | LSC |
| 33735 | T(G) | 7 | 33741 | psbC | CDs | LSC |
| 35322 | T(A) | 8 | 35329 | trnS(UGA)-psbZ | IGS | LSC |
| 36667 | T(T) | 7 | 36673 | rps14 | CDS | LSC |
| 39353 | T(A) | 7 | 39359 | psaA | CDS | LSC |
| 39839 | T(C) | 7 | 39845 | psaA | CDS | LSC |
| 40939 | T(A) | 7 | 40945 | psaA | CDS | LSC |
| 42855 | T(T) | 8 | 42862 | ycf3 | intron | LSC |
| 42989 | T(T) | 7 | 42995 | ycf3 | intron | LSC |
| 46485 | T(A) | 9 | 46493 | trnL(UAA) | intron | LSC |
| 47083 | T(T) | 13 | 47095 | trnL(UAA)-trnF(GAA) | IGS | LSC |
| 47312 | T(T) | 11 | 47322 | trnF(GAA)-ndhJ(pseudo) | IGS | LSC |
| 47680 | T(T) | 7 | 47686 | trnF(GAA)-ndhJ(pseudo) | IGS | LSC |
| 50140 | T(T) | 7 | 50146 | trnV(UAC) | intron | LSC |
| 50578 | T(T) | 8 | 50585 | trnM(CAU)-atpE | IGS | LSC |
| 50706 | T(T) | 8 | 50713 | trnM(CAU)-atpE | IGS | LSC |
| 53006 | T(T) | 11 | 53016 | atpB-rbcL | IGS | LSC |
| 55747 | T(G) | 7 | 55753 | accD | CDS | LSC |
| 55919 | T(T) | 7 | 55925 | accD | CDS | LSC |
| 56666 | T(G) | 8 | 56673 | accD | CDS | LSC |
| 57989 | T(A) | 7 | 57995 | psaI-ycf4 | IGS | LSC |
| 58881 | T(T) | 11 | 58891 | ycf4-cemA | IGS | LSC |
| 60818 | T(A) | 7 | 60824 | petA | CDS | LSC |
| 61289 | T(T) | 7 | 61295 | petA | CDS | LSC |
| 61586 | T(T) | 7 | 61592 | petA-psbJ | IGS | LSC |
| 61892 | T(A) | 9 | 61900 | petA-psbJ | IGS | LSC |
| 62736 | T(A) | 8 | 62743 | psbF | CDS | LSC |
| 64211 | T(T) | 7 | 64217 | petL-petG | IGS | LSC |
| 64375 | T(T) | 10 | 64384 | petG-trnW(CCA) | IGS | LSC |
| 64596 | T(T) | 8 | 64603 | trnW(CCA)-trnP(UGG) | IGS | LSC |
| 65201 | T(T) | 7 | 65207 | psaJ | CDS | LSC |
| 65529 | T(T) | 8 | 65536 | psaJ-rpl33 | IGS | LSC |
| 66447 | T(T) | 8 | 66454 | rps18-rpl20 | IGS | LSC |
| 67066 | T(T) | 7 | 67072 | rpl20-rps12 | IGS | LSC |
| 67611 | T(T) | 10 | 67620 | rpl20-rps12 | IGS | LSC |
| 68002 | T(T) | 9 | 68010 | rps12-clpP | IGS | LSC |
| 68533 | T(T) | 10 | 68542 | clpP | intron | LSC |
| 69144 | T(A) | 7 | 69150 | clpP | CDS | LSC |
| 69680 | T(T) | 8 | 69687 | clpP | intron | LSC |
| 71131 | T(T) | 8 | 71138 | psbB | CDS | LSC |
| 71480 | T(T) | 7 | 71486 | psbB | CDS | LSC |
| 73487 | T(T) | 9 | 73495 | petB | intron | LSC |
| 73677 | T(G) | 7 | 73683 | petB | CDS | LSC |
| 75024 | T(T) | 7 | 75030 | petD | intron | LSC |
| 75707 | T(T) | 7 | 75713 | petD-rpoA | IGS | LSC |
| 76851 | T(T) | 7 | 76857 | rps11 | CDS | LSC |
| 77321 | T(T) | 7 | 77327 | rps11-rpl36 | IGS | LSC |
| 78245 | T(T) | 7 | 78251 | rps8 | CDS | LSC |
| 78362 | T(T) | 7 | 78368 | rps8-rpl14 | IGS | LSC |
| 78480 | T(T) | 7 | 78486 | rps8-rpl14 | IGS | LSC |
| 78925 | T(A) | 10 | 78934 | rpl14-rpl16 | IGS | LSC |
| 79589 | T(A) | 9 | 79597 | rpl16 | intron | LSC |
| 80905 | T(T) | 9 | 80913 | rps3 | CDS | LSC |
| 82041 | T(T) | 7 | 82047 | rpl2 | CDS | IR |
| 86122 | T(A) | 7 | 86128 | ycf2 | CDS | IR |
| 86700 | T(T) | 7 | 86706 | ycf2 | CDS | IR |
| 89976 | T(A) | 7 | 89982 | ycf2 | CDS | IR |
| 93094 | T(A) | 13 | 93106 | ndhB(pseudo) | pseudo | IR |
| 96374 | T(T) | 8 | 96381 | rps12-trnV(GAC) | IGS | IR |
| 99881 | T(G) | 7 | 99887 | trnI(GAU) | intron | IR |
| 100086 | T(T) | 8 | 100093 | trnI(GAU) | intron | IR |
| 101477 | T(G) | 7 | 101483 | trnA(UGC) | intron | IR |
| 103916 | T(G) | 7 | 103922 | 23SrRNA | rRNA | IR |
| 105160 | T(A) | 7 | 105166 | 5SrRNA-trnR(ACG) | IGS | IR |
| 105315 | T(T) | 7 | 105321 | 5SrRNA-trnR(ACG) | IGS | IR |
| 105693 | T(A) | 8 | 105700 | trnR(ACG)-trnN(GUU) | IGS | SSC |
| 107772 | T(T) | 8 | 107779 | rpl32 | CDS | SSC |
| 108382 | T(A) | 7 | 108388 | trnL(UAG)-ccsA | IGS | SSC |
| 108723 | T(A) | 8 | 108730 | ccsA | CDS | SSC |
| 109003 | T(T) | 9 | 109011 | ccsA | CDS | SSC |
| 109946 | T(A) | 7 | 109952 | ccsA-ndhD(pseudo) | IGS | SSC |
| 110079 | T(A) | 7 | 110085 | ccsA-ndhD(pseudo) | IGS | SSC |
| 110205 | T(T) | 9 | 110213 | trnL(UAG)-ndhD(pseudo) | IGS | SSC |
| 111043 | T(A) | 8 | 111050 | ndhD(pseudo)-psaC | IGS | SSC |
| 111851 | T(A) | 7 | 111857 | ndhE(pseudo) | pseudo | SSC |
| 112153 | T(A) | 9 | 112161 | ndhE(pseudo)-rps15 | IGS | SSC |
| 112270 | T(T) | 10 | 112279 | rps15 | CDS | SSC |
| 112851 | T(T) | 10 | 112860 | rps15-ycf1 | IGS | SSC |
| 113161 | T(A) | 7 | 113167 | ycf1 | CDS | SSC |
| 113728 | T(T) | 10 | 113737 | ycf1 | CDS | SSC |
| 114561 | T(T) | 7 | 114567 | ycf1 | CDS | SSC |
| 119176 | T(A) | 7 | 119182 | trnR(ACG)-5SrRNA | IGS | IR |
| 119331 | T(T) | 7 | 119337 | trnR(ACG)-5SrRNA | IGS | IR |
| 120575 | T(C) | 7 | 120581 | 23SrRNA | rRNA | IR |
| 123014 | T(C) | 7 | 123020 | trnA(UGC) | intron | IR |
| 124404 | T(A) | 8 | 124411 | trnI(GAU) | intron | IR |
| 124610 | T(C) | 7 | 124616 | trnI(GAU) | intron | IR |
| 128116 | T(A) | 8 | 128123 | trnV(GAC)-rps12 | IGS | IR |
| 131391 | T(T) | 13 | 131403 | ndhB(pseudo) | pseudo | IR |
| 134515 | T(T) | 7 | 134521 | ycf2 | CDS | IR |
| 137791 | T(A) | 7 | 137797 | ycf2 | CDS | IR |
| 138369 | T(T) | 7 | 138375 | ycf2 | CDS | IR |
| 142450 | T(A) | 7 | 142456 | rpl2 | CDS | IR |
| 6296 | T(TA) | 8 | 6303 | rps16-trnQ(UUG) | IGS | LSC |
| 19450 | T(AT) | 10 | 19459 | rpoC2 | CDS | LSC |
| 30207 | T(TA) | 8 | 30214 | trnD(GUC)-trnYT(GUA) | IGS | LSC |
| 40738 | T(AG) | 8 | 40745 | psaA | CDS | LSC |
| 42096 | T(AT) | 8 | 42103 | psaA-ycf3 | IGS | LSC |
| 46122 | T(AT) | 8 | 46129 | trnT(UGU)-trnL(UAA) | IGS | LSC |
| 53909 | T(GA) | 8 | 53916 | rbcL | CDS | LSC |
| 60457 | T(AT) | 8 | 60464 | petA | CDS | LSC |
| 82492 | T(TA) | 8 | 82499 | rpl2 | intron | IR |
| 82782 | T(TC) | 8 | 82789 | rpl2 | intron | IR |
| 88112 | T(CA) | 8 | 88119 | ycf2 | CDS | IR |
| 90658 | T(TA) | 8 | 90665 | ycf2 | CDS | IR |
| 92530 | T(AG) | 8 | 92537 | ndhB(pseudo) | pseudo | IR |
| 103447 | T(CT) | 8 | 103454 | 23SrRNA | rRNA | IR |
| 107267 | T(AT) | 8 | 107274 | ycf1-rpl32 | IGS | SSC |
| 121043 | T(AG) | 8 | 121050 | 23SrRNA | rRNA | IR |
| 131960 | T(CT) | 8 | 131967 | ndhB(pseudo) | pseudo | IR |
| 133832 | T(TA) | 8 | 133839 | ycf2 | CDS | IR |
| 136378 | T(TG) | 8 | 136385 | ycf2 | CDS | IR |
| 141707 | T(AG) | 8 | 141714 | rpl2 | intron | IR |
| 141997 | T(AT) | 8 | 142004 | rpl2 | intron | IR |
| 32690 | T(TTA) | 12 | 32701 | psbD | CDS | LSC |
| 34734 | T(TTC) | 12 | 34745 | psbC | CDS | LSC |
