# Supplementary material for: Comparative genomic analysis of Genlisea (corkscrew plants—Lentibulariaceae) chloroplast genomes reveals an increasing loss of the ndh genes
Source: PLoS One. 2018 Jan 2;13(1):e0190321. doi: 10.1371/journal.pone.0190321 (PMC5749785; doi:10.1371/journal.pone.0190321)
Supplement: S6 Table — Deleted ndh genes in all Genlisea species and boundaries between ndh pseudogenes are uncertain and were not included in this analysis (represented as n/a). (DOCX) [file pone.0190321.s011.docx]

**S6 Table. Phylogenetically informative characters (PIC) and p-distance of each gene for *Genlisea* species.** Deleted *ndh* genes in all *Genlisea* species and boundaries between *ndh* pseudogenes are uncertain and were not included in this analysis (represented as n/a).

| Region | PIC | p-distance |
| --- | --- | --- |
| ycf1 | 323 | 0.085 |
| rpl20-rps12 | 156 | 0.049 |
| rpoC2 | 107 | 0.035 |
| trnK-intron | 96 | 0.059 |
| rps12-trnV | 78 | 0.008 |
| matK | 70 | 0.061 |
| rpl16-intron | 57 | 0.078 |
| trnK-rps16 | 55 | 0.111 |
| rps12-clpP | 53 | 0.105 |
| petA-psbJ | 52 | 0.068 |
| trnV-trnM | 50 | 0.045 |
| ccsA | 45 | 0.06 |
| rpoB | 44 | 0.021 |
| ycf2 | 41 | 0.008 |
| rpl32-trnL | 40 | 0.187 |
| rpoC1 | 35 | 0.026 |
| trnT-psbD | 34 | 0.075 |
| psaA-ycf3 | 33 | 0.095 |
| accD | 33 | 0.032 |
| rpoB-trnC | 31 | 0.071 |
| ycf4-cemA | 31 | 0.088 |
| trnS-G | 29 | 0.098 |
| trnG-intron | 29 | 0.056 |
| rps16-intron | 28 | 0.053 |
| atpI-rps2 | 26 | 0.067 |
| psaB | 26 | 0.017 |
| trnE-T | 25 | 0.088 |
| trnL-ccsA | 25 | 0.084 |
| atpF-intron | 24 | 0.047 |
| atpH-I | 24 | 0.068 |
| psbB | 24 | 0.022 |
| rpoA | 24 | 0.029 |
| atpB-rbcL | 23 | 0.06 |
| trnD-Y | 22 | 0.111 |
| cemA | 22 | 0.043 |
| psbE-petL | 22 | 0.08 |
| clpP-intron2 | 22 | 0.059 |
| ycf3-trnS | 20 | 0.058 |
| psbK-psbI | 19 | 0.093 |
| rpoC1-intron | 19 | 0.055 |
| petN-psbM | 19 | 0.07 |
| psaA | 19 | 0.011 |
| rbcL-accD | 19 | 0.067 |
| petA | 19 | 0.027 |
| psaJ-rpl33 | 19 | 0.09 |
| ndhDψ | 19 | n/a |
| ycf3-intron1 | 18 | 0.033 |
| trnT-L | 18 | 0.094 |
| trnV-intron | 18 | 0.045 |
| rps15-ycf1 | 18 | 0.128 |
| psbA | 17 | 0.02 |
| atpA | 17 | 0.017 |
| rps2 | 17 | 0.029 |
| trnC-petN | 17 | 0.085 |
| psbZ-trnG | 17 | 0.119 |
| rbcL | 17 | 0.015 |
| petD-intron | 17 | 0.049 |
| ndhBψ | 17 | n/a |
| trnL-intron | 16 | 0.051 |
| rps3 | 16 | 0.04 |
| ycf1ψ | 16 | 0.026 |
| atpF | 15 | 0.033 |
| atpI | 15 | 0.03 |
| psbB-psbT | 15 | 0.097 |
| psbC | 14 | 0.016 |
| atpB | 14 | 0.016 |
| trnS-psbZ | 13 | 0.069 |
| clpP-intron1 | 13 | 0.062 |
| petB-intron | 13 | 0.044 |
| trnA-23srRNA | 13 | 0.028 |
| psbA-trnK | 12 | 0.068 |
| atpF-H | 12 | 0.05 |
| trnS-rps4 | 12 | 0.077 |
| psaI-ycf4 | 12 | 0.063 |
| rpl22 | 12 | 0.046 |
| ycf15-trnL | 12 | 0.009 |
| trnH-psbA | 11 | 0.2 |
| psbM-trnD | 11 | 0.058 |
| ycf4 | 11 | 0.027 |
| rpl20 | 11 | 0.027 |
| petB | 11 | 0.019 |
| petD | 11 | 0.021 |
| rpl32 | 11 | 0.077 |
| rps15 | 11 | 0.063 |
| trnQ-psbK | 10 | 0.062 |
| trnG-fM | 10 | 0.064 |
| ycf3-intron2 | 10 | 0.03 |
| trnL-F | 10 | 0.075 |
| accD-psaI | 10 | 0.079 |
| clpP-psbB | 10 | 0.063 |
| rps19 | 10 | 0.033 |
| ndhEψ | 10 | n/a |
| trnR-atpA | 9 | 0.131 |
| psbD | 9 | 0.015 |
| ndhCψ | 8 | n/a |
| petL-petG | 8 | 0.065 |
| rps18-rpl20 | 8 | 0.062 |
| psbH-petB | 8 | 0.061 |
| rps11 | 8 | 0.029 |
| rps8 | 8 | 0.028 |
| trnM-atpE | 7 | 0.078 |
| atpE | 7 | 0.028 |
| petB-petD | 7 | 0.055 |
| rpl16 | 7 | 0.036 |
| psaC | 7 | 0.025 |
| psbI-trnS | 6 | 0.109 |
| trnG-R | 6 | 0.119 |
| rps2-rpoC2 | 6 | 0.075 |
| infA-rps8 | 6 | 0.047 |
| rpl14 | 6 | 0.031 |
| rps16 | 5 | 0.034 |
| atpH | 5 | 0.018 |
| rpoC2-rpoC1 | 5 | 0.076 |
| psbC-trnS | 5 | 0.065 |
| trnfM-rps14 | 5 | 0.051 |
| rps14 | 5 | 0.021 |
| rps4 | 5 | 0.017 |
| ndhKψ | 5 | n/a |
| cemA-petA | 5 | 0.069 |
| trnP-psaJ | 5 | 0.05 |
| rps18 | 5 | 0.023 |
| clpP | 5 | 0.02 |
| rpl36-infA | 5 | 0.076 |
| rpl2 | 5 | 0.007 |
| ndhFψ | 5 | n/a |
| ndhAψ | 5 | n/a |
| ycf3 | 4 | 0.012 |
| rps4-trnT | 4 | 0.065 |
| psbE | 4 | 0.013 |
| petG | 4 | 0.023 |
| rpl33 | 4 | 0.032 |
| rps8-rpl14 | 4 | 0.073 |
| trnR-trnN | 4 | 0.017 |
| trnY-E | 3 | 0.015 |
| petG-trnW | 3 | 0.083 |
| psbT-psbN | 3 | 0.102 |
| infA | 3 | 0.023 |
| rps3-rpl22 | 3 | 0.137 |
| rpl22-rps19 | 3 | 0.055 |
| rpl23 | 3 | 0.006 |
| rps7-rps12 | 3 | 0.02 |
| trnH | 2 | 0.027 |
| trnQ | 2 | 0.028 |
| trnS | 2 | 0.014 |
| atpA-F | 2 | 0.046 |
| trnT | 2 | 0.021 |
| psbZ | 2 | 0.023 |
| psaI | 2 | 0.015 |
| psbL | 2 | 0.018 |
| petL | 2 | 0.025 |
| rpl33-rps18 | 2 | 0.069 |
| petD-rpoA | 2 | 0.065 |
| rpl16-rps3 | 2 | 0.096 |
| rps12 | 2 | 0.01 |
| trnV-16sRNA | 2 | 0.014 |
| trnI-intron | 2 | 0.005 |
| trnI-trnA | 2 | 0.009 |
| 4.5srRNA-5srRNA | 2 | 0.019 |
| trnN-ycf1ψ | 2 | 0.024 |
| ndhIψ | 2 | n/a |
| trnK | 1 | 0.01 |
| rps16-trnQ | 1 | 0.119 |
| psbK | 1 | 0.03 |
| trnR | 1 | 0.01 |
| petN | 1 | 0.018 |
| psbM | 1 | 0.013 |
| trnD | 1 | 0.008 |
| trnT | 1 | 0.01 |
| trnF | 1 | 0.01 |
| psbJ-psbL | 1 | 0.02 |
| trnW-trnP | 1 | 0.079 |
| psaJ | 1 | 0.008 |
| psbT | 1 | 0.013 |
| psbN-psbH | 1 | 0.038 |
| rps11-rpl36 | 1 | 0.03 |
| rpl36 | 1 | 0.014 |
| rpl14-rpl16 | 1 | 0.054 |
| rpl23-trnI | 1 | 0.024 |
| ycf15 | 1 | 0.005 |
| rps7 | 1 | 0.003 |
| 16sRNA | 1 | 0.001 |
| 16sRNA-trnI | 1 | 0.004 |
| ycf68ψ | 1 | 0.006 |
| trnA | 1 | 0.014 |
| 5srRNA | 1 | 0.005 |
| 5srRNA-trnR | 1 | 0.017 |
| trnL | 1 | 0 |
| psbI | 0 | 0.015 |
| trnG | 0 | 0.003 |
| rpoC1-rpoB | 0 | 0.057 |
| trnC | 0 | 0.004 |
| trnY | 0 | 0 |
| trnE | 0 | 0 |
| trnS | 0 | 0.003 |
| trnG | 0 | 0.004 |
| trnfM | 0 | 0 |
| rps14-psaB | 0 | 0.036 |
| psaB-psaA | 0 | 0 |
| trnS | 0 | 0.007 |
| trnL | 0 | 0.014 |
| trnV | 0 | 0 |
| trnM | 0 | 0.004 |
| psbJ | 0 | 0.007 |
| psbL-psbF | 0 | 0.046 |
| psbF | 0 | 0.01 |
| psbF-psbE | 0 | 0.02 |
| trnW | 0 | 0.004 |
| trnP | 0 | 0 |
| psbN | 0 | 0.011 |
| psbH | 0 | 0.017 |
| rpoA-rps11 | 0 | 0.062 |
| rpl19-rpl2 | 0 | 0.045 |
| rpl2-intron | 0 | 0.005 |
| rpl2-rpl23 | 0 | 0.032 |
| trnI | 0 | 0 |
| trnI-ycf2 | 0 | 0 |
| ycf2-ycf15 | 0 | 0 |
| trnL | 0 | 0 |
| rps12-intron | 0 | 0.004 |
| trnV | 0 | 0.004 |
| trnI | 0 | 0 |
| orf56ψ | 0 | 0.007 |
| orf42ψ | 0 | 0.012 |
| trnA-intron | 0 | 0.003 |
| 23srRNA-4.5srRNA | 0 | 0.009 |
| 23srRNA | 0 | 0 |
| 4.5srRNA | 0 | 0 |
| trnR | 0 | 0 |
| trnN | 0 | 0 |
| trnF-ndhJψ | n/a | n/a |
| ndhJψ | n/a | n/a |
| ndhCψ-trnV | n/a | n/a |
| trnL-ndhBψ | n/a | n/a |
| ndhBψ-rps7 | n/a | n/a |
| ycf1ψ-ndhFψ | n/a | n/a |
| ndhFψ-rpl32 | n/a | n/a |
| ccsA-ndhDψ | n/a | n/a |
| ndhDψ-psaC | n/a | n/a |
| psaC-ndhEψ | n/a | n/a |
| ndhEψ-ndhGψ | n/a | n/a |
| ndhGψ | n/a | n/a |
| ndhGψ-ndhIψ | n/a | n/a |
| ndhIψ-ndhAψ | n/a | n/a |
| ndhAψndhHψ | n/a | n/a |
| ndhHψ | n/a | n/a |
| ndhHψ-rps15 | n/a | n/a |
