# Supplementary material for: Comparative genomic analysis of Genlisea (corkscrew plants—Lentibulariaceae) chloroplast genomes reveals an increasing loss of the ndh genes
Source: PLoS One. 2018 Jan 2;13(1):e0190321. doi: 10.1371/journal.pone.0190321 (PMC5749785; doi:10.1371/journal.pone.0190321)
Supplement: S7 Table — (DOCX) [file pone.0190321.s012.docx]

| **S7 Table. Codon usage and amino acid frequencies for *Genlisea* plastomes.** | | | | | | | | | | | |
| --- | --- | --- | --- | --- | --- | --- | --- | --- | --- | --- | --- |
| **Codon usage and amino acid frequencies for *Genlisea aurea*** | | | | | | | | | | | |
| **Amino acid** | **Codon** | **No.** | **RSCU (a)** | **%(b)** | **tRNA** | **Amino acid** | **Codon** | **No.** | **RSCU (a)** | **%(b)** | **tRNA** |
| Ala | GCU | 456 | 1.69 | 42.14 | - | Pro | CCU | 292 | 1.44 | 35.92 | - |
|  | GCC | 198 | 0.73 | 18.30 | - |  | CCC | 155 | 0.76 | 19.07 | - |
|  | GCA | 288 | 1.06 | 26.62 | trnA-UGC* |  | CCA | 234 | 1.15 | 28.78 | trnP-UGG |
|  | GCG | 140 | 0.52 | 12.94 | - |  | CCG | 132 | 0.65 | 16.24 | - |
| Cys | UGU | 167 | 1.57 | 78.40 | - | Gln | CAA | 565 | 1.53 | 76.35 | trnQ-UUG |
|  | UGC | 46 | 0.43 | 21.60 | trnC-GCA |  | CAG | 175 | 0.47 | 23.65 | - |
| Asp | GAU | 593 | 1.59 | 79.28 | - | Arg | AGA | 347 | 1.72 | 27.47 | trnR-UCU |
|  | GAC | 155 | 0.41 | 20.72 | trnD-GUC |  | AGG | 124 | 0.61 | 9.82 | - |
| Glu | GAA | 771 | 1.53 | 76.26 | trnE-UUC |  | CGU | 243 | 1.20 | 19.24 | trnR-ACG |
|  | GAG | 240 | 0.47 | 23.74 | - |  | CUC | 134 | 0.40 | 10.61 | - |
| Phe | UUU | 732 | 1.38 | 68.8 | - |  | CUA | 278 | 0.84 | 22.01 | - |
|  | UUC | 332 | 0.62 | 31.2 | trnF-GAA |  | CUG | 137 | 0.41 | 10.85 | - |
| Gly | GGU | 442 | 1.33 | 33.18 | - | Ser | UCU | 379 | 1.61 | 26.86 | - |
|  | GGC | 157 | 0.47 | 11.79 | trnG-GCC |  | UCC | 214 | 0.91 | 15.17 | trnS-GGA |
|  | GGA | 489 | 1.47 | 36.71 | trnG-UCC* |  | UCA | 259 | 1.10 | 18.36 | trnS-UGA |
|  | GGG | 244 | 0.73 | 18.32 | - |  | UCG | 171 | 0.73 | 12.12 | - |
| His | CAU | 370 | 1.52 | 75.82 | - |  | AGU | 304 | 1.29 | 21.55 | - |
|  | CAC | 118 | 0.48 | 24.18 | trnH-GUG |  | AGC | 84 | 0.36 | 5.95 | trnS-GCU |
| Ile | AUU | 800 | 1.55 | 51.51 | - | Thr | ACU | 407 | 1.57 | 39.25 | - |
|  | AUC | 302 | 0.58 | 19.45 | trnI-GAU |  | ACC | 201 | 0.78 | 19.38 | trnT-GGU |
|  | AUA | 451 | 0.87 | 29.04 | trnI-CAU* |  | ACA | 297 | 1.15 | 28.64 | trnT-UGU |
| Lys | AAA | 822 | 1.53 | 76.25 | trnK-UUU* |  | ACG | 132 | 0.51 | 12.73 | - |
|  | AAG | 256 | 0.47 | 23.75 |  | Val | GUU | 402 | 1.48 | 37.12 | - |
| Leu | UUA | 617 | 1.86 | 31.01 | trnL-UAA* |  | GUC | 146 | 0.54 | 13.48 | trnV-GAC |
|  | UUG | 412 | 1.24 | 20.7 | trnL-CAA |  | GUA | 399 | 1.47 | 36.84 | trnV-UAC* |
|  | CUU | 412 | 1.24 | 20.7 | - |  | GUG | 136 | 0.50 | 12.56 | - |
|  | CUC | 134 | 0.40 | 6.734 | - | Tyr | UAU | 532 | 1.61 | 80.48 | - |
|  | CUA | 278 | 0.84 | 13.97 | trnL-UAG |  | UAC | 129 | 0.39 | 19.52 | trnY-GUA |
|  | CUG | 137 | 0.41 | 6.884 | - | Trp | UGG | 334 | 1.00 | 100 | trnW-CCA |
| Met (START) | AUG | 424 | 1.00 | 100 | trnf(f)M-CAU | STOP | UGA | 15 | 0.65 | 21.74 | - |
| Asn | AAU | 702 | 1.49 | 74.52 | - |  | UAA | 36 | 1.57 | 66.67 | - |
|  | AAC | 240 | 0.51 | 25.48 | trnN-GUU |  | UAG | 18 | 0.78 | 33.33 | - |

(a) Relative Synonymous Codon Usage

(b) Codon frequency (in %) per amino acid

***Intron-containing tRNA genes

**Codon usage and amino acid frequencies for *Genlisea filiformis***

| **Amino acid** | **Codon** | **No.** | **RSCU (a)** | **% (b)** | **tRNA** | **Amino acid** | **Codon** | **No.** | **RSCU (a)** | **% (b)** | **tRNA** |
| --- | --- | --- | --- | --- | --- | --- | --- | --- | --- | --- | --- |
| Ala | GCU | 454 | 1.71 | 42.71 | - | Pro | CCU | 270 | 1.42 | 35.53 | - |
|  | GCC | 197 | 0.74 | 18.53 | - |  | CCC | 150 | 0.79 | 19.74 | - |
|  | GCA | 277 | 1.04 | 26.06 | trnA-UGC* |  | CCA | 218 | 1.15 | 28.68 | trnP-UGG |
|  | GCG | 135 | 0.51 | 12.7 | - |  | CCG | 122 | 0.64 | 16.05 | - |
| Cys | UGU | 152 | 1.54 | 76.77 | - | Gln | CAA | 488 | 1.51 | 75.31 | trnQ-UUG |
|  | UGC | 46 | 0.46 | 23.23 | trnC-GCA |  | CAG | 160 | 0.49 | 24.69 | - |
| Asp | GAU | 546 | 1.58 | 78.9 | - | Arg | CGU | 234 | 1.24 | 20.73 | trnR-ACG |
|  | GAC | 146 | 0.42 | 21.1 | trnD-GUC |  | CGC | 91 | 0.48 | 8.06 | - |
| Glu | GAA | 681 | 1.50 | 75 | trnE-UUC |  | CGA | 274 | 1.46 | 24.27 | - |
|  | GAG | 227 | 0.50 | 25 | - |  | CGG | 102 | 0.54 | 9.03 | - |
| Phe | UUU | 634 | 1.34 | 66.95 | - |  | AGA | 314 | 1.67 | 27.81 | trnR-UCU |
|  | UUC | 313 | 0.66 | 33.05 | trnF-GAA |  | AGG | 114 | 0.61 | 10.10 | - |
| Gly | GGU | 434 | 1.35 | 33.77 | - | Ser | UCU | 357 | 1.69 | 28.09 | - |
|  | GGC | 153 | 0.48 | 11.91 | trnG-GCC |  | UCC | 199 | 0.94 | 15.66 | trnS-GGA |
|  | GGA | 463 | 1.44 | 36.03 | trnG-UCC* |  | UCA | 217 | 1.02 | 17.07 | trnS-UGA |
|  | GGG | 235 | 0.73 | 18.29 | - |  | UCG | 150 | 0.71 | 11.80 | - |
| His | CAU | 353 | 1.54 | 77.07 | - |  | AGU | 271 | 1.28 | 21.32 | - |
|  | CAC | 105 | 0.46 | 22.93 | trnH-GUG |  | AGC | 77 | 0.36 | 6.06 | trnS-GCU |
| Ile | AUU | 728 | 1.54 | 51.45 | - | Thr | ACU | 362 | 1.55 | 38.68 | - |
|  | AUC | 286 | 0.61 | 20.21 | trnI-GAU |  | ACC | 200 | 0.85 | 21.37 | trnT-GGU |
|  | AUA | 401 | 0.85 | 28.34 | trnI-CAU* |  | ACA | 256 | 1.09 | 27.35 | trnT-UGU |
| Lys | AAA | 645 | 1.49 | 74.48 | trnK-UUU* |  | ACG | 118 | 0.50 | 12.61 | - |
|  | AAG | 221 | 0.51 | 25.52 |  | Val | GUU | 379 | 1.46 | 36.44 | - |
| Leu | UUA | 574 | 1.87 | 31.25 | trnL-UAA* |  | GUC | 148 | 0.57 | 14.23 | trnV-GAC |
|  | UUG | 377 | 1.23 | 20.52 | trnL-CAA |  | GUA | 381 | 1.47 | 36.63 | trnV-UAC* |
|  | CUU | 376 | 1.23 | 20.47 | - |  | GUG | 132 | 0.51 | 12.69 | - |
|  | CUC | 119 | 0.39 | 6.478 | - | Tyr | UAU | 487 | 1.61 | 80.63 | - |
|  | CUA | 262 | 0.86 | 14.26 | trnL-UAG |  | UAC | 117 | 0.39 | 19.37 | trnY-GUA |
|  | CUG | 129 | 0.42 | 7.022 | - | Trp | UGG | 313 | 1.00 | 100 | trnW-CCA |
| Met (START) | AUG | 397 | 1.00 | 100 | trnf(f)M-CAU | STOP | UGA | 17 | 0.75 | 25.00 | - |
| Asn | AAU | 603 | 1.47 | 73.63 | - |  | UAA | 32 | 1.41 | 62.75 | - |
|  | AAC | 216 | 0.53 | 26.37 | trnN-GUU |  | UAG | 19 | 0.84 | 37.25 | - |

(a) Relative Synonymous Codon Usage

(b) Codon frequency (in %) per amino acid

***Intron-containing tRNA genes

**Codon usage and amino acid frequencies for *Genlisea pygmaea.***

| **Amino acid** | **Codon** | **No.** | **RSCU (a)** | **% (b)** | **tRNA** | **Amino acid** | **Codon** | **No.** | **RSCU (a)** | **% (b)** | **tRNA** |
| --- | --- | --- | --- | --- | --- | --- | --- | --- | --- | --- | --- |
| Ala | GCU | 456 | 1.70 | 42.62 | - | Pro | CCU | 297 | 1.45 | 36.31 | - |
|  | GCC | 199 | 0.74 | 18.6 | - |  | CCC | 151 | 0.74 | 18.46 | - |
|  | GCA | 277 | 1.04 | 25.89 | trnA-UGC* |  | CCA | 241 | 1.18 | 29.46 | trnP-UGG |
|  | GCG | 138 | 0.52 | 12.9 | - |  | CCG | 129 | 0.63 | 15.77 | - |
| Cys | UGU | 164 | 1.56 | 78.1 | - | Gln | CAA | 558 | 1.53 | 76.44 | trnQ-UUG |
|  | UGC | 46 | 0.44 | 21.9 | trnC-GCA |  | CAG | 172 | 0.47 | 23.56 | - |
| Asp | GAU | 591 | 1.58 | 79.22 | - | Arg | CGU | 247 | 1.22 | 72.43 | trnR-UCU |
|  | GAC | 155 | 0.42 | 20.78 | trnD-GUC |  | CGC | 94 | 0.47 | 27.57 | - |
| Glu | GAA | 764 | 1.52 | 75.94 | trnE-UUC |  | CGA | 288 | 1.43 | 73.28 | trnR-ACG |
|  | GAG | 242 | 0.48 | 24.06 | - |  | CGG | 105 | 0.52 | 26.72 | - |
| Phe | UUU | 718 | 1.37 | 68.38 | - |  | AGA | 348 | 1.73 | 73.11 | - |
|  | UUC | 332 | 0.63 | 31.62 | trnF-GAA |  | AGG | 128 | 0.63 | 26.89 | - |
| Gly | GGU | 446 | 1.33 | 33.33 | - | Ser | UCU | 377 | 1.64 | 37.62 | - |
|  | GGC | 156 | 0.47 | 11.66 | trnG-GCC |  | UCC | 214 | 0.93 | 21.36 | trnS-GGA |
|  | GGA | 486 | 1.45 | 36.32 | trnG-UCC* |  | UCA | 245 | 1.06 | 24.45 | trnS-UGA |
|  | GGG | 250 | 0.75 | 18.68 | - |  | UCG | 166 | 0.72 | 16.57 | - |
| His | CAU | 367 | 1.51 | 75.67 | - |  | AGU | 297 | 1.29 | 78.36 | - |
|  | CAC | 118 | 0.49 | 24.33 | trnH-GUG |  | AGC | 82 | 0.36 | 21.64 | trnS-GCU |
| Ile | AUU | 785 | 1.52 | 50.61 | - | Thr | ACU | 390 | 1.54 | 44.32 | - |
|  | AUC | 314 | 0.61 | 20.25 | trnI-GAU |  | ACC | 201 | 0.79 | 22.84 | trnT-GGU |
|  | AUA | 452 | 0.87 | 29.14 | trnI-CAU* |  | ACA | 289 | 1.14 | 32.84 | trnT-UGU |
| Lys | AAA | 799 | 1.52 | 75.88 | trnK-UUU* |  | ACG | 136 | 0.54 | 25.52 | - |
|  | AAG | 254 | 0.48 | 24.12 |  | Val | GUU | 397 | 1.47 | 74.48 | - |
| Leu | UUA | 619 | 1.86 | 31.06 | trnL-UAA* |  | GUC | 143 | 0.53 | 8.58 | trnV-GAC |
|  | UUG | 415 | 1.25 | 20.82 | trnL-CAA |  | GUA | 402 | 1.49 | 24.13 | trnV-UAC* |
|  | CUU | 412 | 1.24 | 20.67 | - |  | GUG | 136 | 0.50 | 8.16 | - |
|  | CUC | 130 | 0.39 | 6.523 | - | Tyr | UAU | 529 | 1.62 | 31.75 | - |
|  | CUA | 276 | 0.83 | 13.85 | trnL-UAG |  | UAC | 124 | 0.38 | 7.44 | trnY-GUA |
|  | CUG | 141 | 0.42 | 7.075 | - | Trp | UGG | 332 | 1.00 | 19.93 | trnW-CCA |
| Met (START) | AUG | 415 | 1.00 | 100 | trnf(f)M-CAU | STOP | UGA | 16 | 0.71 | 100 | - |
| Asn | AAU | 692 | 1.48 | 73.85 | - |  | UAA | 33 | 1.46 | 63.46 | - |
|  | AAC | 245 | 0.52 | 26.15 | trnN-GUU |  | UAG | 19 | 0.84 | 36.54 | - |

(a) Relative Synonymous Codon Usage

(b) Codon frequency (in %) per amino acid

***Intron-containing tRNA genes

**Codon usage and amino acid frequencies for *Genlisea repens.***

| **Amino acid** | **Codon** | **No.** | **RSCU (a)** | **% (b)** | **tRNA** | **Amino acid** | **Codon** | **No.** | **RSCU (a)** | **% (b)** | **tRNA** |
| --- | --- | --- | --- | --- | --- | --- | --- | --- | --- | --- | --- |
| Ala | GCU | 460 | 1.70 | 42.51 | - | Pro | CCU | 298 | 1.45 | 36.30 | - |
|  | GCC | 200 | 0.74 | 18.48 | - |  | CCC | 152 | 0.74 | 18.51 | - |
|  | GCA | 283 | 1.05 | 26.16 | trnA-UGC* |  | CCA | 242 | 1.18 | 29.48 | trnP-UGG |
|  | GCG | 139 | 0.51 | 12.85 | - |  | CCG | 129 | 0.63 | 15.71 | - |
| Cys | UGU | 167 | 1.55 | 77.67 | - | Gln | CAA | 556 | 1.53 | 76.37 | trnQ-UUG |
|  | UGC | 48 | 0.45 | 22.33 | trnC-GCA |  | CAG | 172 | 0.47 | 23.63 | - |
| Asp | GAU | 597 | 1.58 | 79.18 | - | Arg | AGA | 350 | 1.73 | 73.22 | trnR-UCU |
|  | GAC | 157 | 0.42 | 20.82 | trnD-GUC |  | AGG | 128 | 0.63 | 26.78 | - |
| Glu | GAA | 770 | 1.52 | 75.94 | trnE-UUC |  | CGU | 244 | 1.21 | 71.98 | trnR-ACG |
|  | GAG | 244 | 0.48 | 24.06 | - |  | CGC | 95 | 0.47 | 28.02 | - |
| Phe | UUU | 723 | 1.37 | 68.47 | - |  | CGA | 288 | 1.42 | 72.54 | - |
|  | UUC | 333 | 0.63 | 31.53 | trnF-GAA |  | CGG | 109 | 0.54 | 27.46 | - |
| Gly | GGU | 449 | 1.34 | 33.41 | - | Ser | UCU | 378 | 1.63 | 37.35 | - |
|  | GGC | 155 | 0.46 | 11.53 | trnG-GCC |  | UCC | 219 | 0.94 | 21.64 | trnS-GGA |
|  | GGA | 488 | 1.45 | 36.31 | trnG-UCC* |  | UCA | 248 | 1.07 | 24.51 | trnS-UGA |
|  | GGG | 252 | 0.75 | 18.75 | - |  | UCG | 167 | 0.72 | 16.50 | - |
| His | CAU | 370 | 1.52 | 75.98 | - |  | AGU | 299 | 1.29 | 78.48 | - |
|  | CAC | 117 | 0.48 | 24.02 | trnH-GUG |  | AGC | 82 | 0.35 | 21.52 | trnS-GCU |
| Ile | AUU | 792 | 1.52 | 50.64 | - | Thr | ACU | 396 | 1.55 | 44.54 | - |
|  | AUC | 314 | 0.60 | 20.08 | trnI-GAU |  | ACC | 203 | 0.79 | 22.83 | trnT-GGU |
|  | AUA | 458 | 0.88 | 29.28 | trnI-CAU* |  | ACA | 290 | 1.13 | 32.62 | trnT-UGU |
| Lys | AAA | 810 | 1.52 | 76.13 | trnK-UUU* |  | ACG | 134 | 0.52 | 25.28 | - |
|  | AAG | 254 | 0.48 | 23.87 |  | Val | GUU | 396 | 1.46 | 74.72 | - |
| Leu | UUA | 623 | 1.86 | 31.06 | trnL-UAA* |  | GUC | 146 | 0.54 | 8.68 | trnV-GAC |
|  | UUG | 416 | 1.24 | 20.74 | trnL-CAA |  | GUA | 405 | 1.49 | 24.08 | trnV-UAC* |
|  | CUU | 413 | 1.24 | 20.59 | - |  | GUG | 137 | 0.51 | 8.15 | - |
|  | CUC | 129 | 0.39 | 6.431 | - | Tyr | UAU | 538 | 1.63 | 31.99 | - |
|  | CUA | 283 | 0.85 | 14.11 | trnL-UAG |  | UAC | 124 | 0.37 | 7.37 | trnY-GUA |
|  | CUG | 142 | 0.42 | 7.079 | - | Trp | UGG | 332 | 1.00 | 19.74 | trnW-CCA |
| Met (START) | AUG | 417 | 1.00 | 100 | trnf(f)M-CAU | STOP | UGA | 16 | 0.70 | 100 | - |
| Asn | AAU | 695 | 1.48 | 73.78 | - |  | UAA | 34 | 1.48 | 64.15 | - |
|  | AAC | 247 | 0.52 | 26.22 | trnN-GUU |  | UAG | 19 | 0.83 | 35.85 | - |

(a) Relative Synonymous Codon Usage

(b) Codon frequency (in %) per amino acid

***Intron-containing tRNA genes

**Codon usage and amino acid frequencies for *Genlisea tuberosa.***

| **Amino acid** | **Codon** | **No.** | **RSCU (a)** | **% (b)** | **tRNA** | **Amino acid** | **Codon** | **No.** | **RSCU (a)** | **% (b)** | **tRNA** |
| --- | --- | --- | --- | --- | --- | --- | --- | --- | --- | --- | --- |
| Ala | GCU | 454 | 1.68 | 41.96 | - | Pro | CCU | 294 | 1.44 | 36.12 | - |
|  | GCC | 199 | 0.74 | 18.39 | - |  | CCC | 155 | 0.76 | 19.04 | - |
|  | GCA | 289 | 1.07 | 26.71 | trnA-UGC* |  | CCA | 234 | 1.15 | 28.75 | trnP-UGG |
|  | GCG | 140 | 0.52 | 12.94 | - |  | CCG | 131 | 0.64 | 16.09 | - |
| Cys | UGU | 163 | 1.55 | 77.25 | - | Gln | CAA | 562 | 1.53 | 76.36 | trnQ-UUG |
|  | UGC | 48 | 0.45 | 22.75 | trnC-GCA |  | CAG | 174 | 0.47 | 23.64 | - |
| Asp | GAU | 596 | 1.59 | 79.47 | - | Arg | CGU | 241 | 1.19 | 19.87 | trnR-UCU |
|  | GAC | 154 | 0.41 | 20.53 | trnD-GUC |  | CGC | 98 | 0.48 | 8.08 | - |
| Glu | GAA | 771 | 1.52 | 76.04 | trnE-UUC |  | CGA | 292 | 1.44 | 24.07 | trnR-ACG |
|  | GAG | 243 | 0.48 | 23.96 | - |  | CGG | 106 | 0.52 | 8.74 | - |
| Phe | UUU | 732 | 1.38 | 68.99 | - |  | AGA | 348 | 1.72 | 28.69 | - |
|  | UUC | 329 | 0.62 | 31.01 | trnF-GAA |  | AGG | 128 | 0.63 | 10.55 | - |
| Gly | GGU | 440 | 1.32 | 33.06 | - | Ser | UCU | 371 | 1.58 | 26.29 | - |
|  | GGC | 162 | 0.49 | 12.17 | trnG-GCC |  | UCC | 218 | 0.93 | 15.45 | trnS-GGA |
|  | GGA | 486 | 1.46 | 36.51 | trnG-UCC* |  | UCA | 260 | 1.11 | 18.43 | trnS-UGA |
|  | GGG | 243 | 0.73 | 18.26 | - |  | UCG | 171 | 0.73 | 12.12 | - |
| His | CAU | 372 | 1.52 | 75.92 | - |  | AGU | 304 | 1.29 | 21.55 | - |
|  | CAC | 118 | 0.48 | 24.08 | trnH-GUG |  | AGC | 87 | 0.37 | 6.17 | trnS-GCU |
| Ile | AUU | 799 | 1.54 | 51.42 | - | Thr | ACU | 407 | 1.57 | 39.21 | - |
|  | AUC | 310 | 0.60 | 19.95 | trnI-GAU |  | ACC | 201 | 0.77 | 19.36 | trnT-GGU |
|  | AUA | 445 | 0.86 | 28.64 | trnI-CAU* |  | ACA | 296 | 1.14 | 28.52 | trnT-UGU |
| Lys | AAA | 819 | 1.53 | 76.33 | trnK-UUU* |  | ACG | 134 | 0.52 | 12.91 | - |
|  | AAG | 254 | 0.47 | 23.67 |  | Val | GUU | 403 | 1.49 | 37.25 | - |
| Leu | UUA | 623 | 1.88 | 31.34 | trnL-UAA* |  | GUC | 148 | 0.55 | 13.68 | trnV-GAC |
|  | UUG | 409 | 1.23 | 20.57 | trnL-CAA |  | GUA | 398 | 1.47 | 36.78 | trnV-UAC* |
|  | CUU | 412 | 1.24 | 20.72 | - |  | GUG | 133 | 0.49 | 12.29 | - |
|  | CUC | 133 | 0.40 | 6.69 | - | Tyr | UAU | 540 | 1.62 | 80.96 | - |
|  | CUA | 276 | 0.83 | 13.88 | trnL-UAG |  | UAC | 127 | 0.38 | 19.04 | trnY-GUA |
|  | CUG | 135 | 0.41 | 6.791 | - | Trp | UGG | 335 | 1.00 | 100 | trnW-CCA |
| Met (START) | AUG | 424 | 1.00 | 100 | trnf(f)M-CAU | STOP | UGA | 16 | 0.70 | 23.19 | - |
| Asn | AAU | 698 | 1.49 | 74.33 | - |  | UAA | 35 | 1.52 | 50.72 | - |
|  | AAC | 241 | 0.51 | 25.67 | trnN-GUU |  | UAG | 18 | 0.78 | 26.09 | - |

(a) Relative Synonymous Codon Usage

(b) Codon frequency (in %) per amino acid

***Intron-containing tRNA genes

**Codon usage and amino acid frequencies for *Genlisea violacea.***

| **Amino acid** | **Codon** | **No.** | **RSCU (a)** | **% (b)** | **tRNA** | **Amino acid** | **Codon** | **No.** | **RSCU (a)** | **% (b)** | **tRNA** |
| --- | --- | --- | --- | --- | --- | --- | --- | --- | --- | --- | --- |
| Ala | GCU | 458 | 1.72 | 43.05 | - | Pro | CCU | 293 | 1.46 | 36.49 | - |
|  | GCC | 179 | 0.67 | 16.82 | - |  | CCC | 172 | 0.86 | 21.42 | - |
|  | GCA | 300 | 1.13 | 28.2 | trnA-UGC* |  | CCA | 222 | 1.11 | 27.65 | trnP-UGG |
|  | GCG | 127 | 0.48 | 11.94 | - |  | CCG | 116 | 0.58 | 14.45 | - |
| Cys | UGU | 154 | 1.51 | 75.49 | - | Gln | CAA | 552 | 1.51 | 75.31 | trnQ-UUG |
|  | UGC | 50 | 0.49 | 24.51 | trnC-GCA |  | CAG | 181 | 0.49 | 24.69 | - |
| Asp | GAU | 605 | 1.56 | 78.17 | - | Arg | AGA | 354 | 1.75 | 29.21 | trnR-UCU |
|  | GAC | 169 | 0.44 | 21.83 | trnD-GUC |  | AGG | 133 | 0.66 | 10.97 | - |
| Glu | GAA | 781 | 1.53 | 76.57 | trnE-UUC |  | CGU | 251 | 1.24 | 20.71 | trnR-ACG |
|  | GAG | 239 | 0.47 | 23.43 | - |  | CGC | 85 | 0.42 | 7.01 | - |
| Phe | UUU | 716 | 1.34 | 67.23 | - |  | CGA | 285 | 1.41 | 23.51 | - |
|  | UUC | 349 | 0.66 | 32.77 | trnF-GAA |  | CGG | 104 | 0.51 | 8.58 | - |
| Gly | GGU | 428 | 1.28 | 31.89 | - | Ser | UCU | 395 | 1.65 | 27.49 | - |
|  | GGC | 158 | 0.47 | 11.77 | trnG-GCC |  | UCC | 233 | 0.97 | 16.21 | trnS-GGA |
|  | GGA | 502 | 1.50 | 37.41 | trnG-UCC* |  | UCA | 253 | 1.06 | 17.61 | trnS-UGA |
|  | GGG | 254 | 0.76 | 18.93 | - |  | UCG | 161 | 0.67 | 11.20 | - |
| His | CAU | 373 | 1.53 | 76.28 | - |  | AGU | 310 | 1.29 | 21.57 | - |
|  | CAC | 116 | 0.47 | 23.72 | trnH-GUG |  | AGC | 85 | 0.35 | 5.92 | trnS-GCU |
| Ile | AUU | 807 | 1.55 | 51.63 | - | Thr | ACU | 404 | 1.58 | 39.61 | - |
|  | AUC | 306 | 0.59 | 19.58 | trnI-GAU |  | ACC | 195 | 0.76 | 19.12 | trnT-GGU |
|  | AUA | 450 | 0.86 | 28.79 | trnI-CAU* |  | ACA | 290 | 1.14 | 28.43 | trnT-UGU |
| Lys | AAA | 804 | 1.50 | 75.21 | trnK-UUU* |  | ACG | 131 | 0.51 | 12.84 | - |
|  | AAG | 265 | 0.50 | 24.79 |  | Val | GUU | 384 | 1.43 | 35.69 | - |
| Leu | UUA | 600 | 1.82 | 30.41 | trnL-UAA* |  | GUC | 136 | 0.51 | 12.64 | trnV-GAC |
|  | UUG | 425 | 1.29 | 21.54 | trnL-CAA |  | GUA | 403 | 1.50 | 37.45 | trnV-UAC* |
|  | CUU | 420 | 1.28 | 21.29 | - |  | GUG | 153 | 0.57 | 14.22 | - |
|  | CUC | 129 | 0.39 | 6.538 | - | Tyr | UAU | 527 | 1.61 | 80.70 | - |
|  | CUA | 272 | 0.83 | 13.79 | trnL-UAG |  | UAC | 126 | 0.39 | 19.30 | trnY-GUA |
|  | CUG | 127 | 0.39 | 6.437 | - | Trp | UGG | 338 | 1.00 | 100 | trnW-CCA |
| Met (START) | AUG | 422 | 1.00 | 100 | trnf(f)M-CAU | STOP | UGA | 16 | 0.70 | 23.19 | - |
| Asn | AAU | 718 | 1.53 | 76.46 | - |  | UAA | 36 | 1.57 | 52.17 | - |
|  | AAC | 221 | 0.47 | 23.54 | trnN-GUU |  | UAG | 17 | 0.74 | 24.64 | - |

(a) Relative Synonymous Codon Usage

(b) Codon frequency (in %) per amino acid

***Intron-containing tRNA genes
